# Leveraging fragment-based drug discovery to advance 3D scaffolds into potent ligands: application to the histamine H_1_ receptor

**DOI:** 10.1039/d5md01081k

**Published:** 2026-01-29

**Authors:** Tom Dekker, Oscar P. J. van Linden, Herman D. Lim, Mabel E. Dekker, Henry F. Vischer, Rob Leurs, Tiffany van der Meer, Maurice C. M. L. Buzink, David J. Hamilton, Barbara Zarzycka, Elwin Janssen, Maikel Wijtmans, Iwan J. P. de Esch

**Affiliations:** a Division of Medicinal Chemistry, Amsterdam Institute of Molecular and Life Sciences, Faculty of Science, Vrije Universiteit Amsterdam De Boelelaan 1108 1081 HZ Amsterdam The Netherlands i.de.esch@vu.nl

## Abstract

Fragment-based drug discovery (FBDD) often relies on screening simple, 2D aromatic fragments, with the introduction of 3D character typically being reserved for later stages of hit optimization. The use of 3D screening fragments has been limited because of concerns about reduced hit rates and limited availability arising from synthetic accessibility constraints. Consequently, the chemical diversity of screening libraries has been restricted, and the potential of 3D scaffolds remains underexplored. To address these limitations, we developed novel synthetic methodologies for constructing 3D scaffolds and integrated them into FBDD workflows, which we consider an optimal strategy to advance these chemistries into hit optimization programs. Here, we report the screening of a focused 3D fragment library and the identification of a cyclobutane-containing hit for the histamine H_1_ receptor. A subsequent hit exploration yielded high-affinity antagonist 17a (VUF26691, p*K*_i_ = 8.8). Overall, we demonstrate that FBDD is an efficient method to achieve the incorporation of novel (3D) chemistries into biologically active compounds.

## Abstract 2

Fragment-based drug discovery enables translation of novel (3D) chemistries into biologically relevant hit matter, as demonstrated by the identification and optimization of a cyclobutane-based 3D fragment into a potent histamine H_1_ receptor ligand.

## Graphical abstract

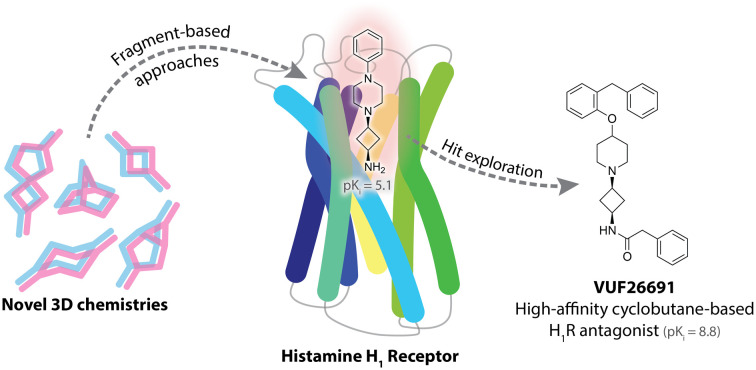

## 1. Introduction

Fragment-based drug discovery (FBDD) has become a well-established approach in drug development, with eight FDA-approved drugs having reached the market as of November 2025 and numerous (pre)clinical candidates under investigation.^1–3^ The success of FBDD is primarily attributed to the structural simplicity of low-molecular-weight (MW ≤ 300 Da) screening compounds.^1,4^ Historically, most fragment libraries consist primarily of structures with relatively many sp^2^-hybridised atoms, as found in aromatic (hetero)cycles.^5–8^ Such structures can be referred to as flat, two-dimensional (2D) entities. Consequently, in recent years, there has been growing interest in the screening of three-dimensional (3D) fragments to improve the overall molecular diversity and broaden the chemical space that is being explored.^5,9–12^ However, since 3D-shaped screening fragments are typically considered to possess greater molecular complexity, they do present a potential contradiction to the core principle of FBDD, which stipulates that hit rates decrease as the size and complexity of the screening compounds increase. Thus, lower hit rates may be expected when 3D fragments are screened, potentially requiring larger screening libraries.^4^ Additional concerns include the synthetic challenges posed by 3D fragments, particularly when stereocenters are introduced. Regardless, the limited presence of 3D molecules in current fragment libraries implies that the respective part of chemical space remains relatively underexplored. Incorporating 3D structures into fragment libraries addresses this gap, but efficient hit exploration and optimization remain dependent on tractable synthetic methodologies.^13,14^ Significant efforts have been made in this direction,^12,15,16^ and as part of our ongoing FBDD efforts, we have also been developing chemistries around 3D fragments to provide access to new chemical space.^17–19^ In our view, FBDD provides an optimal platform for integrating innovative chemistries into initial hit molecules and subsequent drug development programs. This synergy between innovative chemistries and FBDD ensures that novel chemical scaffolds have the highest chance of being identified as hits in drug discovery projects, hence exploiting the chemistries and scaffolds effectively and addressing both structural diversity and ligand sociability. In addition to expanding accessible chemical space, 3D fragments may offer other advantages during hit optimization. Although these findings have also been challenged,^20,21^ an increased 3D character of drug-like molecules has been associated with increased solubility, higher clinical success rates and reduced promiscuity.^22,23^ Furthermore, because the core scaffold of hit fragments is often maintained throughout hit and lead optimization,^3^ using 3D fragments reduces the need to incorporate 3D features at later stages.

Here we report the use of 3D fragments in a hit finding and exploration endeavor for the histamine H_1_ receptor (H_1_R). The histamine receptor family belongs to the class A of G protein-coupled receptors (GPCRs) and is comprised of histamine receptors H_1_R, H_2_R, H_3_R and H_4_R, that all have histamine as the endogenous ligand. H_1_R is primarily involved in allergy and inflammation, and it has been the target for more than 45 anti-allergic and anti-inflammatory drugs that have been developed over the past century.^24^ In 2011, the X-ray structure of H_1_R in complex with the first-generation antihistamine doxepin was published, which has significantly increased the structural understanding of the receptor.^25^ More recently, in 2021 and 2024, cryo-EM structures of H_1_R in the apo form, in complex with histamine/G_q_ protein and with various antihistamines (mepyramine, desloratadine, and astemizole), have been published.^26,27^ Thus, despite the many marketed antihistaminic drugs, H_1_R remains a target that is being studied intensively.

## 2. Results and discussion

### 2.1. Hit finding and exploration

**Fig. 1:**
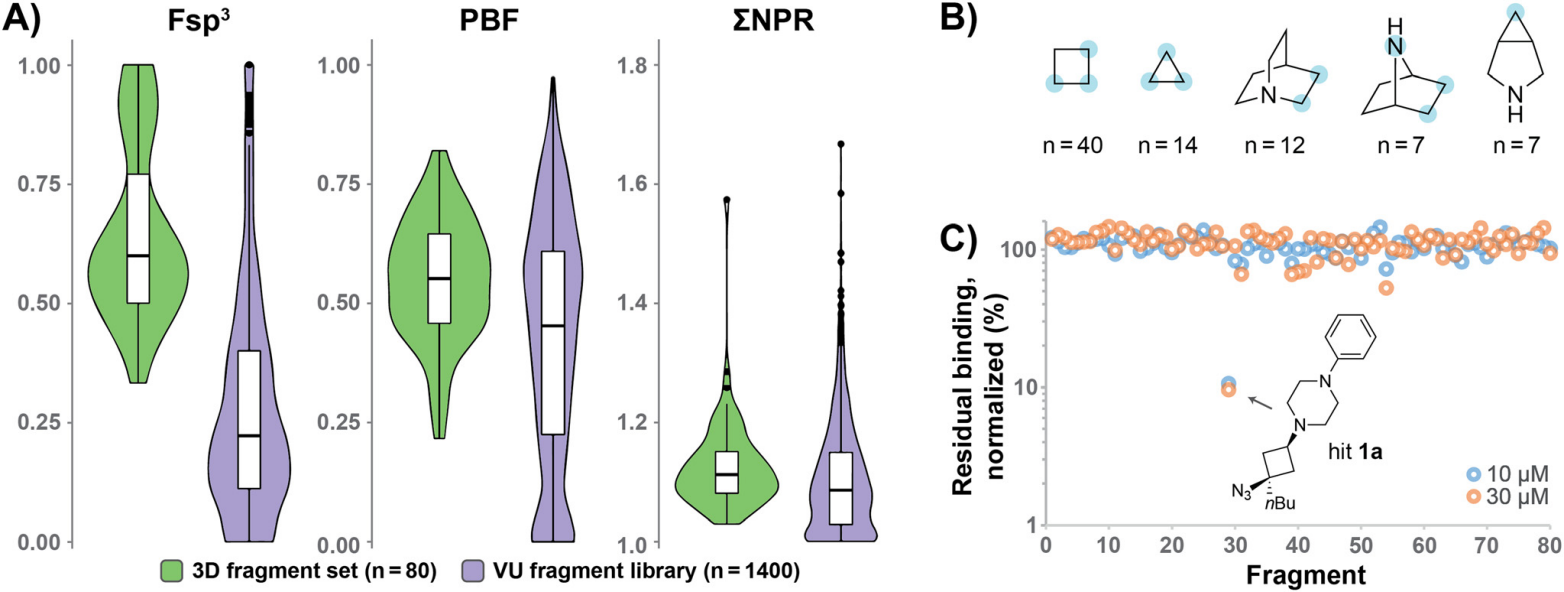
(A) Comparison of the recently added 3D fragment subset (*N* = 80, green) and the original VU fragment library (*N* = 1400, purple) in terms of the 3D metrics Fsp^3^, PBF and PMI (ΣNPR values). The distribution is displayed in violin plots and box plots. (B) Main scaffolds of the 3D fragment subset. Possible positions of substitution are highlighted in light blue. (C) Screening results of the 3D fragment subset. Fragments were screened at 10 μM (blue) and 30 μM (orange) in a [^3^H]mepyramine radioligand binding assay for human H_1_R. Data is normalized to full radioligand displacement. The normalized levels of residual bound radioligand are plotted on the vertical axis for each fragment shown on the horizontal axis. Fragment 29 (hit 1a) displaces [^3^H]mepyramine strongly (>85%) at both concentrations.

To enhance the 3D shape diversity of our in-house fragment library, we have been developing new synthetic chemistry protocols to obtain novel 3D scaffolds. As a result of these synthesis studies, we recently plated 80 diverse screening compounds that, on average and relative to our original fragment library,^28^ rank higher across the 3D metrics ‘fraction of sp^3^ carbon atoms’ (Fsp^3^),^22,29^ ‘Plane of Best Fit’ (PBF)^30^ and ‘Principal Moment of Inertia’ (PMI)^31^ (Fig. 1A). The plated compounds comprise various aliphatic (hetero)cyclic scaffolds including cyclobutane, cyclopropane and azabicyclic rings (Fig. 1B). While most compounds adhere to the Rule of Three (Ro3) criteria for fragment libraries,^32^ a few surrogates with slight deviations with respect to the molecular weight and rotatable bond count were included for specific synthetic reasons (*vide infra*). The 3D compounds were screened against H_1_R at 10 μM and 30 μM concentrations using a radioligand displacement assay. This led to the identification of cyclobutane hit 1a^17^ (Table 1), which displays strong displacement (>85%) of [^3^H]mepyramine at both concentrations (Fig. 1C). Notably, hit 1a demonstrates significant selectivity, as it was not identified as a hit in screenings against other GPCRs, including the histamine receptor subtypes H_3_R and H_4_R (data not shown).

**Table 1:** Initial hit validation and deconstruction. All p*K*_i_ values represent mean ± SD from at least *n* = 3

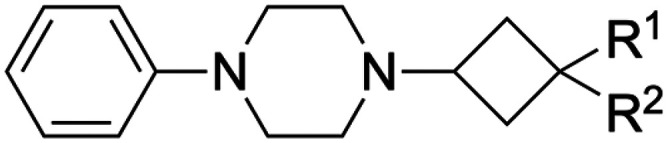								
Compound	R^1^	R^2^	MW	clog *P*	Isomer	p*K*_i_	LE	LLE
1a	–N_3_	–*n*Bu	313	4.8	*cis* a	7.5 ± 0.3	0.44	2.7
1b					*trans* a	6.6 ± 0.2	0.39	1.8
2a	–N_3_	–H	257	2.8	*cis*	6.6 ± 0.3	0.48	3.8
2b					*trans*	5.8 ± 0.2	0.42	3.0
3a	–NH_2_	–H	231	0.9	*cis*	5.1 ± 0.1	0.41	4.2
3b					*trans*	5.2 ± 0.2	0.42	4.3

a Relative stereochemical relationship between the phenylpiperazine and azide moiety.

Hit compound 1a does not fully adhere to the Ro3 criteria for a fragment.^32^ The *n*-butyl chain was installed to allow for a synthesis that avoids small organic azide building blocks that bear a high explosion risk.^17^ Similarly, the azide moiety, which is rarely found in fragment libraries, was initially introduced as a synthetic handle.^17^ By screening this surrogate compound, we aimed to maximize the chance of identifying the cyclobutane scaffold as a hit, with the intention to address undesired substituents during hit exploration. In the case of H_1_R, this particular screening compound was identified as a hit. Given the small number and limited diversity of the 3D compounds screened, it is difficult to compare the hit rate with other screening campaigns. However, it is worth noting that the original VU fragment library, which at the time contained 1010 structurally diverse screening compounds, achieved a hit rate of 3.6% when screened on H_1_R.^28^

Hit 1a was validated by establishing full dose–response curves. Interestingly, 1a has approximately an order of magnitude higher affinity (p*K*_i_) compared to its *trans* isomer 1b (Table 1). These results marked the beginning of a more elaborate hit exploration (Table 1), where our initial focus was the deconstruction of the hit by eliminating the *n*-butyl chain and azide moiety. Removal of the *n*-butyl chain resulted in a drop in affinity by approximately one log unit for both isomers (2a–b). Subsequent replacement of the azide group by an amine functionality caused an additional decrease in affinity by more than one log unit (3a–b). However, the ligand efficiency (LE)^33^ remained relatively unchanged throughout these modifications, while the lipophilic ligand efficiency (LLE)^34^ increased significantly as a result of the strong decrease in lipophilicity (*i.e.*, clog *P*), indicating the potential to grow to higher-affinity ligands from the bare fragments 3a–b.

Table 2 shows various carbon- (4–9), oxygen- (10–11) and nitrogen-based (12–13) substituents that were explored to establish the potential to grow fragments 3a–b and to explore the structure–activity relationships (SAR) around their scaffold. Owing to the stereochemical features of the synthetic strategy used (*vide infra*), many compounds could be tested as separate diastereomers. Specifically, similar quantities of separated *cis* and *trans* building blocks required for the nitrogen-based compounds were obtained, and thus both isomers could be obtained and tested for these analogs. For the carbon- and oxygen-based analogs, it was not always possible to obtain sufficient material of both isomers of the required building blocks, owing to the higher stereoselectivity in favor of the *cis* isomer in the key reaction step (d.r. ≈ 9 : 1, compared to d.r. ≈ 1 : 1 for the nitrogen-based building block) and a more challenging separation of the ensuing diastereomers. Therefore, the *trans* isomer could not always be obtained and tested for the compounds with carbon- and oxygen-based substituents. Carboxylic acid 8 was the only compound that was obtained as a mixture of two inseparable diastereomers.

**Table 2:** Hit exploration and structure–activity relationships of fragments 3a–b. All p*K*_i_ values represent mean ± SD from at least *n* = 3

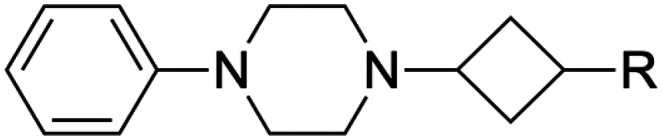							
Compound	R	MW	clog *P*	Isomer	p*K*_i_	LE	LLE
3a	–NH_2_	231	0.9	*cis*	5.1 ± 0.1	0.41	4.2
3b				*trans*	5.2 ± 0.2	0.42	4.3
4a	–CN	241	2.3	*cis*	5.0 ± 0.2	0.38	2.6
4b				*trans*	5.6 ± 0.2	0.42	3.2
5a	–CON(Me)_2_	287	1.8	*cis*	6.4 ± 0.3	0.42	4.5
5b				*trans*	—a	—	—
6a	–COOEt	288	2.7	*cis*	6.5 ± 0.3	0.42	3.7
6b				*trans*	—a	—	—
7a	–COOH	260	1.6	*cis*	<5.0	<0.35	<3.2
7b				*trans*	—a	—	—
8	–CH_2_COOH	274	2.6	*∼3 : 1 cis : trans*	5.4 ± 0.2	0.37	2.8
9a	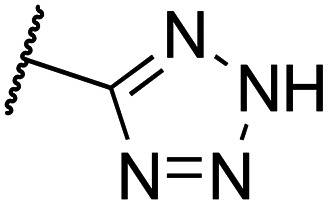	284	0.4	*cis*	6.3 ± 0.2	0.41	5.9
9b				*trans*	—a	—	—
10a	–OBn	322	3.7	*cis*	6.7 ± 0.3	0.38	3.0
10b				*trans*	7.0 ± 0.3	0.40	3.3
11a	–OH	232	1.7	*cis*	6.2 ± 0.3	0.50	4.6
11b				*trans*	6.3 ± 0.3	0.51	4.6
12a	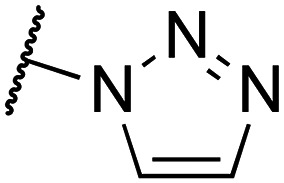	283	2.2	*cis*	5.5 ± 0.1	0.36	3.4
12b				*trans*	6.2 ± 0.3	0.40	4.0
13a	–NHCOMe	273	1.4	*cis*	6.3 ± 0.3	0.43	4.9
13b				*trans*	6.1 ± 0.3	0.42	4.7

a Product was not isolated or synthesis was not pursued.

Compared to their amine analogs 3a and 3b, nitriles 4a and 4b possess similar and slightly improved affinity, respectively, with a preference for the *trans* over the *cis* isomer. The LE remains relatively unchanged, while the LLE decreases significantly. Introduction of a dimethylamide moiety (5a) results in an increase in affinity by more than one log unit in comparison to amine 3a, accompanied by LE and LLE values that are on par with those of the amine. Its affinity is similar to ester 6a, but the ester possesses a comparatively low LLE and is of less interest because of concerns about hydrolytic instability. The carboxylic acid counterpart 7a is not tolerated as it has abolished affinity. The extended carboxylic acid 8 also binds only with low affinity, indicating that carboxylic acidic groups are unfavorable for affinity. However, ligand 9a, with a tetrazole as a well-known carboxylic acid isostere,^35^ has an affinity that is more than 15-fold improved when compared to fragment 3a. It possesses the highest LLE in the entire series, primarily because of the low clog *P* value. However, tetrazole 9a was not considered an ideal candidate for further hit exploration due to limitations in terms of growing vectors. Benzyloxy substituents (10a–b) show the highest affinities in the entire series, albeit at the expense of LE and, most notably, LLE. Removal of the benzyl group, giving alcohols 11a–b, provided ligands that are superior to amines 3a–b in terms of affinity, LE and LLE, making them attractive potential starting points for further hit exploration. Unsubstituted triazoles 12a–b were investigated because of their potential for rapid hit exploration through the conversion of azides 2a–b to triazoles using CuAAC chemistry. However, for the *cis* isomer 12a, affinity is only marginally higher compared to amine 3a, and comparatively low LE and LLE values are obtained. The *trans* isomer 12b performs better, with a p*K*_i_ value of 6.2 and efficiency metrics that more closely resemble those of amine 3b. Ultimately, amides 13a–b provided a favorable profile in terms of affinity, LE, and LLE. Additionally, the chemistry around the required building block was more accessible in comparison to amides 5a, ethers 10a–b and alcohols 11a–b. Therefore, amides 13a–b were selected for further exploration.

Optimization of amides 13a–b started with the introduction of various aliphatic rings of increasing size (Table 3). In this series (*i.e.*, 13c–h), the cyclopentyl substituent shows the highest affinity as well as the highest selectivity between the *cis* and *trans* isomer (13e and 13f). Comparing 13g–h and 13i–j, only minor changes were observed between aliphatic and aromatic six-membered rings. However, introduction of a methylene linker, affording benzylamides 13k–l, provided more than an order of magnitude increase in the p*K*_i_ value for the *cis* isomer 13k, with a significant difference between the *cis* and *trans* isomer 13k and 13l. Thus, the H_1_R affinity was improved 50-fold from methylamide 13a to benzylamide 13k, without substantial changes to the LE and LLE.

**Table 3:** Optimization of the amide substituent. All p*K*_i_ values represent mean ± SD from at least *n* = 3

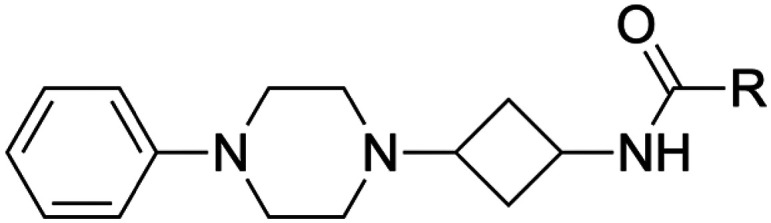							
Compound	R	MW	clog *P*	Isomer	p*K*_i_ H_1_R	LE	LLE
13a	–Me	273	1.4	*cis*	6.3 ± 0.3	0.43	4.9
13b				*trans*	6.1 ± 0.3	0.42	4.7
13c	–*c*Pr	299	2.1	*cis*	7.2 ± 0.2	0.45	5.1
13d				*trans*	7.0 ± 0.2	0.43	4.8
13e	–*c*Pent	327	2.9	*cis*	7.8 ± 0.2	0.45	4.9
13f				*trans*	7.1 ± 0.2	0.41	4.2
13g	–*c*Hex	341	3.4	*cis*	7.1 ± 0.1	0.39	3.8
13h				*trans*	6.9 ± 0.3	0.38	3.5
13i	–Ph	335	3.0	*cis*	6.6 ± 0.3	0.36	3.6
13j				*trans*	7.2 ± 0.3	0.39	4.2
13k	–CH_2_Ph	349	2.9	*cis*	8.0 ± 0.3	0.42	5.0
13l				*trans*	7.2 ± 0.3	0.38	4.3

Overall, replacement of the amine substituent in 3a with a benzylamide moiety afforded an approximate 800-fold improvement in affinity, accompanied by a significant increase in LLE (4.2 to 5.0) and a virtually unchanged LE (0.41 to 0.42). We therefore considered this side of the cyclobutane scaffold sufficiently optimized and shifted focus to the side that bears the phenylpiperazine moiety. Based on H_1_R ligand-based and structure-based knowledge, we hypothesized that the distance between the aromatic ring and the basic amine in this part of the molecule was likely suboptimal.^25^ Therefore, we essentially performed a fragment merging exercise to introduce a 4-(2-benzylphenoxy)piperidine—a moiety that we previously identified^36^ and explored^37,38^ using a H_1_R virtual fragment screening approach. This gave rise to a series of type II analogs with selected key substituents at the R^1^ position (Table 4). For this series, only the *cis* isomers were successfully synthesized because of instability of the *trans* isomer (*vide infra*). The stepwise introduction of the 4-(2-benzylphenoxy)piperidine moiety and its comparison to the type I analogs was performed with azide 2a as the reference compound. Introduction of the phenoxypiperidine moiety (14a) results in an increase in affinity by approximately one log unit and a slight increase in LE and LLE values when compared to type I counterpart 2a, indicating that the distance between the phenyl ring and the basic amine is indeed improved in the type II analogs. Introduction of the benzyl group on the R^2^-position (15a) provided the expected increase in affinity but also slightly lower LE and LLE compared to 14a. With the SAR established for the type I and type II substructures, the previously identified key substituents were reintroduced (*i.e.*, the amine group as in 3a and the benzylamide moiety as in 13f). As expected, reintroduction of the primary amine (16a) results in a slight decrease in affinity and a marked increase in LLE. Finally, reintroduction of the benzylamide (17a) restored the affinity to similar levels of azide 15a. This completed the transition from type I to type II analogs, with an overall approximate 6-fold gain in affinity and 1.4 units reduction in LLE compared to phenylpiperazine 13k.

**Table 4:** Optimization of the phenyl-amine distance by scaffold merging. All p*K*_i_ values represent mean ± SD from at least *n* = 3

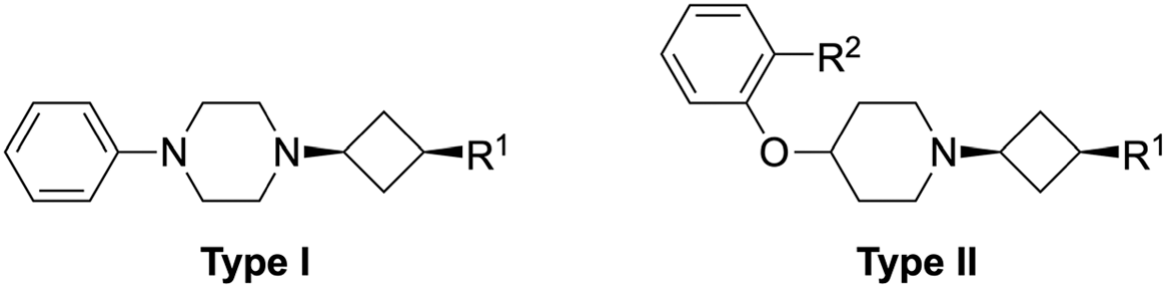								
Compound	Type	R^1^	R^2^	MW	clog *P*	p*K*_i_	LE	LLE
2a	I	–N_3_	—	257	2.8	6.6 ± 0.3	0.48	3.8
14a	II	–N_3_	–H	272	3.5	7.5 ± 0.1	0.52	4.0
15a	II	–N_3_	–Bn	362	5.0	8.8 ± 0.2	0.44	3.8
16a	II	–NH_2_	–Bn	336	3.1	8.4 ± 0.3	0.46	5.3
17a	II	–NHCOCH_2_Ph	–Bn	454	5.1	8.8 ± 0.3	0.35	3.6

**Fig. 2:**
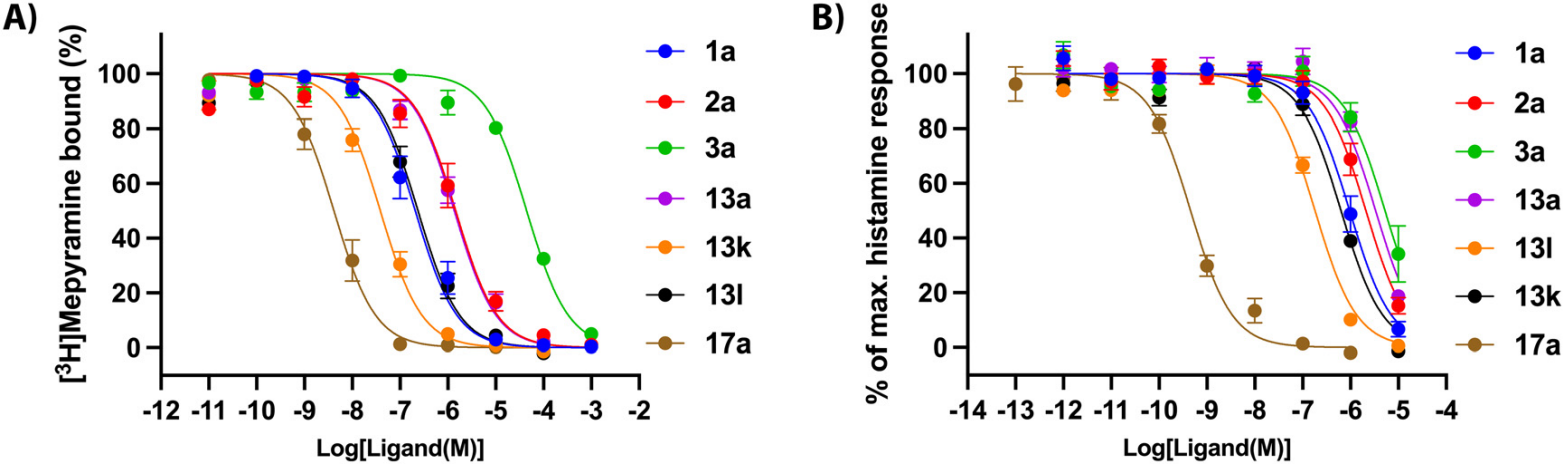
Concentration–response curves for key compounds 1a, 2a, 3a, 13a, 13k, 13l and 17a. (A) Increasing concentrations of ligands inhibit [^3^H]mepyramine binding to the human H_1_R expressed transiently in HEK 293T cells. Curves represent normalized data pooled from multiple experiments (*n* ≥ 3) and the error bars represent SEMs. (B) Antagonism against histamine-induced activity of the human H_1_R stably expressed in CHO cells in a NFAT-luciferase reporter gene assay. Curves represent normalized data pooled from multiple experiments (*n* ≥ 3) and the error bars represent SEMs.

Fig. 2A shows the dose–response curves for H_1_R binding of key compounds throughout the different SAR cycles (1a, 2a, 3a, 13a, 13k, 13l and 17a). These ligands were also characterized in a functional assay (Fig. 2B). In keeping with expectations, the key compounds inhibited histamine activity, with higher-affinity compounds generally also possessing higher antagonistic potency. Ligand 17a demonstrated a functional potency (p*K*_b_) of 10.1.

**Fig. 3:**
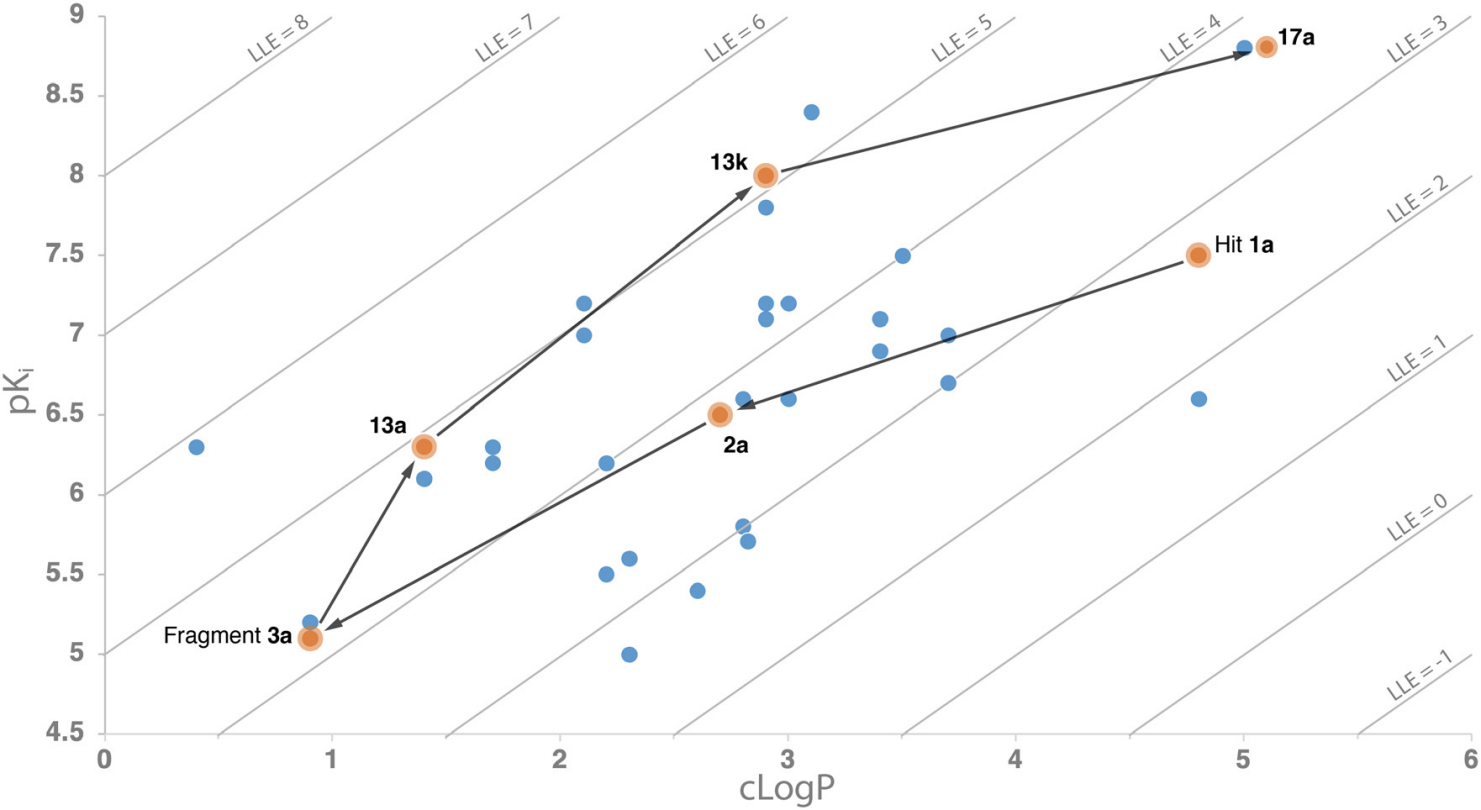
Hit exploration route in terms of clog *P* (horizontal axis), affinity (p*K*_i_; vertical axis) and LLE (p*K*_i_ – clog *P*; diagonal lines).

In all, hit 1a was first deconstructed by elimination of undesired features that were present for safety (–*n*Bu) or synthetic versatility (–N_3_) reasons, providing fragment 3a. This fragment has a relatively low affinity, but a similar LE and significantly improved LLE (Fig. 3). Subsequently, various substituents and linkers were explored, providing amide 13a as the starting point for a next optimization cycle. This gave benzylamide 13f, which possesses a 50-fold higher affinity and similar LLE when compared to 13a. Albeit at the expense of LLE, affinity could be further improved to give ligand 17a, which has a binding affinity of 8.8. Thus, the undesirable features that were present in the original hit could effectively be replaced while retaining the cyclobutane scaffold. Interestingly, significant differences in terms of affinity were observed for the *cis* and *trans* isomers throughout the series. This selectivity can be considered an advantage of using 3D fragments, although it requires strategies to obtain isomerically pure analogs. In this study, the symmetrical nature of the 1,3-disubstituted cyclobutane scaffold prevented the possibility of enantiomers and thereby reduced the (synthetic) complexity. In many cases, both diastereomers of the tested compounds could conveniently be synthesized from diastereomerically pure intermediate building blocks, facilitating an efficient hit exploration.

### 2.2. Computational modeling

**Fig. 4:**
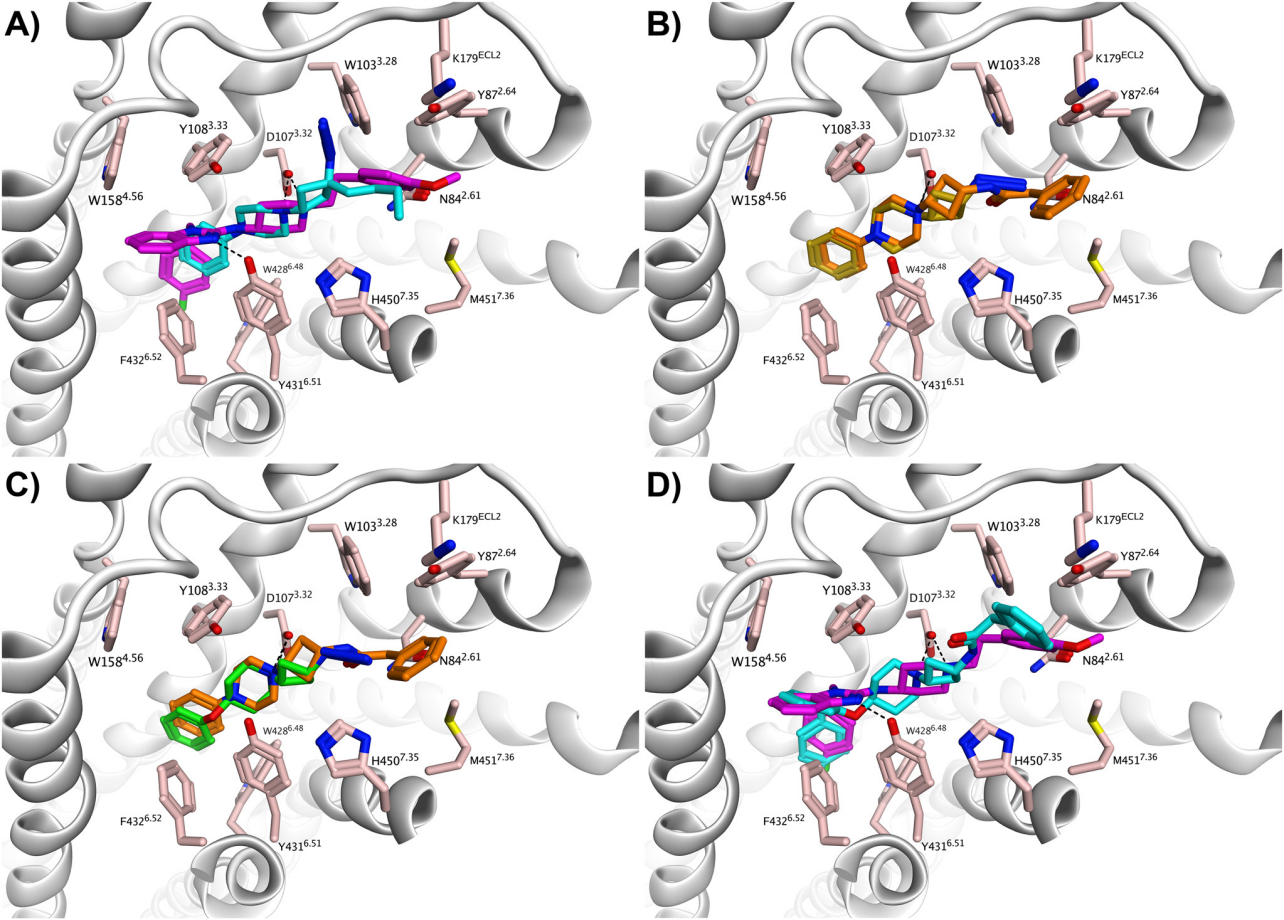
Compounds docked in the cryo-EM structure of the histamine H_1_ receptor in complex with astemizole (PDB: 8X5Y) using MOE.^27^ (A) Compound 1a (cyan carbon atoms) in overlay with astemizole (purple carbon atoms). (B) An overlay of 2a (gold carbon atoms) and 13k (orange carbon atoms). (C) An overlay of 13k (orange carbon atoms) and 14a (green carbon atoms). (D) An overlay of 17a (cyan carbon atoms) and astemizole (purple carbon atoms). Binding site residues are colored with salmon-colored carbon atoms. Oxygen, nitrogen and fluorine atoms are colored red, blue and green, respectively. Hydrogen bond interactions and ionic interactions are shown as dashed lines.

The cryo-EM structure of the H_1_R in complex with antagonist astemizole (Fig. 4A, purple carbon atoms; PDB: 8X5Y) was used for molecular modeling studies.^27^ In this structure, astemizole binds to the H_1_R by forming a salt-bridge between its basic amine and Asp107^3.32^, a hallmark binding motif that is also found in other H_1_R structures. The benzimidazole moiety of astemizole extends into the upper aromatic region and forms a hydrogen bond interaction with Tyr431^6.51^, while the fluorobenzene ring extends into the lower aromatic region of the main binding pocket, making hydrophobic interactions with Trp428^6.48^ and Phe432^6.52^. The methoxyphenyl moiety of astemizole was shown to occupy the secondary binding pocket, defined by residues of ECL2, TM2, TM3 and TM7, where it makes hydrophobic contacts with amino acids Tyr87^2.64^, Trp103^3.28^ and Met451^7.36^.^27^

Compounds 1–17 were docked into this astemizole-bound structure, hypothesizing that their scaffold would also occupy the secondary binding pocket. We deemed a binding pose relevant if the hallmark interaction between the basic amine of the ligands and Asp107^3.32^ was present, as well as a hydrophobic interaction with Trp428^6.48^ and/or Phe432^6.52^ (see SI for more details). In the obtained docking pose for hit 1a (Fig. 4A, cyan carbon atoms), the cyclobutane scaffold extends into the secondary binding pocket while the basic amine interacts with Asp107^3.32^. The phenyl ring of 1a points towards the lower aromatic region. The overlay with astemizole supports our hypothesis about the suboptimal distance between the aromatic ring and the basic amine in the type I analogs, as the lower aromatic region is not optimally occupied by the phenyl ring of hit fragment 1a.

Varying the substituents on the cyclobutane ring (Tables 2 and 3, compounds 2–13l) yielded binding modes similar to hit 1a. In Fig. 4B, the structures of 2a (gold carbon atoms) and 13k (orange carbon atoms) are presented to illustrate this. Docking of compound 14a (Table 4) confirms that the aromatic ring of the type II analogs reaches deeper into the lower aromatic region owing to the introduction of the ether linker, which is demonstrated in Fig. 4C by the overlay of type I analog 13k and type II analog 14a.

Finally, completion of the type I to type II transition through the merging of ligand 13k and the 4-(2-benzylphenoxy)piperidine moiety provided the most potent compound in the series (17a, Table 4). Its binding mode is presented in Fig. 4D (cyan carbon atoms) in overlay with astemizole (purple carbon atoms). The phenyl ring of the phenoxy group in 17a extends into the upper aromatic region while the benzyl group probes deep into the lower aromatic region. This is in line with the prediction of Kuhne *et al.* regarding the binding mode of this structural element.^37^ As hypothesized, the benzylamide moiety of 17a binds in the secondary binding pocket, similar to the methoxyphenyl group of astemizole. These modeling studies give further rationale to the efficient hit exploration.

Some limitations are associated with our modeling results. Although in some cases selectivity was observed between the *cis*- and *trans*-substituted cyclobutane ligands (*e.g.*, 13k*vs.*13l, Table 3), no clear trend emerged and based on the current modeling studies, a definitive explanation regarding differences in affinity between stereoisomers could not be established. The flexibility of ECL2, which partially forms the secondary binding pocket, contributes to the complexity of this analysis. Nevertheless, the experimental affinity data indicate that the cyclobutane ring plays an important role in positioning the substituent to engage in additional interactions within this pocket. An additional complicating factor is that most molecular docking algorithms struggle to sample interconversions between different aliphatic ring conformations during the docking simulation, which can hinder identification of the correct binding mode. When compared to aromatic fragments, this represents a potential disadvantage of (semi)flexible 3D fragments, at least in the context of computational studies.

### 2.3. Synthesis

**Scheme 1:**
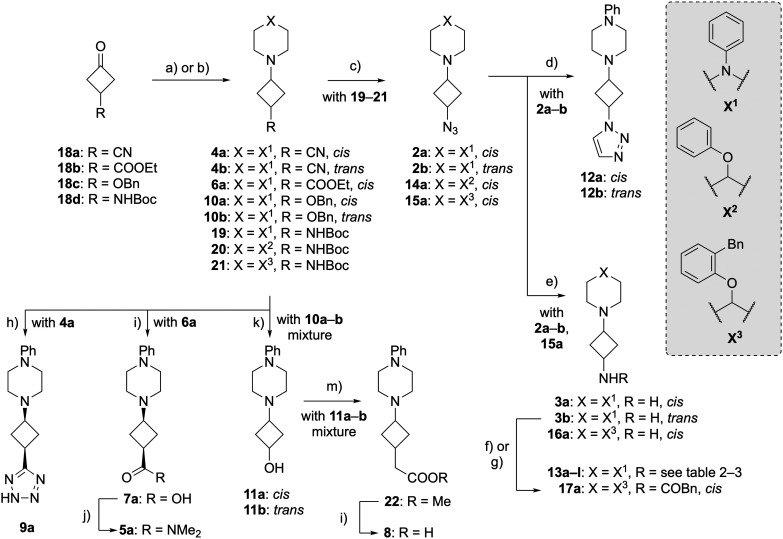
Synthesis towards the H_1_R ligands: (a) for 19–21; corresponding amine, NaBH(2-ethylhexanoate)_3_, 2-ethylhexanoate, THF, rt, 3–22 h, 73–87%; (b) for 4a–b, 6a and 10a–b; corresponding amine, NaBH(OAc)_3_, AcOH, THF, rt, 3–20 h, 66–86%; (c) [i] HCl, dioxane, rt, 16–36 h; [ii] ImSO_2_N_3_·H_2_SO_4_, K_2_CO_3_, CuSO_4_·5H_2_O, MeOH, rt, 16 h–3 d, 31–50% over two steps; (d) vinyl acetate, THF, 130 °C (μW), 36 h, 40–76%; (e) Pd/C, H_2_, MeOH, rt, on, 90–99%; (f) for 13a–b; AcCl, CH_2_Cl_2_, 0 °C → rt, 2 h, 49–64%; (g) for 13c–l and 17a; corresponding carboxylic acid, HATU, i-Pr_2_NEt, DMF, rt, on, 19–76%; (h) NaN_3_, NH_4_Cl, DMF, 110 °C, 40 h, 29%; (i) KOH, H_2_O, EtOH, 90 °C, 2–3 h, 22–52%; (j) Me_2_NH·HCl, HATU, i-Pr_2_NEt, DMF, rt, 20 h, 63%; (k) Pd/C, Pd(OH)_2_/C, H_2_, HCl, MeOH, rt, 20 h, 39%; (m) [i] (COCl)_2_, DMSO, Et_3_N, CH_2_Cl_2_, −78 °C → rt, 21 h; [ii] methyl 2-(diethoxyphosporyl)acetate, NaH, THF, 0 °C → rt, 4 h; [iii] Pd/C, Pd(OH)_2_/C, H_2_, HCl, MeOH, rt, 16% over three steps.

The synthesis commenced with a reductive amination of cyclobutanones 18a–d with the corresponding piperazine or piperidine, providing amines 4a–b, 6a, 10a–b and 19–21 (Scheme 1). Using NaBH(OAc)_3_ as the reducing agent, reductive aminations of the carbon- and oxygen-substituted cyclobutanes 18a–c gave primarily the *cis* isomer, with an approximate d.r. of 9 : 1 as inferred from LCMS analysis of reaction mixtures. This is in alignment with detailed studies on BH_4_^−^-based reductions of 3-benzoxy and 3-phenyl substituted cyclobutanones.^39^ However, reductive aminations with the NHBoc-substituted cyclobutanone 18d gave primarily the *trans* isomer in an approximate 1 : 3 *cis* : *trans* ratio. Although this change in stereoselectivity was unexpected, literature precedent supports this observation.^40^ In an attempt to steer the stereoselectivity away from the *trans* isomer, the sterically hindered reducing agent NaBH(2-ethylhexanoate)_3_ was used,^41^ which gave an improved ratio of approximately 1 : 1.

Separation of the diastereomers by normal phase column chromatography proved to be possible for nitriles 4a–b, ester 6a (6b was not obtained in sufficient quantity/purity), benzyl ethers 10a–b, and alcohols 11a–b, but could not be readily achieved for Boc-protected amines 19–21. Fortunately, following amine deprotection and subsequent copper-catalyzed diazo transfer,^42^ the diastereomers of the resulting azides 2, 14 and 15 were separable by column chromatography. However, only for piperazine 2, both isomers could be obtained, as the *trans* isomers of phenoxypiperidines 14 and 15 appeared to be unstable. For these two compounds, significant formation of an unknown impurity was observed after separation of the isomers by column chromatography and concentration of the relevant fractions. Despite multiple efforts to identify this impurity, we were unable to unequivocally determine the identity and cause of the impurity. Microwave-assisted cyclization with vinyl acetate of azides 2a–b gave triazoles 12a–b in moderate yield.^43^ Hydrogenolysis of azides 2a–b and 15a gave amines 3a–b and 16a, effecting the formal deprotection and diastereomeric separation of Boc-amines 19 and 21. These amines were reacted with AcCl to provide 13a and 13b, or with the corresponding carboxylic acids in a HATU-mediated amide coupling to provide amides 13c–l and 17a.

Ring closure of nitrile 4a with NaN_3_ provided tetrazole 9a. Ester 6a was hydrolyzed to provide carboxylic acid 7a, after which a HATU-mediated amide coupling provided dimethyl amide 5a. Pd-catalyzed hydrogenolysis of benzyl ether 10 (a mixture of *cis* and *trans* isomers), followed by chromatographic separation of the isomers provided alcohol 11a and 11b in a combined yield of 39%. Alcohol 11 (a mixture of *cis* and *trans* isomers) was subjected to a Swern oxidation, followed by a Wittig–Horner reaction and hydrogenation of the alkene to provide methyl ester 22. Following a failed attempt to isolate the presumably unstable^44,45^ 3-amino-ketone that resulted from the Swern oxidation, these three steps were combined into one procedure without isolation of the intermediates. Separation of the diastereomers at this stage proved to be challenging, and subsequent ester hydrolysis therefore provided carboxylic acid 8 as a mixture of diastereomers in a 3 : 1 *cis* : *trans* ratio.

The relative stereochemistry of all final compounds was determined at key stages in the synthetic pathway using 1D or 2D NOESY NMR experiments. When key proton signals showed significant overlap, GEMSTONE-NOESY experiments were used.^46^ For the *cis* isomers, we relied on direct correlations between the two CH cyclobutane protons, or, when such correlations could not be accurately measured or observed, on indirect correlations *via* the equatorial CH_2_ protons and the absence of strong correlations with the axial CH_2_ protons. For the *trans* isomers, direct CH–CH correlations were absent, while the axial and equatorial CH_2_ protons showed correlations with different CH protons, respectively.

## 3. Conclusion

Following our efforts to develop novel chemistries around 3D scaffolds, a selection of the resulting fragments and analogs was screened for binding affinity to the histamine H_1_ receptor. Despite the small library size of only 80 compounds, cyclobutane-based hit 1a (p*K*_i_ = 7.5) was identified. The first key step in the hit exploration was the removal of undesirable features (*i.e.*, the aliphatic chain and azide group), providing the bare fragment 3a with p*K*_i_ = 5.2. In total, fewer than 40 compounds were required to optimize fragment 3a towards high-affinity antagonist 17a (VUF26691, p*K*_i_ = 8.8), partially by benefitting from a fragment merging approach and by using structure- and ligand-based information. Overall, these results indicate that 3D fragments show potential in the identification of novel biologically active molecules. The success of 3D fragments is obviously target-dependent and the H_1_R can be considered a very druggable GPCR. Nevertheless, the fact that the chemical modality of interest—in this case the cyclobutane core—was maintained throughout the hit exploration supports our proposition that the fragment-based drug discovery approach is an effective method to incorporate novel chemistries into biologically active compounds.

## Author contributions

Tom Dekker: conceptualization, formal analysis, investigation, methodology, visualization, writing – original draft; Oscar P. J. van Linden: formal analysis, investigation, methodology, visualization, writing – original draft; Herman D. Lim: formal analysis, investigation, methodology, visualization; Mabel E. Dekker: formal analysis, investigation; Henry F. Vischer: methodology; Rob Leurs: methodology; Tiffany van der Meer: formal analysis, investigation; Maurice C. M. L. Buzink: formal analysis, investigation; David J. Hamilton: investigation; Barbara Zarzycka: investigation; Elwin Janssen: investigation, methodology; Maikel Wijtmans: conceptualization, funding acquisition, supervision, writing – review & editing; Iwan J. P. de Esch: conceptualization, funding acquisition, supervision, writing – review & editing.

## Conflicts of interest

There are no conflicts of interest to declare.

## Supplementary Material

MD-017-D5MD01081K-s001

## Data Availability

The data supporting this article have been included as part of the supplementary information (SI). Supplementary information: experimental details and NMR spectra. See DOI: https://doi.org/10.1039/d5md01081k.
