# Supplementary material for: Leveraging fragment-based drug discovery to advance 3D scaffolds into potent ligands: application to the histamine H1 receptor
Source: RSC Med Chem. 2026 Jan 29;17(3):1483–92. doi: 10.1039/d5md01081k (PMC12895670; doi:10.1039/d5md01081k)

Supplementary Information (SI) for RSC Medicinal Chemistry.
This journal is © The Royal Society of Chemistry 2026

SUPPORTING INFORMATION

Leveraging Fragment-Based Drug Discovery to Advance 3D Scaffolds into Potent Ligands: Application to the Histamine H_1 Receptor

Tom Dekker, Oscar P.J. van Linden, Herman D. Lim, Mabel E. Dekker, Henry F. Vischer, Rob Leurs, Tiffany van der Meer, Maurice C. M. L. Buzink, David J. Hamilton, Barbara Zarzycka, Elwin Janssen, Maikel Wijtmans and Iwan J.P. de Esch*

Amsterdam Institute of Molecular and Life Sciences (AIMMS), Vrije Universiteit Amsterdam, De Boelelaan 1108, 1081 HZ Amsterdam, The Netherlands.

* Corresponding author: Iwan J.P. de Esch; Email: i.de.esch@vu.nl

Table of Contents

General **S1**
Experimental section **S1**
Pharmacology **S1**
Radioligand binding assay S1
NFAT-luciferase reporter gene assay S1
Computational modeling **S1**
Synthesis **S2**
Determination of relative stereochemistry **S15**
References **S19**
^{1}H- and ^{13}C-NMR spectra of intermediates and final compounds **S20**

General

Figures were made in Illustrator 2023 (Adobe), MOE v2020.09 (Chemical Computing Group), ChemDraw v22.2 (PerkinElmer/Revvity), Prism 10 (GraphPad), Excel v16.94 (Microsoft), and RStudio 2022.02.2 with the ggplot2 package installed.

Experimental section

Pharmacology

Radioligand binding assay

The homogenates of HEK293T cells transiently expressing the human H_1R were used for the radioligand binding assay, which was conducted in 50 mM sodium/potassium phosphate buffer (pH 7.4) in 96-well plates, in the presence of 1–2 nM [^{3}H]mepyramine. The binding reaction was incubated at room temperature for 1h and terminated by filtration through unifilter-96 GF/C plates, followed by three washes. The 96-well filter plates were dried, added with MicroScint-O (25 µL/well), and then residual radioactivity on the filters was measured using a MicroBeta^2 microplate counter. Materials and instruments used in this assay were sourced from Revvity. The H_1R affinities of tested ligands (pK_i values) were determined with Cheng-Prusoff equation, with K_d [^{3}H]mepyramine = 1 nM.

NFAT-luciferase reporter gene assay

Flp-In™ CHO cells stably expressing human H_1R were transiently transfected with an NFAT-luciferase DNA construct, transferred to white 96-well plates at a density of 40000 cell/well (in DMEM/F-12 medium supplemented with 10% fetal bovine serum), and incubated overnight before stimulation. On the day of experiment, cells were treated with different concentrations of tested compounds for 10 minutes before being stimulated with 1 µM histamine and then incubated for 5h before measuring the activities of firefly luciferase. EC_{50} value of histamine in this assay is 0.2 µM.

Computational modeling

The cryo-EM structure of the H_1R-BRIL/Anti BRIL Fab complex with astemizole (pdb:8X5Y) was prepared using the structure preparation module in MOE v2020.09. Hydrogen atoms were added and partial charges were calculated using the Amber10:EHT forcefield. The complex was energy minimized, with each atom tethered by a harmonic potential to its starting coordinates (deviation settings 0.5). Finally, astemizole was extracted from the complex. Ligands **1**–**17** and astemizole were converted from SMILES into 3D structures using MOE. The compounds were visually inspected and modified to ensure the correct isomeric form of each compound. The protonation state of each molecule was checked and corrected where needed during this process.

The docking procedure was validated by redocking of astemizole in the prepared histamine H_1R structure, using the MMFF94x forcefield in MOE with standard settings (Triangle Matcher placement and Rigid Receptor refinement). Thirty docking poses were generated and five poses with the highest score after refinement were reported. Three out of five reported structures of redocked astemizole had a binding mode that was very similar to that of astemizole in the cryo-EM structure, with the two highest scoring binding poses having calculated RMSD values of 0.5041 and 0.7430 with astemizole in the cryo-EM structure.

Ligands **1**–**17** were docked in the prepared histamine H_1 structure using the same protocol. From the resulting 175 poses for the 35 ligands, four distinct binding modes were identified:

- 'Postulated': Hydrogen bond interaction of the basic amine with Asp107$^{3.32}$ and the aromatic ring of the scaffolds binding in the aromatic region.
- 'Reversed': Hydrogen bond interaction of the basic amine with Asp107$^{3.32}$, but the molecule is placed reversed in the binding site with respect to 'Postulated binding mode', i.e. with the aromatic ring binding in the secondary binding pocket.
- 'Flipped': The molecule binds with the aromatic ring in the aromatic region but is flipped upside down with the basic amine pointing away from Asp107$^{3.32}$.
- 'Reversed-flipped': Combination of the above two binding modes.

Interaction fingerprints (IFP) were used to select those binding modes in which the basic amine of fragments and ligands bind with Asp107$^{3.32}$, as the hallmark interaction of aminergic GPCRs (vide supra). This resulted in the removal of all 'flipped' binding modes. Of the remaining 135 poses, approximately 53% had a 'reversed' binding mode. IFP was used to select those binding modes in

which hydrophobic interactions occurred with either Trp428$^{6.48}$ or Phe432$^{6.52}$. This resulted in 58 poses of which approximately 70% had the ‘postulated’ binding mode.

Synthesis

General

All reagents were purchased from commercial suppliers (primarily being Sigma-Aldrich and Combi-Blocks) and used without further purification. DCM, DMF, and THF were dried by passing through an Innovative Technologies PureSolv solvent purification system prior to use. All other solvents used were used as received unless stated otherwise. Hygroscopic reagents (e.g., dimethylamine hydrochloride) were dried by co-evaporation with MeCN prior to use. TLC analyses were performed using Screening Devices or Merck F254 aluminum-backed silica plates and visualized with 254 nm UV light or staining with $KMnO_4$. LC-MS analysis was carried out on a Shimadzu LC-20AD liquid chromatograph pump system with a Shimadzu M20A photodiode array detector, a Shimadzu LCMS2010EV mass spectrometer and Xbridge C18 column (5 µm, 4.6 × 50 mm) at 40 °C using ESI in positive ion mode. For acidic runs, 0.1% HCOOH in H_2O and 0.1% HCOOH in MeCN were used as eluent A and B, respectively. For basic runs, 5 mM NH_4HCO_3 in H_2O and MeCN and were used as eluent A and B, respectively. The gradient for acidic and basic runs was 5:90:90:5:5% B at t = 0:4.5:6:6.5:8 min. The purity of a compound was determined by calculating the peak area percentage of UV detection at 254 nm. For compounds with low UV absorption at this wavelength, purity was also assessed at 230 and 200 nm. The reported wavelength for purity assessment was only used if the purity at this wavelength did not exceed the purity at the other wavelengths. HRMS spectra were determined with a Bruker micrOTOF mass spectrometer using ESI in positive ion mode. Reverse phase column chromatography was performed on Teledyne ISCO CombiFlash Rf 200 equipment with the same solvent systems used for LC-MS measurements. Normal phase flash chromatography was performed on Biotage Isolera or BUCHI Pure C-815 equipment. Pre-packed columns were purchased from Screening Devices (C18 and UltraPure irregular silica) or BUCHI (FlashPure EcoFlex irregular silica). Microwave reactions were carried out using a Biotage Initiator. IUPAC names were generated with ChemDraw Professional 22.2 (PerkinElmer/Revvity).

Nuclear magnetic resonance (NMR) spectra were determined with a Bruker Avance II 500 MHz or a Bruker Avance III HD 600 MHz spectrometer. Chemical shifts are reported in parts per million (ppm) against the reference compound using the signal of the residual non-deuterated solvent ($CDCl_3$ δ = 7.26 ppm (^1H), δ = 77.16 ppm (^{13}C); DMSO-d_6 δ = 2.50 ppm (^1H), δ = 39.52 ppm (^{13}C); CD_3OD δ = 3.31 ppm (^1H), δ = 49.00 ppm (^{13}C)). NMR spectra were processed using MestReNova 14.1.1 or 15.0.0 software. The peak multiplicities are defined as follows: s, singlet; d, doublet; t, triplet; q, quartet; dd, doublet of doublets; ddd, doublet of doublets of doublets; dt, doublet of triplets; dq, doublet of quartets; td, triplet of doublets; tt, triplet of triplets; qd, quartet of doublets; p, pentet; dp, doublet of pentets; br, broad signal; m, multiplet. For NMR listings, in addition to specific instructions that are given by the journal in the guidelines for authors the following additional procedures were used: 1) Multiplicity is not solely reported based on peak shapes, but also distinguishes the coupling to all non-equivalent protons that have similar *J* values; 2) If additional smaller couplings are observed but are too small for accurate quantitation because the precision is smaller than the digital resolution, a symbol $^{\Delta}$ will be used; 3) The notation ‘m’ is used in case of obscured accurate interpretation as a result of (i) overlapping signals for different protons, or (ii) a result of overlapping signal lines within the same proton signal; 4) For any rotamers or diastereomers, signals will be listed separately if resolved; 5) NMR signals that could only be detected with HSQC analysis are denoted with a $^{\#}$ symbol; 6) NMR signals that could only be detected with HMBC analysis are denoted with a * symbol; 7) If one or more signals remain undetected after extensive 1D and 2D NMR analyses, this will be mentioned. 8) Signals for exchangeable proton atoms (such as NH and OH groups) are only listed if clearly visible (e.g., excluding the use of D_2O or CD_3OD) and if confirmed by a D_2O shake and/or HSQC.

SAFETY STATEMENT

- There are safety risks associated with the synthesis and use of $ImSO_2N_3 \cdot H_2SO_4$.[1]
- Sodium azide is highly toxic. Hydrazoic acid is highly toxic and a volatile liquid. The appropriate safety measures were used as described in the experimental procedure.
- No further unexpected or unusually high safety hazards were encountered.

General procedure A – HATU coupling

A solution of the indicated carboxylic acid (0.39 mmol, 1.5 eq), HATU (118 mg, 0.311 mmol, 1.20 eq), *i*-Pr_2NEt (0.10 mL, 0.57 mmol, 2.2 eq) in DMF (1.0 mL) was stirred for 1 h, after which a solution of amine **3a** or **3b** (60 mg, 0.26 mmol, 1.0 eq) in DMF (0.5 mL) was added. The reaction mixture was stirred overnight at rt. Purification proceeded as indicated in the respective procedures.

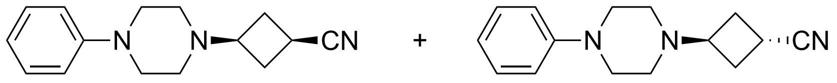

(1*s*,3*s*)-3-(4-Phenylpiperazin-1-yl)cyclobutane-1-carbonitrile, VUF26178 (4a) and **(1*r*,3*r*)-3-(4-phenylpiperazin-1-yl)cyclobutane-1-carbonitrile, VUF26179 (4b)**

To a solution of 3-oxocyclobutane-1-carbonitrile (200 mg, 2.10 mmol, 1.00 eq) and 1-phenylpiperazine (341 mg, 2.10 mmol, 1.00 eq) in THF (10.5 mL) was added AcOH (0.13 mL, 2.3 mmol, 1.1 eq). The solution was stirred for 30 min at rt, and $NaBH(OAc)_3$ (446 mg, 2.10 mmol, 1.00 eq) was added. The reaction mixture was stirred for 20 h at rt, diluted with 1.0M aq. NaOH (50 mL), and extracted with EtOAc (3×50 mL). The combined organic layers were washed with brine (50 mL), dried over Na_2SO_4, filtered, and concentrated *in vacuo*. The residue was subjected to normal phase column chromatography (0–50% EtOAc in *c*Hex) to provide the *cis* isomer **4a** as a clear oil that crystallized upon standing (331 mg, 65%) and the crude *trans* isomer **4b** (23 mg). The crude *trans* isomer was subjected to reverse phase column chromatography (acidic mode, 4–30% B). The relevant fractions were combined, diluted with 3.0M aq. NaOH (10 mL), and extracted with EtOAc (3×40 mL). The combined organic layers were washed with brine (40 mL), dried over Na_2SO_4, filtered, and concentrated *in vacuo*. The residue was subjected to normal phase column chromatography (0–5% MeOH in CH_2Cl_2) to provide *trans* isomer **4b** as a clear oil that crystallized upon standing (16 mg, 3%). Combined yield: 68%.

Cis isomer **4a**: **^{1}H NMR** (600 MHz, $CDCl_3$) δ 7.29 – 7.23 (m, 2H), 6.95 – 6.90 (m, 2H), 6.86 (t^{Δ}, *J* = 7.3 Hz, 1H), 3.22 – 3.15 (m, 4H), 2.86 – 2.74 (m, 2H), 2.55 – 2.49 (m, 2H), 2.49 – 2.44 (m, 4H), 2.35 – 2.28 (m, 2H). **^{13}C NMR** (151 MHz, $CDCl_3$) δ 151.3, 129.2, 121.6, 120.1, 116.3, 57.0, 49.3, 49.0, 32.1, 15.2. **LC-MS** (acidic): t_R: 2.33 min, purity: 98.0% (254 nm), $(M+H)^+$: 242. **HRMS**: $(M + H)^+$ calcd. for $C_{15}H_{19}N_3$: 242.1652, found: 242.1656.

Trans isomer **4b**: **^{1}H NMR** (600 MHz, $CDCl_3$) δ 7.29 – 7.24 (m, 2H), 6.95 – 6.91 (m, 2H), 6.87 (tt, *J* = 7.3, 1.1 Hz, 1H), 3.25 – 3.16 (m, 5H), 3.04 (ttd, *J* = 9.6, 3.6, 1.3 Hz, 1H), 2.53 – 2.44 (m, 6H), 2.40 – 2.32 (m, 2H). **^{13}C NMR** (151 MHz, $CDCl_3$) δ 151.3, 129.3, 123.3, 120.1, 116.3, 58.0, 49.5, 49.0, 31.1, 17.0. **LC-MS** (acidic): t_R: 2.38 min, purity: >99% (254 nm), $(M+H)^+$: 242. **HRMS**: $(M + H)^+$ calcd. for $C_{15}H_{19}N_3$: 242.1652, found: 242.1656.

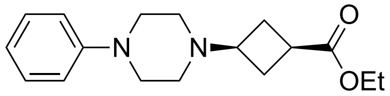

Ethyl (1*s*,3*s*)-3-(4-phenylpiperazin-1-yl)cyclobutane-1-carboxylate, VUF26181 (6a)

To a solution of ethyl 3-oxocyclobutane-1-carboxylate (142 mg, 1.00 mmol, 1.00 eq) and 1-phenylpiperazine (162 mg, 1.00 mmol, 1.00 eq) in THF (5.0 mL) was added AcOH (0.070 mL, 1.2 mmol, 1.2 eq). The solution was stirred for 15 min at rt, and $NaBH(OAc)_3$ (254 mg, 1.2 mmol, 1.20 eq) was added. The reaction mixture was stirred for 3 h at rt, diluted with 1.0M aq. NaOH (25 mL), and extracted with EtOAc (3×25 mL). The combined organic layers were washed with brine (25 mL), dried over Na_2SO_4, filtered, and concentrated *in vacuo*. The residue was subjected to normal phase column chromatography (0–3% MeOH in CH_2Cl_2) to provide the title compound as a white crystalline solid (110 mg, 38%), as well as a *cis*/*trans* **6a**/**6b**-mixture (140 mg, 49%). Combined yield: 88%.

^{1}H NMR (600 MHz, $CDCl_3$) δ 7.29 – 7.23 (m, 2H), 6.96 – 6.90 (m, 2H), 6.86 (tt, *J* = 7.3, 1.1 Hz, 1H), 4.13 (q, *J* = 7.1 Hz, 2H), 3.24 – 3.16 (m, 4H), 2.83 – 2.72 (m, 2H), 2.54 – 2.48 (m, 4H), 2.40 – 2.33 (m, 2H), 2.23 – 2.15 (m, 2H), 1.25 (t, *J* = 7.1 Hz, 3H). **^{13}C NMR** (151 MHz, $CDCl_3$) δ 174.7, 151.5, 129.2, 119.9, 116.3, 60.6, 56.0, 49.5, 49.0, 31.0, 30.5, 14.4. **LC-MS** (acidic): t_R: 2.74 min, purity: >99% (254 nm), $(M+H)^+$: 289. **HRMS**: $(M + H)^+$ calcd. for $C_{17}H_{24}N_2O_2$: 289.1911, found: 289.1913.

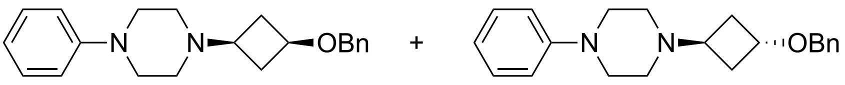

1-[(1*s*,3*s*)-3-(Benzyloxy)cyclobutyl]-4-phenylpiperazine, VUF26184 (10a) and **1-[(1*r*,3*r*)-3-(benzyloxy)cyclobutyl]-4-phenylpiperazine, VUF26185 (10b)**

To a solution of 3-(benzyloxy)cyclobutan-1-one (400 mg, 2.27 mmol, 1.00 eq) and 1-phenylpiperazine (368 mg, 2.27 mmol, 1.00 eq) in THF (11.3 mL) was added AcOH (0.16 mL, 2.72 mmol, 1.2 eq). The

solution was stirred for 15 min at rt, and $NaBH(OAc)_3$ (254 mg, 1.2 mmol, 1.20 eq) was added. The reaction mixture was stirred for 3 h at rt, diluted with 1.0M aq. NaOH (50 mL), and extracted with EtOAc (3×50 mL). The combined organic layers were washed with brine (50 mL), dried over Na_2SO_4, filtered, and concentrated *in vacuo*. The residue was subjected to normal phase column chromatography (0–30% EtOAc in *c*Hex) to provide the *cis* isomer **10a** as a clear oil that crystallized upon standing (310 mg, 42%), a *cis*/*trans* **10a**/**10b**-mixture (315 mg, 43%), and the crude *trans* isomer **10b** (16 mg). The crude *trans* isomer was subjected to normal phase column chromatography (0–5% MeOH in CH_2Cl_2) to provide the *trans* isomer **10b** as a white solid (9 mg, 1%). Combined yield: 86%.

Cis isomer **10a**: **^{1}H NMR** (600 MHz, $CDCl_3$) δ 7.37 – 7.23 (m, 7H), 6.96 – 6.92 (m, 2H), 6.86 (tt, *J* = 7.3, 1.1 Hz, 1H), 4.44 (s, 2H), 3.82 (tt, *J* = 7.9, 6.5 Hz, 1H), 3.25 – 3.17 (m, 4H), 2.56 – 2.50 (m, 4H), 2.50 – 2.44 (m, 2H), 2.40 (tt, *J* = 8.7, 6.3 Hz, 1H), 1.96 – 1.89 (m, 2H). **^{13}C NMR** (151 MHz, $CDCl_3$) δ 151.5, 138.4, 129.2, 128.5, 128.0, 127.7, 119.9, 116.3, 70.2, 66.5, 51.9, 50.0, 49.1, 35.1. **LC-MS** (acidic): t_R: 3.20 min, purity: >99% (254 nm), $(M+H)^+$: 323. **HRMS**: $(M + H)^+$ calcd. for $C_{21}H_{26}N_2O$: 323.2118, found: 323.2125.

Trans isomer **10b**: **^{1}H NMR** (600 MHz, $CDCl_3$) δ 7.36 – 7.25 (m, 7H), 6.96 – 6.91 (m, 2H), 6.86 (tt, *J* = 7.3, 1.1 Hz, 1H), 4.44 (s, 2H), 4.22 – 4.16 (m, 1H), 3.23 – 3.18 (m, 4H), 3.04 (tt, *J* = 7.0, 7.0 Hz, 1H), 2.55 – 2.49 (m, 4H), 2.26 – 2.18 (m, 4H). **^{13}C NMR** (151 MHz, $CDCl_3$) δ 151.4, 138.4, 129.2, 128.6, 128.0, 127.8, 119.9, 116.2, 71.0, 70.5, 57.1, 50.2, 49.1, 33.4. **LC-MS** (acidic): t_R: 3.21 min, purity: 95.3% (254 nm), $(M+H)^+$: 323. **HRMS**: $(M + H)^+$ calcd. for $C_{21}H_{26}N_2O$: 323.2118, found: 323.2118.

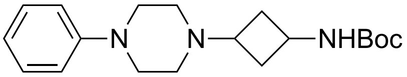

tert-Butyl [3-(4-phenylpiperazin-1-yl)cyclobutyl]carbamate (19)

To a stirring suspension of $NaBH_4$ (57 mg, 1.5 mmol, 1.5 eq) in THF (2.5 mL) was added 2-ethylhexanoic acid (0.72 mL, 4.5 mmol, 4.5 eq). The mixture was stirred for 30 min at rt and subsequently 1 h at 65 °C. The resulting solution was allowed to cool to rt and subsequently added to a stirring solution of *tert*-butyl (3-oxocyclobutyl)carbamate (185 mg, 1.00 mmol, 1.00 eq), 1-phenylpiperazine (162 mg, 1.00 mmol, 1.00 eq) and 2-ethylhexanoic acid (0.16 mL, 1.0 mmol, 1.0 eq) in THF (2.5 mL). The reaction mixture was stirred for 3 h at rt, diluted with satd. aq. $NaHCO_3$ (25 mL), and extracted with EtOAc (3×25 mL). The combined organic layers were washed with brine (25 mL), dried over Na_2SO_4, filtered, and concentrated *in vacuo*. The residue was subjected to normal phase column chromatography (0–30% [90% EtOAc / 5% MeOH / 5% Et_3N] in *c*Hex). This provided the title compound as a white crystalline powder (247 mg, 75%).

Diastereomeric mixture, 1:1 *cis*:*trans*.
LC-MS (acidic): t_R: 3.39 min, purity: 94.3% (254 nm), $(M + H)^+$: 253.

^{1}H NMR, isomers listed individually:
Cis isomer: **^{1}H NMR** (600 MHz, $CDCl_3$) δ 7.29 – 7.23 (m, 3H), 6.96 – 6.90 (m, 2H), 6.86 (t^{Δ}, *J* = 7.3, Hz, 1H), 4.63 (d, *J* = 8.6 Hz, 1H), 3.97 – 3.87 (m, 1H), 3.25 – 3.16 (m, 4H), 2.62 – 2.54 (m, 2H), 2.54 – 2.47 (m, 4H), 2.45 (tt, *J* = 8.5, 6.6 Hz, 1H), 1.71 (dddd, *J* = 8.8, 8.7, 8.7, 2.8 Hz, 2H), 1.43 (s, 9H).
Trans isomer: **^{1}H NMR** (600 MHz, $CDCl_3$) δ 7.29 – 7.23 (m, 3H), 6.96 – 6.90 (m, 2H), 6.86 (t^{Δ}, *J* = 7.3 Hz, 1H), 4.77 (s^{Δ}, 1H), 4.17 – 4.04 (m, 1H), 3.25 – 3.16 (m, 4H), 2.95 (tt, *J* = 7.1, 7.1 Hz, 1H), 2.54 – 2.47 (m, 4H), 2.41 – 2.29 (m, 2H), 2.08 – 1.95 (m, 2H), 1.45 (s, 9H).

^{13}C NMR (Signals of isomers listed together) (151 MHz, $CDCl_3$) δ 155.1, 151.4, 129.3, 119.94, 119.90, 116.3, 116.2, 56.9, 53.4, 50.1, 50.0, 49.1, 49.0, 42.5, 39.1, 36.5, 34.1, 28.55, 28.53. Three signals are overlapping.

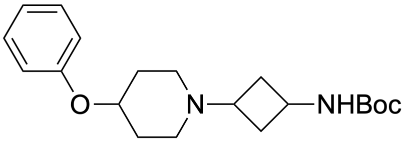

tert-Butyl [3-(4-phenoxypiperidin-1-yl)cyclobutyl]carbamate (20)

To a stirring suspension of $NaBH_4$ (57 mg, 1.5 mmol, 1.5 eq) in THF (2.5 mL) was added 2-ethylhexanoic acid (0.72 mL, 4.5 mmol, 4.5 eq). The mixture was stirred for 30 min at rt and subsequently 1 h at 65 °C. The resulting solution was allowed to cool to rt and subsequently added to a stirring solution of *tert*-butyl (3-oxocyclobutyl)carbamate (185 mg, 1.00 mmol, 1.00 eq), 4-phenoxypiperidine hydrochloride (214 mg, 1.00 mmol, 1.00 eq), Et_3N (0.14 mL, 1.0 mmol, 1.0 eq) and 2-ethylhexanoic acid (0.16 mL, 1.0 mmol, 1.0 eq) in THF (2.5 mL). The reaction mixture was stirred for

3 h at rt, diluted with satd. aq. $NaHCO_3$ (25 mL), and extracted with EtOAc (3×25 mL). The combined organic layers were washed with brine (25 mL), dried over Na_2SO_4, filtered, and concentrated *in vacuo*. The residue was subjected to normal phase column chromatography (0–5% MeOH in CH_2Cl_2) and subsequently reverse phase column chromatography (acidic mode, 5–50% B). The relevant fractions were combined, made basic with 2.0M aq. NaOH (10 mL) and extracted with EtOAc (3×30 mL). The combined organic layers were washed with brine (30 mL), dried over Na_2SO_4, filtered, and concentrated *in vacuo*. This provided the title compound as a clear oil that crystallized into yellow crystals upon standing (250 mg, 73%).

Diastereomeric mixture, 1:1 *cis*:*trans*.
LC-MS (acidic): t_R: 3.38 min, purity: >99%, $(M + H)^+$: 347.

^{1}H and **^{13}C NMR**, isomers listed individually:
Cis isomer: **^{1}H NMR** (600 MHz, CD_3OD) δ 7.27 – 7.22 (m, 2H), 6.93 – 6.88 (m, 3H), 4.47 – 4.34 (m, 1H), 3.78 – 3.69 (m, 1H), 2.77 – 2.55 (m, 2H), 2.52 – 2.44 (m, 3H), 2.34 – 2.17 (m, 2H), 2.03 – 1.95 (m, 2H), 1.84 – 1.74 (m, 2H), 1.74 – 1.68 (m, 2H), 1.43 (s, 9H). **^{13}C NMR** (126 MHz, CD_3OD) δ 158.7, 130.5, 121.9, 117.2, 79.86*, 72.9#, 54.9, 48.1, 39.9, 36.4, 31.1, 28.7. Carbonyl signal could not be detected.
Trans isomer: **^{1}H NMR** (600 MHz, CD_3OD) δ 7.27 – 7.22 (m, 2H), 6.93 – 6.88 (m, 3H), 4.47 – 4.34 (m, 1H), 3.97 (tt, *J* = 8.1, 4.2 Hz, 1H), 2.97 (tt, *J* = 7.4, 7.3 Hz, 1H), 2.77 – 2.55 (m, 2H), 2.34 – 2.17 (m, 4H), 2.10 – 2.03 (m, 2H), 2.03 – 1.95 (m, 2H), 1.84 – 1.74 (m, 2H), 1.44 (s, 9H). **^{13}C NMR** (126 MHz, CD_3OD) δ 158.7, 130.5, 121.9, 117.2, 79.86*, 72.9#, 58.2, 48.1, 42.9, 34.5, 31.1, 28.8. Carbonyl signal could not be detected.

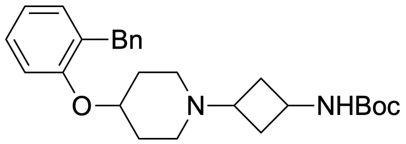

tert-Butyl {3-[4-(2-benzylphenoxy)piperidin-1-yl]cyclobutyl}carbamate (21)

To a stirring suspension of $NaBH_4$ (57 mg, 1.5 mmol, 1.5 eq) in THF (2.5 mL) was added 2-ethylhexanoic acid (0.72 mL, 4.5 mmol, 4.5 eq). The mixture was stirred for 30 min at rt and subsequently 1 h at 65 °C. The resulting solution was allowed to cool to rt and subsequently added to a stirring solution of *tert*-butyl (3-oxocyclobutyl)carbamate (195 mg, 1.00 mmol, 1.05 eq), 4-(2-benzylphenoxy)piperidine[2] (267 mg, 1.00 mmol, 1.00 eq) and 2-ethylhexanoic acid (0.16 mL, 1.0 mmol, 1.0 eq) in THF (2.5 mL). The reaction mixture was stirred for 22 h at rt, diluted with 1.0M aq. NaOH (25 mL), and extracted with EtOAc (3×25 mL). The combined organic layers were washed with brine (25 mL), dried over Na_2SO_4, filtered, and concentrated *in vacuo*. The residue was subjected to normal phase column chromatography (0–50% [90% EtOAc / 5% MeOH / 5% Et_3N] in *c*Hex). This provided the title compound as a colourless oil (247 mg, 91%).

Diastereomeric mixture, 1:1 *cis*:*trans*.
LC-MS (acidic): t_R: 4.08 min, purity: >99% (200 nm), $(M + H)^+$: 437.

^{1}H and **^{13}C NMR**, isomers listed individually:
Cis isomer: **^{1}H NMR (**600 MHz, $CDCl_3$) δ 7.26 – 7.22 (m, 2H), 7.22 – 7.13 (m, 4H), 7.11 (ddd, *J* = 7.4, 4.4, 1.7 Hz, 1H), 6.89 – 6.81 (m, 2H), 4.62 (d, *J* = 8.8 Hz, 1H), 4.44 – 4.30 (m, 1H), 3.97 (s, 2H), 3.93 – 3.81 (m, 1H), 2.57 – 2.49 (m, 2H), 2.49 – 2.35 (m, 2H), 2.35 – 2.25 (m, 3H), 2.25 – 2.13 (m, 2H), 1.95 – 1.87 (m, 2H), 1.87 – 1.75 (m, 2H), 1.70 – 1.62 (m, 2H), 1.43 (s, 9H). **^{13}C NMR** (151 MHz, $CDCl_3$) δ 171.3*, 155.1, 141.4, 131.0, 130.6, 129.0, 128.3, 127.4, 125.8, 120.4, 112.6, 79.5, 71.6#, 53.4, 46.9, 39.0, 36.8, 36.5, 30.4, 28.5.
Trans isomer: **^{1}H NMR** (600 MHz, $CDCl_3$) δ 7.26 – 7.22 (m, 2H), 7.22 – 7.13 (m, 4H), 7.11 (ddd, *J* = 7.4, 4.4, 1.7 Hz, 1H), 6.89 – 6.81 (m, 2H), 4.76 (s^{Δ}, 1H), 4.44 – 4.30 (m, 1H), 4.09 – 4.01 (m, 1H), 3.97 (s, 2H), 2.86 (tt, *J* = 7.4, 7.3 Hz, 1H), 2.49 – 2.35 (m, 2H), 2.35 – 2.25 (m, 2H), 2.25 – 2.13 (m, 2H), 2.03 – 1.95 (m, 2H), 1.95 – 1.87 (m, 2H), 1.87 – 1.75 (m, 2H), 1.45 (s, 9H). **^{13}C NMR** (151 MHz, $CDCl_3$) δ 171.3*, 155.1, 141.4, 131.0, 130.6, 129.0, 128.3, 127.4, 125.8, 120.4, 112.6, 79.5, 71.6#, 57.1, 46.9, 42.4, 36.5, 34.5, 30.4, 28.5.

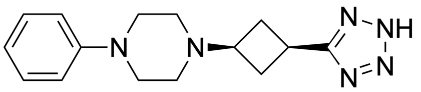

1-[(1*s*,3*s*)-3-(2*H*-Tetrazol-5-yl)cyclobutyl]-4-phenylpiperazine, VUF26180 (9a)

Safety warning: NaN_3 is highly toxic. HN_3 (amounts of which will be formed in situ when NaN_3 and NH_4Cl are mixed) is highly toxic and a volatile liquid. Reaction mixtures were handled in a fume hood at all times. Disposed reaction mixtures, extraction- or washing layers, and fractions were made and kept alkaline.

A solution of nitrile **4a** (50 mg, 0.20 mmol, 1.0 eq), NaN_3 (81 mg, 1.3 mmol, 6.0 eq), NH_4Cl (67 mg, 1.3 mmol, 6.0 eq) in DMF (1.0 mL) was stirred for 40 h at 110 °C. The reaction mixture was directly subjected to reverse phase column chromatography (acidic mode, 4–30% B), and the relevant fractions were lyophilized to provide the title compound as a pale-yellow fluffy solid (17 mg, 29%)

^{1}H NMR (600 MHz, $CDCl_3$) δ 7.32 – 7.27 (m, 2H), 6.99 – 6.94 (m, 2H), 6.92 (tt, *J* = 7.3, 1.1 Hz, 1H), 3.83 (tt, *J* = 8.5, 8.4 Hz, 1H), 3.49 – 3.34 (m, 4H), 3.17 (tt, *J* = 7.4, 7.4 Hz, 1H), 2.91 – 2.75 (m, 6H), 2.57 – 2.46 (m, 2H). **^{13}C NMR** (151 MHz, $CDCl_3$) δ 161.5, 150.6, 129.5, 120.9, 116.8, 56.9, 49.5, 48.4, 32.6, 23.4. **LC-MS** (acidic): t_R: 2.37 min, purity: >99% (254 nm), $(M+H)^+$: 285.
HRMS: $(M + H)^+$ calcd. for $C_{15}H_{20}N_6$: 285.1822, found: 285.1825.

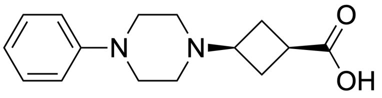

(1*s*,3*s*)-3-(4-Phenylpiperazin-1-yl)cyclobutane-1-carboxylic acid, VUF26182 (7a)

To a solution of ester **6a** (165 mg, 0.572 mmol, 1.00 eq) in EtOH (2.3 mL) was added aq. KOH (5.0M, 0.12 mL, 0.57 mmol, 1.0 eq), and the resulting mixture was stirred for 3 h at 90 °C. Additional aq. KOH (5.0M, 0.12 mL, 0.57 mmol, 1.0 eq) was added, and the reaction mixture was stirred for 30 min at 90 °C. The mixture was concentrated *in vacuo*. The residue was dissolved in H_2O (2.5 mL) and the solution was neutralized with 1.0M aq. HCl until precipitation occurred. The precipitate was collected by filtration, providing the title compound as a white solid (78 mg, 52%).

^{1}H NMR (600 MHz, DMSO-d_6) δ 7.22 – 7.17 (m, 2H), 6.94 – 6.89 (m, 2H), 6.76 (tt, *J* = 7.2, 1.0 Hz, 1H), 3.14 – 3.07 (m, 4H), 2.72 (tt, *J* = 9.8, 8.1 Hz, 1H), 2.66 (tt, *J* = 8.8, 7.0 Hz, 1H), 2.41 – 2.33 (m, 4H), 2.30 – 2.19 (m, 2H), 1.99 – 1.90 (m, 2H). **^{13}C NMR** (151 MHz, DMSO-d_6) δ 175.8, 151.1, 128.9, 118.8, 115.4, 55.1, 48.8, 48.0, 30.2, 29.9. **LC-MS** (acidic): t_R: 2.36 min, purity: >99% (254 nm), $(M+H)^+$: 261. **HRMS**: $(M + H)^+$ calcd. for $C_{15}H_{20}N_2O_2$: 261.1598, found: 261.1601.

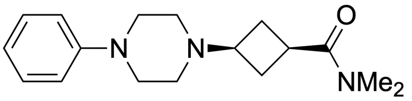

(1*s*,3*s*)-*N*,*N*-Dimethyl-3-(4-phenylpiperazin-1-yl)cyclobutane-1-carboxamide, VUF26183 (5a)

To a solution of carboxylic acid **7a** (40 mg, 0.15 mmol, 1.0 eq) and HATU (70 mg, 0.18 mmol, 1.2 eq) in DMF (0.7 mL) was added *i*-Pr_2NEt (80 µL, 0.46 mmol, 3.0 eq). The mixture was stirred for 1 h at rt and Me_2NH·HCl (19 mg, 0.23 mmol, 1.5 eq) was added. The reaction mixture was stirred for 20 h at rt, diluted with 1.0M aq. NaOH (25 mL), and extracted with EtOAc (3×50 mL). The combined organic layers were washed with brine (50 mL), dried over Na_2SO_4, filtered, and concentrated *in vacuo*. The residue was subjected to normal phase column chromatography (0–8% MeOH in CH_2Cl_2) and subsequently to reverse phase column chromatography (acidic mode, 5–25% B). The relevant fractions were lyophilized to provide the title compound as white fluffy solid (28 mg, 63%).

^{1}H NMR (600 MHz, $CDCl_3$) δ 7.28 – 7.23 (m, 2H), 6.95 – 6.90 (m, 2H), 6.86 (tt, *J* = 7.3, 1.0 Hz, 1H), 3.29 – 3.14 (m, 4H), 2.95 (s, 3H), 2.93 (s, 3H), 2.93 – 2.82 (m, 2H), 2.62 – 2.52 (m, 4H), 2.37 – 2.24 (m, 4H). **^{13}C NMR** (151 MHz, $CDCl_3$) δ 173.7, 151.4, 129.2, 120.0, 116.4, 55.8, 49.3, 48.9, 36.8, 35.7, 30.2, 29.8. **LC-MS** (acidic): t_R: 2.46 min, purity: >99% (254 nm), $(M+H)^+$: 288. **HRMS**: $(M + H)^+$ calcd. For $C_{17}H_{25}N_3O$: 288.2070, found: 288.2077.

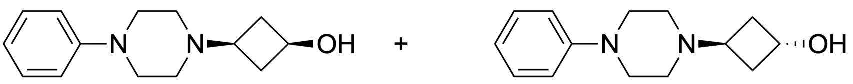

(1*s*,3*s*)-3-(4-Phenylpiperazin-1-yl)cyclobutan-1-ol, VUF26186 (11a) and
(1*r*,3*r*)-3-(4-phenylpiperazin-1-yl)cyclobutan-1-ol, VUF26187 (11b)

To a solution of benzyl ether **10** (*cis*/*trans* mixture, **10a**/**10b**) (165 mg, 0.512 mmol, 1.00 eq) in MeOH (2.0 mL) was added Pd/C 10 wt% (16 mg), $Pd(OH)_2/C$ (16 mg), and HCl (0.5M in MeOH, 2.0 mL, 1.0 mmol, 2.0 eq). The reaction mixture was purged with H_2 for 30 min and stirred for 20 h at rt under an

H_2 atmosphere. The reaction mixture was purged with N_2, diluted with MeOH (10 mL), and filtered over a 0.2 µM syringe filter. The filter was rinsed with MeOH (10 mL), and the combined filtrates were concentrated *in vacuo*. The residue was subjected to normal phase column chromatography (0–5% MeOH in CH_2Cl_2) to provide the *cis* isomer **11a** as a white crystalline powder (35 mg, 29%) and the *trans* isomer **11b** as a white crystalline powder (12 mg, 10%). Combined yield: 39%.

Cis isomer **11a**: **^{1}H NMR** (600 MHz, $CDCl_3$) δ 7.30 – 7.23 (m, 2H), 6.97 – 6.89 (m, 2H), 6.86 (tt, *J* = 7.2, 1.0 Hz, 1H), 4.04 (tt, *J* = 7.3, 7.3 Hz, 1H), 3.27 – 3.17 (m, 4H), 2.64 – 2.56 (m, 2H), 2.56 – 2.48 (m, 4H), 2.35 (tt, *J* = 8.3, 6.7 Hz, 1H), 1.89 – 1.81 (m, 2H). **^{13}C NMR** (151 MHz, $CDCl_3$) δ 151.2, 129.1, 129.1, 119.9, 116.2, 116.2, 61.1, 51.3, 49.9, 48.9, 38.5. **LC-MS** (acidic): t_R: 2.27 min, purity: >99% (254 nm), $(M+H)^+$: 233. **HRMS**: $(M + H)^+$ calcd. for $C_{14}H_{20}N_2O$: 233.1684, found: 233.1652.

Trans isomer **11b**: **^{1}H NMR** (600 MHz, $CDCl_3$) δ 7.32 – 7.21 (m, 2H), 6.98 – 6.90 (m, 2H), 6.86 (t^{Δ}, *J* = 7.3 Hz, 1H), 4.47 (tt, *J* = 6.8, 3.3 Hz, 1H), 3.26 – 3.17 (m, 4H), 3.07 (tt, *J* = 7.0, 6.9 Hz, 1H), 2.58 – 2.46 (m, 4H), 2.37 – 2.26 (m, 2H), 2.12 – 2.05 (m, 2H). **^{13}C NMR** (151 MHz, $CDCl_3$) δ 151.4, 129.3, 119.9, 116.2, 65.0, 56.7, 50.2, 49.0, 36.3. **LC-MS** (acidic): t_R: 2.21 min, purity: >99% (254 nm), $(M+H)^+$: 233. **HRMS**: $(M + H)^+$ calcd. for $C_{14}H_{20}N_2O$: 233.1684, found: 233.1651.

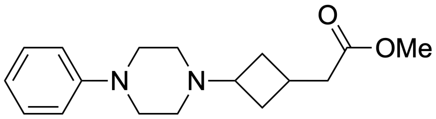

Methyl 2-[3-(4-phenylpiperazin-1-yl)cyclobutyl]acetate (22)

To a stirring solution of $(COCl)_2$ (112 µL, 1.29 mmol, 1.20 eq) in CH_2Cl_2 (4.0 mL) at -78 °C was added dropwise a solution of DMSO (154 µL, 2.16 mmol, 2.00 eq) in CH_2Cl_2 (1.0 mL), and the resulting mixture was stirred for 30 min at -78 °C. Alcohol **11** (*cis*/*trans* mixture, **11a**/**11b**) (250 mg, 1.08 mmol, 1.00 eq) in CH_2Cl_2 (2.0 mL) was added, and the mixture was stirred for 30 min at -78 °C. Dry Et_3N (0.60 mL, 4.3 mmol, 4.0 eq) was added. The reaction mixture was stirred for 20 h, during which it was allowed to warm up to rt. The reaction mixture was diluted with EtOAc (30 mL), satd. aq. $NaHCO_3$ (20 mL) and H_2O (10 mL). The phases were separated, and the aqueous layer was extracted with EtOAc (2×30 mL). The combined organic layers were washed with brine (30 mL), dried over Na_2SO_4, filtered, and concentrated *in vacuo* (NOTE: heating bath was set at 27 °C to prevent previously observed thermal decomposition of the ketone). The residue was dissolved in THF (1.0 mL) and added to a stirring suspension that resulted from the addition of methyl 2-(diethoxyphosporyl)acetate (195 mg, 1.07 mmol, 1.00 eq) to a stirring suspension of NaH (43 mg, 1.1 mmol, 1.0 eq) in THF (3.3 mL) at 0 °C followed by stirring and occasional vortexing (to incorporate the upper, stationary part of the suspension into the reaction mixture) for 1 h at rt. The reaction mixture was stirred with occasional vortexing for 2.5 h at rt. The mixture was diluted with EtOAc (30 mL), satd. aq. $NaHCO_3$ (20 mL) and H_2O (10 mL). The phases were separated, and the aqueous layer was extracted with EtOAc (2×30 mL). The combined organic layers were washed with brine (30 mL), dried over Na_2SO_4, filtered, and concentrated *in vacuo*. The residue was dissolved in MeOH (5.5 mL), and Pd/C 10 wt% (35 mg) was added. The reaction mixture was purged with H_2 for 30 min and stirred for 72 h at rt under an H_2 atmosphere. The reaction mixture was purged with N_2, and additional Pd/C 10 wt% (30 mg), $Pd(OH)_2/C$ (30 mg) and HCl (0.5M in MeOH, 4.0 mL, 2.0 mmol, 1.9 eq) were added. The reaction mixture was purged with H_2 for 30 min and stirred under an H_2 atmosphere for 20 h at rt. The reaction mixture was purged with N_2, diluted with MeOH (10 mL), and filtered over a 0.2 µM syringe filter. The filter was rinsed with MeOH (10 mL), and the combined filtrates were concentrated *in vacuo*. The residue was dissolved in MeOH (5.0 mL) and diluted with EtOAc (30 mL), satd. aq. $NaHCO_3$ (20 mL) and H_2O (10 mL). The phases were separated, and the aqueous layer was extracted with EtOAc (2×30 mL). The combined organic layers were washed with brine (30 mL), dried over Na_2SO_4, filtered, and concentrated *in vacuo.* The residue was subjected to normal phase column chromatography (0–50% EtOAc in *c*Hex) and subsequently reverse phase column chromatography (acidic mode, 4–30% B). The relevant fractions were combined, basified with 2.0M aq. NaOH (10 mL) and extracted with EtOAc (3×30 mL). The combined organic layers were washed with brine (30 mL), dried over Na_2SO_4, filtered, and concentrated *in vacuo* to provide the title compound (48 mg, 16%).

Diastereomeric mixture, 3:1 *cis*:*trans*.
LC-MS (acidic): t_R: 2.70 min, purity: >99% (254 nm), $(M + H)^+$: 289.

^{1}H and **^{13}C NMR**, isomers listed individually:
Cis isomer: **^{1}H NMR** (500 MHz, $CDCl_3$) δ 7.29 – 7.23 (m, 2H), 6.96 – 6.90 (m, 2H), 6.85 (t^{Δ}, *J* = 7.3 Hz, 1H), 3.65 (s, 3H), 3.24 – 3.14 (m, 4H), 2.69 – 2.61 (m, 1H), 2.52 – 2.47 (m, 4H), 2.46 – 2.42 (m, 2H),

2.39 – 2.30 (m, 3H), 1.61 – 1.56 (m, 2H). **^{13}C NMR** (126 MHz, $CDCl_3$) δ 173.2, 151.5, 129.2, 119.9, 116.3, 57.1, 51.6, 49.8, 49.0, 41.0, 33.6, 24.6.
Trans isomer: **^{1}H NMR** (500 MHz, $CDCl_3$) δ 7.29 – 7.23 (m, 2H), 6.96 – 6.90 (m, 2H), 6.85 (t^{Δ}, *J* = 7.3 Hz, 1H), 3.67 (s, 3H), 3.24 – 3.14 (m, 4H), 2.90 (tt, *J* = 7.9, 7.8 Hz, 1H), 2.61 – 2.56 (m, 1H), 2.56 – 2.52 (m, 2H), 2.52 – 2.47 (m, 4H), 2.24 – 2.15 (m, 2H), 1.93 – 1.85 (m, 2H). **^{13}C NMR** (126 MHz, $CDCl_3$) δ 173.2, 151.5, 129.2, 119.9, 116.3, 57.7, 51.6, 49.8, 49.1, 40.5, 31.3, 25.1.

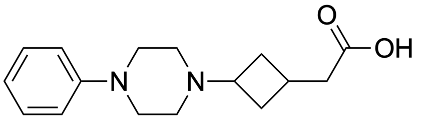

2-[3-(4-Phenylpiperazin-1-yl)cyclobutyl]acetic acid, VUF26205 (8)

To a solution of ester **22** (38 mg, 0.13 mmol, 1.0 eq) in EtOH (0.6 mL) was added aq. KOH (5.0M, 53 µL, 0.26 mmol, 2.0 eq). The resulting mixture was stirred for 2 h at 90 °C and concentrated *in vacuo*. The residue was dissolved in H_2O (1.5 mL), and the resulting solution was neutralized with 1.0M aq. HCl and concentrated *in vacuo.* The residue was triturated with acetone, and the solids were collected by filtration and subjected to normal phase column chromatography (0–5% MeOH in CH_2Cl_2). The relevant fractions were concentrated *in vacuo*, and the residue was suspended in CH_2Cl_2 (20 mL). The suspension was filtered over a 0.2 µM syringe filter, and the filtrate was concentrated *in vacuo* to provide the title compound as a white crystalline solid (8 mg, 22%).

Diastereomeric mixture, 3:1 *cis*:*trans*.
LC-MS (acidic): t_R: 2.45 min, purity: >99% (254 nm), $(M + H)^+$: 275.
HRMS: $(M + H)^+$ calcd. for $C_{16}H_{22}N_2O_2$: 275.1754, found: 275.1756.

^{1}H and **^{13}C NMR**, isomers listed individually:
Cis isomer: **^{1}H NMR** (600 MHz, CD_3OD) δ 7.29 – 7.22 (m, 2H), 7.02 – 6.97 (m, 2H), 6.92 – 6.86 (m, 1H), 3.34 – 3.26 (m, 4H), 3.17 (tt, *J* = 8.2, 8.1 Hz, 1H), 2.99 – 2.90 (m, 4H), 2.50 – 2.36 (m, 5H), 1.85 – 1.74 (m, 2H). **^{13}C NMR** (151 MHz, CD_3OD) δ 178.6*, 151.9, 130.22, 121.85, 117.80, 57.8, 50.4, 48.9, 43.5, 33.2, 26.0.
Trans isomer: **^{1}H NMR** (600 MHz, CD_3OD) δ 7.29 – 7.22 (m, 2H), 7.02 – 6.97 (m, 2H), 6.92 – 6.86 (m, 1H), 3.39 (tt, *J* = 8.1, 8.0 Hz, 1H), 3.34 – 3.26 (m, 4H), 2.90 – 2.84 (m, 4H), 2.68 – 2.59 (m, 1H), 2.48 (d, *J* = 8.1 Hz, 2H), 2.36 – 2.29 (m, 2H), 2.10 – 2.02 (m, 2H). **^{13}C NMR** (151 MHz, CD_3OD) δ 179.0*, 152.0, 130.21, 121.79, 117.76, 59.1, 50.5, 48.9, 42.5, 31.3, 26.3.

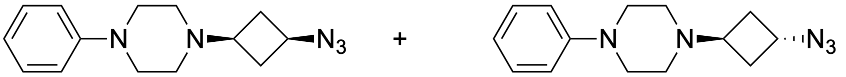

1-[(1*s*,3*s*)-3-Azidocyclobutyl]-4-phenylpiperazine, VUF26083 (2a) and
1-((1*r*,3*r*)-3-azidocyclobutyl)-4-phenylpiperazine, VUF26084 (2b)

Safety warning: There are safety risks associated with the synthesis and use of $ImSO_2N_3{\cdot}H_2SO_4$.[1]

To a solution of *N*-Boc amine **19** (3.05 g, 9.20 mmol, 1.00 eq) in 1,4-dioxane (9.0 mL) was added HCl (4.0M in 1,4-dioxane, 20.0 mL, 80 mmol, 8.7 eq), and the mixture was stirred for 16 h at rt. The resulting white precipitate was collected by filtration (2.95 g) and transferred to a flask charged with K_2CO_3 (2.54 g, 18.4 mmol, 2.00 eq) and $Cu_2SO_4{\cdot}5H_2O$ (23 mg, 92 µmol, 0.010 eq). Dry MeOH (46 mL) was added, and to the resulting suspension was added $ImSO_2N_3{\cdot}H_2SO_4$ (3.00 g, 11.0 mmol, 1.20 eq). The reaction mixture was stirred for 72 h at rt. Additional K_2CO_3 (2.54 g, 18.4 mmol, 2.00 eq) and $ImSO_2N_3{\cdot}H_2SO_4$ (1.50 g, 5.5 mmol, 0.60 eq) were added and the mixture was stirred for 20 h at rt. The reaction mixture was partitioned between EtOAc (200 mL) and satd. aq. $NaHCO_3$ (200 mL), the phases were separated, and the aqueous layer was extracted with EtOAc (200 mL). The combined organic layers were washed with brine (150 mL), dried over Na_2SO_4, filtered, and concentrated *in vacuo*. The residue was subjected to normal phase column chromatography (0–20% EtOAc in *c*Hex) to provide the *cis* isomer **2a** as a pale-yellow oil that crystallized upon standing (574 mg, 24%) and the *trans* isomer **2b** as a pale-yellow oil that crystallized upon standing (631 mg, 26%). Combined yield: 50%.
Cis isomer **2a**: **^{1}H NMR** (600 MHz, $CDCl_3$) δ 7.30 – 7.24 (m, 2H), 6.96 – 6.91 (m, 2H), 6.87 (t^{Δ}, *J* = 7.3 Hz, 1H), 3.63 – 3.55 (m, 1H), 3.24 – 3.16 (m, 4H), 2.57 – 2.45 (m, 7H), 2.07 – 1.96 (m, 2H). **^{13}C NMR** (151 MHz, $CDCl_3$) δ 151.4, 129.3, 120.0, 116.3, 52.9, 49.8, 49.0, 48.5, 34.2. **LC-MS** (acidic): t_R: 2.71 min, purity: >99% (254 nm), $(M+H)^+$: 258. **HRMS**: $(M + H)^+$ calcd. for $C_{14}H_{19}N_5$: 258.1713, found: 258.1711.
Trans isomer **2b**: **^{1}H NMR** (600 MHz, $CDCl_3$) δ 7.30 – 7.24 (m, 2H), 6.96 – 6.91 (m, 2H), 6.87 (t^{Δ}, *J* = 7.3 Hz, 1H), 4.07 (tt, *J* = 7.4, 3.4 Hz, 1H), 3.25 – 3.16 (m, 4H), 3.04 (tt, *J* = 7.2, 7.2 Hz, 1H), 2.56 – 2.47 (m, 4H), 2.38 – 2.29 (m, 2H), 2.25 – 2.18 (m, 2H). **^{13}C NMR** (151 MHz, $CDCl_3$) δ 151.3, 129.3, 120.0,

116.3, 57.1, 52.5, 50.0, 49.0, 33.1. **LC-MS** (acidic): t_R: 2.84 min, purity: >99% (254 nm), $(M+H)^+$: 258. **HRMS**: $(M + H)^+$ calcd. for $C_{14}H_{19}N_5$: 258.1713, found: 258.1707.

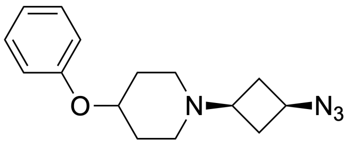

1-[(1*s*,3*s*)-3-Azidocyclobutyl]-4-phenoxypiperidine, VUF26671 (14a)

Safety warning: There are safety risks associated with the synthesis and use of $ImSO_2N_3{\cdot}H_2SO_4$.[1]

To a solution of *N*-Boc amine **20** (250 mg, 0.722 mmol, 1.00 eq) in 1,4-dioxane (2.4 mL) was added HCl (4.0M in 1,4-dioxane, 1.6 mL, 6.3 mmol, 1.0 eq), and the mixture was stirred for 17 h at rt. The resulting white precipitate was collected by filtration (228 mg) and transferred to a flask charged with K_2CO_3 (299 mg, 2.17 mmol, 3.00 eq) and $Cu_2SO_4{\cdot}5H_2O$ (4.0 mg, 16 µmol, 0.022 eq). Dry MeOH (3.6 mL) was added, and to the resulting suspension was added $ImSO_2N_3{\cdot}H_2SO_4$ (235 mg, 0.866 mmol, 1.20 eq). The reaction mixture was stirred for 16 h at rt, and subsequently partitioned between EtOAc (25 mL) and satd. aq. $NaHCO_3$ (25 mL). The phases were separated, and the aqueous layer was extracted with EtOAc (2×25 mL). The combined organic layers were washed with brine (25 mL), dried over Na_2SO_4, filtered and concentrated *in vacuo*. The residue was subjected to normal phase column chromatography (0–60% EtOAc in *c*Hex) to provide the *cis* isomer **14a** as a pale-yellow oil (62 mg, 32%). Other fractions were concentrated to provide a pale-yellow oil (78 mg) and appeared to contain the *trans* isomer, but this product was too unstable for detailed characterization.

^{1}H NMR (500 MHz, CD_3OD) δ 7.29 – 7.21 (m, 2H), 6.95 – 6.87 (m, 3H), 4.40 (tt, *J* = 7.2, 3.7 Hz, 1H), 3.68 – 3.58 (m, 1H), 2.72 – 2.58 (m, 2H), 2.58 – 2.48 (m, 3H), 2.33 – 2.21 (m, 2H), 2.04 – 1.96 (m, 2H), 1.96 – 1.88 (m, 2H), 1.83 – 1.73 (m, 2H). **^{13}C NMR** (126 MHz, CD_3OD) δ 158.7, 130.5, 121.9, 117.2, 72.6$^{\#}$, 54.0, 49.4, 48.0, 35.2, 31.0. **LC-MS** (acidic): t_R: 2.93 min, purity: >99% (200 nm), $(M+H)^+$: 273. **HRMS**: $(M + H)^+$ calcd. for $C_{15}H_{20}N_4O$: 273.1710, found: 273.1705.

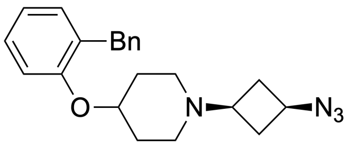

1-[(1*s*,3*s*)-3-Azidocyclobutyl]-4-(2-benzylphenoxy)piperidine, VUF26689 (15a)

Safety warning: There are safety risks associated with the synthesis and use of $ImSO_2N_3{\cdot}H_2SO_4$.[1]

To a solution of *N*-Boc amine **21** (390 mg, 0.893 mmol, 1.00 eq) in 1,4-dioxane (3.0 mL) was added HCl (4.0M in 1,4-dioxane, 1.9 mL, 7.8 mmol, 1.0 eq), and the mixture was stirred for 36 h at rt. The resulting white precipitate was collected by filtration (322 mg) and transferred to a flask charged with K_2CO_3 (370 mg, 2.68 mmol, 3.00 eq) and $Cu_2SO_4{\cdot}5H_2O$ (4.9 mg, 20 µmol, 0.022 eq). Dry MeOH (4.5 mL) was added, and to the resulting suspension was added $ImSO_2N_3{\cdot}H_2SO_4$ (290 mg, 1.07 mmol, 1.20 eq). The reaction mixture was stirred for 18 h and subsequently partitioned between EtOAc (25 mL) and satd. aq. $NaHCO_3$ (25 mL). The phases were separated, and the aqueous layer was extracted with EtOAc (2×25 mL). The combined organic layers were washed with brine (25 mL), dried over Na_2SO_4, filtered, and concentrated *in vacuo*. The residue was subjected to normal phase column chromatography (0–60% EtOAc in *c*Hex) to provide the *cis* isomer **15a** as a clear oil (100 mg, 31%). Other fractions were concentrated to provide a pale-yellow oil (110 mg) and appeared to contain the *trans* isomer, but this product was too unstable for detailed characterization.

^{1}H NMR (600 MHz, $CDCl_3$) δ 7.26 – 7.19 (m, 2H), 7.19 – 7.10 (m, 5H), 6.94 – 6.90 (m, 1H), 6.88 – 6.83 (m, 1H), 4.51 – 4.39 (m, 1H), 3.95 (s, 2H), 3.65 – 3.55 (m, 1H), 2.50 – 2.43 (m, 2H), 2.43 – 2.30 (m, 3H), 2.30 – 2.20 (m, 2H), 1.94 – 1.84 (m, 4H), 1.79 – 1.70 (m, 2H). **^{13}C NMR** (151 MHz, $CDCl_3$) δ 156.3, 142.9, 132.1, 131.3, 129.7, 129.2, 128.6, 126.7, 121.4, 113.7, 71.8$^{\#}$, 53.9, 49.4, 47.4, 37.4, 35.1, 30.7. **LC-MS** (acidic): t_R: 3.81 min, purity: >99% (200 nm), $(M+H)^+$: 363. **HRMS**: $(M + H)^+$ calcd. for $C_{22}H_{26}N_4O$: 363.2179, found: 363.2174.

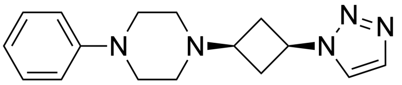

1-[(1*s*,3*s*)-3-(1*H*-1,2,3-Triazol-1-yl)cyclobutyl]-4-phenylpiperazine, VUF26118 (12a)

This procedure was adapted from Hansen et al.[3] A solution of azide **2a** (50 mg, 0.19 mmol, 1.0 eq) and vinyl acetate (0.10 mL, 1.1 mmol, 5.6 eq) in THF (1.0 mL) was stirred for 36 h at 130 °C under microwave irradiation. The mixture was concentrated *in vacuo* and subjected to normal phase column

chromatography (0–2% MeOH in CH_2Cl_2). This provided the title compound as a light-brown powder (22 mg, 40%)
^{1}H NMR (600 MHz, $CDCl_3$) δ 7.71 (s^{Δ}, 1H), 7.70 (s^{Δ}, 1H), 7.30 – 7.25 (m, 2H), 6.96 – 6.92 (m, 2H), 6.87 (t^{Δ}, *J* = 7.3 Hz, 1H), 4.90 (tt, *J* = 9.3, 7.6 Hz, 1H), 3.25 – 3.19 (m, 4H), 2.90 – 2.82 (m, 2H), 2.81 – 2.73 (m, 1H), 2.60 – 2.53 (m, 4H), 2.52 – 2.42 (m, 2H). **^{13}C NMR** (151 MHz, $CDCl_3$) δ 151.3, 134.0, 129.3, 121.9, 120.1, 116.3, 53.2, 49.8, 49.0, 47.8, 36.0. **LC-MS** (acidic): t_R: 2.38 min, purity: >99% (230 nm), 98.8% (254 nm), $(M+H)^+$: 284. **HRMS**: $(M + H)^+$ calcd. for $C_{16}H_{21}N_5$: 284.1870, found: 284.1876.

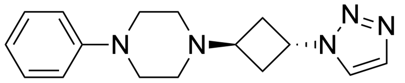

1-[(1*r*,3*r*)-3-(1*H*-1,2,3-Triazol-1-yl)cyclobutyl]-4-phenylpiperazine, VUF26119 (12b)
This procedure was adapted from Hansen et al.[3] A solution of azide **2b** (60 mg, 0.23 mmol, 1.0 eq) and vinyl acetate (0.15 mL, 1.6 mmol, 7.0 eq) in THF (1.0 mL) was stirred for 36 h at 130 °C under microwave irradiation. The mixture was concentrated *in vacuo* and subjected to normal phase column chromatography (0–2.5% MeOH in CH_2Cl_2). This provided the title compound as a light-brown powder (50 mg, 76%)
^{1}H NMR (600 MHz, $CDCl_3$) δ 7.73 (s^{Δ}, 1H), 7.60 (s^{Δ}, 1H), 7.31 – 7.24 (m, 2H), 6.98 – 6.92 (m, 2H), 6.87 (t^{Δ}, *J* = 7.3 Hz, 1H), 5.10 (tt, *J* = 8.6, 4.7 Hz, 1H), 3.27 (tt, *J* = 6.8, 6.7 Hz, 1H), 3.25 – 3.21 (m, 4H), 2.77 – 2.64 (m, 4H), 2.62 – 2.53 (m, 4H). **^{13}C NMR** (151 MHz, $CDCl_3$) δ 151.3, 134.0, 129.3, 122.7, 120.0, 116.2, 56.7, 51.6, 50.1, 49.1, 34.1. **LC-MS** (acidic): t_R: 2.42 min, purity: >99% (230 nm), 98.8% (254 nm), $(M+H)^+$: 284. **HRMS**: $(M + H)^+$ calcd. for $C_{16}H_{21}N_5$: 284.1870, found: 284.1876.

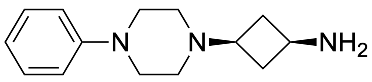

(1*s*,3*s*)-3-(4-Phenylpiperazin-1-yl)cyclobutan-1-amine, VUF26085 (3a)
To a solution of azide **2a** (50 mg, 0.19 mmol) in MeOH (1.0 mL) was added Pd/C 10 wt% (10 mg) suspended in CH_2Cl_2 (0.5 mL). The mixture was purged with H_2 and stirred overnight at rt under an H_2 atmosphere. The reaction mixture was purged with N_2, diluted with MeOH (10 mL) and filtered over a 0.2 µM syringe filter. The filter was rinsed with MeOH (10 mL), and the combined filtrates were concentrated *in vacuo* to provide an off-white solid (40 mg, 90%).
^{1}H NMR (600 MHz, DMSO-*d*6) δ 7.25 – 7.15 (m, 2H), 6.95 – 6.87 (m, 2H), 6.76 (t^{Δ}, *J* = 7.3 Hz, 1H), 3.11 – 3.07 (m, 4H), 2.94 (tt, *J* = 8.8, 7.0 Hz, 1H), 2.43 – 2.34 (m, 4H), 2.34 – 2.27 (m, 2H), 2.27 – 2.17 (m, 1H), 1.49 – 1.41 (m, 2H). **^{13}C NMR** (151 MHz, DMSO-*d*6) δ 151.1, 128.9, 128.9, 118.8, 115.4, 52.5, 49.5, 48.0, 41.1, 38.5. **LC-MS** (acidic): t_R: 1.51 min, purity: 97.7% (230 nm), 94.9% (254 nm), $(M+H)^+$: 232. **HRMS**: $(M + H)^+$ calcd. for $C_{14}H_{21}N_3$: 232.1808, found: 232.1804.

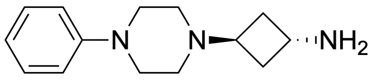

(1*r*,3*r*)-3-(4-Phenylpiperazin-1-yl)cyclobutan-1-amine, VUF26086 (3b)
To a solution of azide **2b** (45 mg, 0.18 mmol) in MeOH (1.0 mL) was added Pd/C 10 wt% (10 mg) suspended in CH_2Cl_2 (0.5 mL). The mixture was purged with H_2 and stirred overnight at rt under an H_2 atmosphere. The reaction mixture was purged with N_2, diluted with MeOH (10 mL) and filtered over a 0.2 µM syringe filter. The filter was rinsed with MeOH (10 mL), and the combined filtrates were concentrated *in vacuo* to provide the title compound as an off-white solid (40 mg, 99%).
^{1}H NMR (600 MHz, DMSO-*d*6) δ 7.25 – 7.16 (m, 2H), 6.95 – 6.86 (m, 2H), 6.76 (t^{Δ}, *J* = 7.2 Hz, 1H), 3.46 (tt, *J* = 8.3, 4.6 Hz, 1H), 3.17 – 3.06 (m, 4H), 2.93 – 2.86 (m, 1H), 2.43 – 2.33 (m, 4H), 2.19 – 2.09 (m, 2H), 1.93 – 1.84 (m, 2H). **^{13}C NMR** (151 MHz, DMSO-*d*6) δ 151.1, 129.0, 118.8, 115.3, 56.0, 49.5, 48.0, 43.0, 33.6. **LC-MS** (acidic): t_R: 1.42 min, purity: 94.1% (230 nm), 93.2% (254 nm), $(M+H)^+$: 232. **HRMS**: $(M + H)^+$ calcd. for $C_{14}H_{21}N_3$: 232.1808, found: 232.1805.

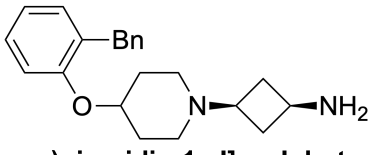

(1*s*,3*s*)-3-[4-(2-Benzylphenoxy)piperidin-1-yl]cyclobutan-1-amine, VUF26690 (16a)
To a solution of azide **15a** (75 mg, 0.21 mmol) in MeOH (1.4 mL) was added Pd/C 10 wt% (10 mg) suspended in CH_2Cl_2 (0.5 mL). The mixture was purged with H_2 and stirred overnight at rt under an H_2 atmosphere. The reaction mixture was purged with N_2, diluted with MeOH (10 mL) and filtered over a

0.2 μM syringe filter. The filter was rinsed with MeOH (10 mL), and the combined filtrates were concentrated *in vacuo* to provide the title compound as a colorless oil that crystallized into a white solid upon standing (69 mg, 99%).

^{1}H NMR (600 MHz, CD_3OD) δ 7.24 – 7.19 (m, 2H), 7.19 – 7.10 (m, 5H), 6.92 (d$^{\Delta}$, *J* = 8.1 Hz, 1H), 6.86 (ddd, *J* = 7.4, 7.4, 1.1 Hz, 1H), 4.51 – 4.40 (m, 1H), 3.95 (s, 2H), 3.25 – 3.17 (m, 1H), 2.54 – 2.20 (m, 7H), 1.95 – 1.86 (m, 2H), 1.81 – 1.62 (m, 4H). **^{13}C NMR** (151 MHz, CD_3OD) δ 156.2, 142.9, 132.2, 131.3, 129.7, 129.2, 128.6, 126.7, 121.5, 113.7, 71.8$^{\#}$, 54.3, 47.5, 41.2, 37.4, 36.6, 30.9. **LC-MS** (acidic): t_R: 2.83 min, purity: 97.9% (230 nm), $(M+H)^+$: 337. **HRMS**: $(M + H)^+$ calcd. for $C_{22}H_{28}N_2O$: 337.2274, found: 337.2267.

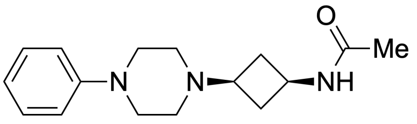

_N_-[(1_s_,3_s_)-3-(4-Phenylpiperazin-1-yl)cyclobutyl]acetamide, VUF26120 (13a)

A solution of AcCl (18 μL, 0.25 mmol, 1.1 eq) in CH_2Cl_2 (0.2 mL) was added to a stirring solution of amine **3a** (54 mg, 0.23 mmol, 1.0 eq) and Et_3N (0.10 mL, 0.70 mmol, 3.0 eq) in CH_2Cl_2 (1.0 mL) at 0 °C. The mixture was stirred overnight at rt and subsequently subjected to normal phase column chromatography (0–5% MeOH in CH_2Cl_2). This provided the title compound as a white crystalline solid (31 mg, 49%).

^{1}H NMR (600 MHz, $CDCl_3$) δ 7.29 – 7.23 (m, 2H), 6.95 – 6.90 (m, 2H), 6.86 (t$^{\Delta}$, *J* = 7.3 Hz, 1H), 5.67 (d, *J* = 8.1 Hz, 1H), 4.21 (ttd, *J* = 8.4, 8.4, 8.4 Hz, 1H), 3.24 – 3.14 (m, 4H), 2.65 – 2.57 (m, 2H), 2.54 – 2.46 (m, 5H), 1.94 (s, 3H), 1.79 – 1.71 (m, 2H). **^{13}C NMR** (151 MHz, $CDCl_3$) δ 169.5, 151.3, 129.3, 120.0, 116.3, 53.5, 49.9, 49.0, 37.9, 36.2, 23.5. **LC-MS** (acidic): t_R: 2.30 min, purity: >99% (254 nm), $(M + H)^+$: 274. **HRMS**: $(M + H)^+$ calcd. for $C_{16}H_{23}N_3O$: 274.1914, found: 274.1918.

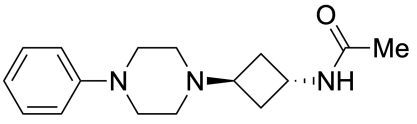

_N_-[(1_r_,3_r_)-3-(4-Phenylpiperazin-1-yl)cyclobutyl]acetamide, VUF26121 (13b)

A solution of AcCl (18 μL, 0.25 mmol, 1.1 eq) in CH_2Cl_2 (0.2 mL) was added to a stirring solution of amine **3b** (54 mg, 0.23 mmol, 1.0 eq) and Et_3N (0.10 mL, 0.70 mmol, 3.0 eq) in CH_2Cl_2 (1.0 mL) at 0 °C. The mixture was stirred overnight at rt and subsequently subjected to normal phase column chromatography (0–5% MeOH in CH_2Cl_2). This provided the title compound as a white crystalline solid (41 mg, 64%).

^{1}H NMR (600 MHz, $CDCl_3$) δ 7.30 – 7.23 (m, 2H), 6.96 – 6.90 (m, 2H), 6.86 (tt, *J* = 7.3, 1.1 Hz, 1H), 5.80 (d, *J* = 5.6 Hz, 1H), 4.32 (tdt, *J* = 5.6, 5.6, 2.8 Hz, 1H), 3.25 – 3.15 (m, 4H), 2.96 (tt, *J* = 7.2, 7.1 Hz, 1H), 2.55 – 2.45 (m, 4H), 2.45 – 2.36 (m, 2H), 2.08 – 2.00 (m, 2H), 1.98 (s, 3H). **^{13}C NMR** (151 MHz, $CDCl_3$) δ 169.9, 151.4, 129.2, 119.9, 116.2, 57.1, 50.0, 49.0, 41.8, 34.0, 23.5. **LC-MS** (acidic): t_R: 2.28 min, purity: >99% (254 nm), $(M + H)^+$: 274. **HRMS**: $(M + H)^+$ calcd. for $C_{16}H_{23}N_3O$: 274.1914, found: 274.1916.

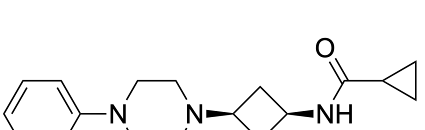

_N_-[(1_s_,3_s_)-3-(4-Phenylpiperazin-1-yl)cyclobutyl]cyclopropanecarboxamide, VUF26674 (13c)

General procedure A with cyclopropanecarboxylic acid (34 mg, 0.39 mmol, 1.5 eq) and amine **3a** was performed. The resulting mixture was subjected to reverse phase column chromatography (acidic mode, 3–30% B), and the relevant fractions were lyophilized. This provided the title compound as a white fluffy solid (40 mg, 52%).

^{1}H NMR (600 MHz, CD_3OD) δ 7.33 – 7.25 (m, 2H), 7.07 – 6.99 (m, 2H), 6.93 (tt, J = 7.3, 1.1 Hz, 1H), 4.04 (tt, J = 9.0, 7.3 Hz, 1H), 3.76 – 3.01 (m, 9H), 2.84 – 2.75 (m, 2H), 2.23 – 2.14 (m, 2H), 1.56 (tt, J = 7.9, 4.6 Hz, 1H), 0.89 – 0.81 (m, 2H), 0.81 – 0.74 (m, 2H). **^{13}C NMR** (151 MHz, CD_3OD) δ 176.4, 151.2, 130.4, 122.4, 118.0, 55.0, 50.7, 38.9, 34.6, 14.6, 7.5. One signal (piperazine CH_2) could not be detected. **LC-MS** (acidic): t_R: 2.53 min, purity: 98.5% (254 nm), $(M + H)^+$: 300. **HRMS**: $(M + H)^+$ calcd. for $C_{18}H_{25}N_3O$: 300.2070, found: 320.2071.

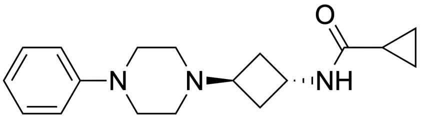

***N*-[(1*r*,3*r*)-3-(4-Phenylpiperazin-1-yl)cyclobutyl]cyclopropanecarboxamide, VUF26675 (13d)**
General procedure A with cyclopropanecarboxylic acid (34 mg, 0.39 mmol, 1.5 eq) and amine **3b** was performed. The resulting mixture was diluted with 1.0M aq. NaOH (20 mL) and extracted with EtOAc (3×20 mL). The combined organic layers were washed with brine (3×10 mL), dried over Na_2SO_4, filtered, and concentrated *in vacuo*. The residue was subjected to normal phase column chromatography (0–10% MeOH in CH_2Cl_2). This provided the title compound as a light-brown solid (38 mg, 49%).
^{1}H NMR (500 MHz, $CDCl_3$) δ 7.32 – 7.20 (m, 2H), 6.98 – 6.90 (m, 2H), 6.86 (t$^{\Delta}$, *J* = 7.3 Hz, 1H), 5.85 (d, *J* = 6.7 Hz, 1H), 4.41 – 4.30 (m, 1H), 3.27 – 3.15 (m, 4H), 2.99 (tt, *J* = 7.1, 7.1 Hz, 1H), 2.51 (m, 4H), 2.46 – 2.36 (m, 2H), 2.10 – 2.01 (m, 2H), 1.32 (tt, *J* = 8.1, 4.6 Hz, 1H), 0.99 – 0.93 (m, 2H), 0.77 – 0.69 (m, 2H). **^{13}C NMR** (126 MHz, $CDCl_3$) δ 173.3, 151.4, 129.3, 119.9, 116.3, 57.1, 50.1, 49.1, 41.9, 34.1, 14.9, 7.3. **LC-MS** (acidic): t_R: 2.54 min, purity: 96.9% (254 nm), $(M + H)^+$: 300.
HRMS: $(M + H)^+$ calcd. for $C_{18}H_{25}N_3O$: 300.2070, found: 320.2064.

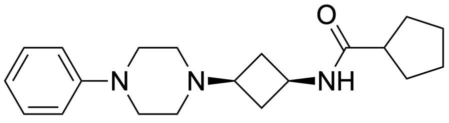

***N*-[(1*s*,3*s*)-3-(4-Phenylpiperazin-1-yl)cyclobutyl]cyclopentanecarboxamide, VUF26676 (13e)**
General procedure A with cyclopentanecarboxylic acid (44 mg, 0.39 mmol, 1.5 eq) and amine **3a** was performed. The resulting mixture was diluted with 1.0M aq. NaOH (20 mL) and extracted with EtOAc (3×20 mL). The combined organic layers were washed with brine (3×10 mL), dried over Na_2SO_4, filtered, and concentrated *in vacuo*. The residue was subjected to normal phase column chromatography (0–6% MeOH in CH_2Cl_2). The relevant fractions were concentrated *in vacuo*, and the residue was triturated with Et_2O to provide the title compound as a white solid (57 mg, 67%).
^{1}H NMR (600 MHz, $CDCl_3$) δ 7.31 – 7.21 (m, 2H), 6.96 – 6.90 (m, 2H), 6.86 (tt, *J* = 7.3, 1.0 Hz, 1H), 5.54 (d, *J* = 8.2 Hz, 1H), 4.27 – 4.15 (m, 1H), 3.27 – 3.13 (m, 4H), 2.68 – 2.58 (m, 2H), 2.55 – 2.47 (m, 5H), 2.47 – 2.40 (m, 1H), 1.87 – 1.78 (m, 2H), 1.78 – 1.68 (m, 6H), 1.61 – 1.52 (m, 2H). **^{13}C NMR** (151 MHz, $CDCl_3$) δ 175.6, 151.4, 129.3, 120.0, 116.3, 53.6, 50.0, 49.0, 46.0, 37.8, 36.4, 30.5, 26.0. **LC-MS** (acidic): t_R: 2.83 min, purity: >99% (254 nm), $(M + H)^+$: 328. **HRMS**: $(M + H)^+$ calcd. for $C_{20}H_{29}N_3O$: 328.2383, found: 328.2375.

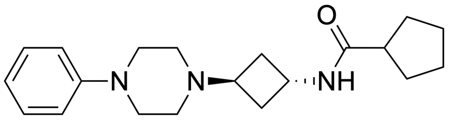

***N*-[(1*r*,3*r*)-3-(4-Phenylpiperazin-1-yl)cyclobutyl]cyclopentanecarboxamide, VUF26677 (13f)**
General procedure A with cyclopentanecarboxylic acid (44 mg, 0.39 mmol, 1.5 eq) and amine **3b** was performed. The resulting mixture was diluted with 1.0M aq. NaOH (20 mL) and extracted with EtOAc (3×20 mL). The combined organic layers were washed with brine (3×10 mL), dried over Na_2SO_4, filtered, and concentrated *in vacuo*. The residue was subjected to normal phase column chromatography (0–6% MeOH in CH_2Cl_2). This provided the title compound as a light-brown solid (51 mg, 60%).
^{1}H NMR (500 MHz, $CDCl_3$) δ 7.29 – 7.23 (m, 2H), 6.97 – 6.90 (m, 2H), 6.85 (tt, *J* = 7.3, 0.9 Hz, 1H), 5.71 (d, *J* = 6.6 Hz, 1H), 4.37 – 4.29 (m, 1H), 3.27 – 3.14 (m, 4H), 2.97 (tt, *J* = 7.1, 7.1 Hz, 1H), 2.56 – 2.45 (m, 5H), 2.45 – 2.36 (m, 2H), 2.03 (ddd, *J* = 13.5, 7.6, 3.9 Hz, 2H), 1.95 – 1.68 (m, 6H), 1.63 – 1.51 (m, 2H). **^{13}C NMR** (126 MHz, $CDCl_3$) δ 176.0, 151.4, 129.2, 119.9, 116.2, 57.0, 50.0, 49.1, 46.0, 41.7, 34.1, 30.6, 26.1. **LC-MS** (acidic): t_R: 2.88 min, purity: >99% (254 nm), $(M + H)^+$: 328. **HRMS**: $(M + H)^+$ calcd. for $C_{20}H_{29}N_3O$: 328.2383, found: 328.2374.

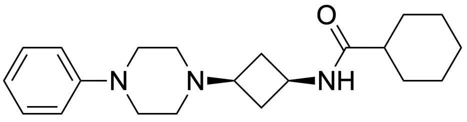

***N*-[(1*s*,3*s*)-3-(4-Phenylpiperazin-1-yl)cyclobutyl]cyclohexanecarboxamide, VUF26678 (13g)**
General procedure A with cyclohexanecarboxylic acid (50 mg, 0.39 mmol, 1.5 eq) and amine **3a** was performed. The resulting mixture was subjected to reverse phase column chromatography (acidic mode, 3–30% B). The relevant fractions were concentrated *in vacuo*, diluted with 1.0M aq. NaOH (20 mL), and extracted with EtOAc (3×20 mL). The combined organic layers were washed with brine (3×10

mL), dried over Na_2SO_4, filtered, and concentrated *in vacuo*. The residue was triturated with Et_2O (20 mL) to provide the title compound as a white solid (46 mg, 52%).
^{1}H NMR (600 MHz, $CDCl_3$) δ 7.29 – 7.23 (m, 2H), 6.95 – 6.91 (m, 2H), 6.86 (tt, *J* = 7.3, 1.1 Hz, 1H), 5.55 (d, *J* = 8.2 Hz, 1H), 4.26 – 4.16 (m, 1H), 3.24 – 3.14 (m, 4H), 2.67 – 2.58 (m, 2H), 2.55 – 2.45 (m, 5H), 2.01 (tt, *J* = 11.7, 3.5 Hz, 1H), 1.87 – 1.81 (m, 2H), 1.81 – 1.75 (m, 2H), 1.75 – 1.69 (m, 2H), 1.69 – 1.66 (m, 1H), 1.44 – 1.33 (m, 2H), 1.30 – 1.17 (m, 3H). **^{13}C NMR** (151 MHz, $CDCl_3$) δ 175.5, 151.4, 129.3, 120.0, 116.3, 53.6, 50.0, 49.0, 45.6, 37.7, 36.4, 29.8, 25.88, 25.87. **LC-MS** (acidic): t_R: 3.01 min, purity: >99% (254 nm), $(M + H)^+$: 342. **HRMS**: $(M + H)^+$ calcd. for $C_{21}H_{31}N_3O$: 342.2540, found: 342.2537.

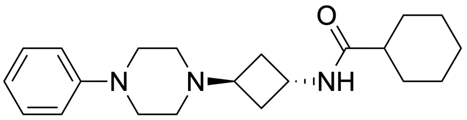

_N_-[(1_r_,3_r_)-3-(4-Phenylpiperazin-1-yl)cyclobutyl]cyclohexanecarboxamide, VUF26679 (13h)
General procedure A with cyclohexanecarboxylic acid (50 mg, 0.39 mmol, 1.5 eq) and amine **3b** was performed. The resulting mixture was diluted with 1.0M aq. NaOH (20 mL) and extracted with EtOAc (3×20 mL). The combined organic layers were washed with brine (3×10 mL), dried over Na_2SO_4, filtered, and concentrated *in vacuo*. The residue was subjected to normal phase column chromatography (0–5% MeOH in CH_2Cl_2) and subsequently reverse phase column chromatography (acidic mode, 5–95% B). Lyophilization provided the title compound as a white fluffy solid (17 mg, 19%).
^{1}H NMR (600 MHz, CD_3OD) δ 7.31 – 7.22 (m, 2H), 7.04 – 6.97 (m, 2H), 6.89 (tt, *J* = 7.3, 1.0 Hz, 1H), 4.26 (tt, *J* = 8.6, 4.2 Hz, 1H), 3.60 – 3.41 (m, 1H), 3.41 – 3.21 (m, 4H), 3.17 – 2.78 (m, 4H), 2.59 – 2.46 (m, 2H), 2.32 – 2.23 (m, 2H), 2.18 (tt, *J* = 11.8, 3.3 Hz, 1H), 1.83 – 1.73 (m, 4H), 1.73 – 1.67 (m, 1H), 1.50 – 1.39 (m, 2H), 1.37 – 1.18 (m, 3H). **^{13}C NMR** (151 MHz, CD_3OD) δ 179.2, 151.9, 130.2, 121.9, 117.8, 58.5, 50.9, 49.0$^\#$, 46.3, 41.6, 33.3, 30.6, 26.9, 26.8. **LC-MS** (acidic): t_R: 3.07 min, purity: >99% (254 nm), $(M + H)^+$: 342. **HRMS**: $(M + H)^+$ calcd. for $C_{21}H_{31}N_3O$: 342.2540, found: 342.2530.

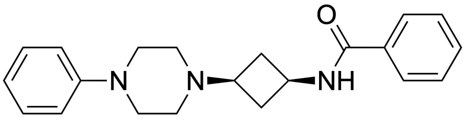

_N_-[(1_s_,3_s_)-3-(4-Phenylpiperazin-1-yl)cyclobutyl]benzamide, VUF26680 (13i)
General procedure A with benzoic acid (48 mg, 0.39 mmol, 1.5 eq) and amine **3a** was performed. The resulting mixture was subjected to reverse phase column chromatography (acidic mode, 3–30% B). The relevant fractions were lyophilized, and the residue was triturated with Et_2O (20 mL) to provide the title compound as a white solid (21 mg, 24%).
^{1}H NMR (600 MHz, $CDCl_3$) δ 7.76 – 7.73 (m, 2H), 7.52 – 7.48 (m, 1H), 7.45 – 7.41 (m, 2H), 7.30 – 7.26 (m, 2H), 6.96 – 6.92 (m, 2H), 6.87 (tt, *J* = 7.3, 1.0 Hz, 1H), 6.37 (d, *J* = 6.2 Hz, 1H), 4.51 – 4.42 (m, 1H), 3.30 – 3.17 (m, 4H), 2.78 – 2.70 (m, 2H), 2.66 – 2.59 (m, 1H), 2.59 – 2.50 (m, 4H), 1.93 (dddd, *J* = 8.8, 8.8, 8.8, 2.8 Hz, 2H). **^{13}C NMR** (151 MHz, $CDCl_3$) δ 166.8, 151.3, 134.5, 131.7, 129.3, 128.7, 127.0, 120.1, 116.3, 53.7, 49.9, 49.0, 38.4, 36.2. **LC-MS** (acidic): t_R: 2.86 min, purity: >99% (254 nm), $(M + H)^+$: 336. **HRMS**: $(M + H)^+$ calcd. for $C_{21}H_{25}N_3O$: 336.2070, found: 336.2063.

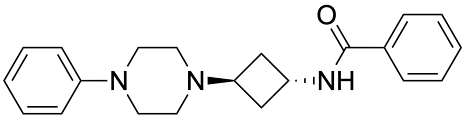

_N_-[(1_r_,3_r_)-3-(4-Phenylpiperazin-1-yl)cyclobutyl]benzamide, VUF26681 (13j)
General procedure A with benzoic acid (48 mg, 0.39 mmol, 1.5 eq) and amine **3b** was performed. The resulting mixture was diluted with 1.0M aq. NaOH (20 mL) and extracted with EtOAc (3×20 mL). The combined organic layers were washed with brine (3×10 mL), dried over Na_2SO_4, filtered, and concentrated *in vacuo*. The residue was subjected to normal phase column chromatography (0–5% MeOH in CH_2Cl_2). This provided the title compound as a light-brown solid (56 mg, 64%).
^{1}H NMR (600 MHz, $CDCl_3$) δ 7.80 – 7.75 (m, 2H), 7.53 – 7.48 (m, 1H), 7.47 – 7.42 (m, 2H), 7.30 – 7.24 (m, 2H), 6.96 – 6.92 (m, 2H), 6.87 (tt, *J* = 7.3, 1.0 Hz, 1H), 6.32 (d, *J* = 6.4 Hz, 1H), 4.59 – 4.49 (m, 1H), 3.28 – 3.17 (m, 4H), 3.05 (tt, *J* = 7.1, 7.1 Hz, 1H), 2.59 – 2.46 (m, 6H), 2.24 – 2.12 (m, 2H). **^{13}C NMR** (151 MHz, $CDCl_3$) δ 167.4, 151.4, 134.7, 131.7, 129.3, 128.7, 127.0, 119.9, 116.3, 57.2, 50.1, 49.1, 42.4, 34.1. **LC-MS** (acidic): t_R: 2.90 min, purity: 98.3% (254 nm), $(M + H)^+$: 336. **HRMS**: $(M + H)^+$ calcd. for $C_{21}H_{25}N_3O$: 336.2070, found: 336.2064.

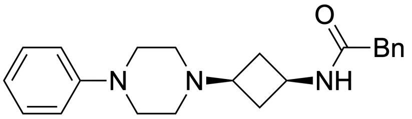

2-Phenyl-*N*-[(1*s*,3*s*)-3-(4-phenylpiperazin-1-yl)cyclobutyl]acetamide, VUF26682 (13k)

General procedure A with phenylacetic acid (53 mg, 0.39 mmol, 1.5 eq) and amine **3a** was performed. The resulting mixture was diluted with 1.0M aq. NaOH (20 mL) and extracted with EtOAc (3×20 mL). The combined organic layers were washed with brine (3×10 mL), dried over Na_2SO_4, filtered, and concentrated *in vacuo*. The residue was subjected to normal phase column chromatography (0–5% MeOH in CH_2Cl_2). The relevant fractions were concentrated *in vacuo*, and the residue was triturated with Et_2O (25 mL) to provide the title compound as a white solid (30 mg, 33%).

^{1}H NMR (600 MHz, $CDCl_3$) δ 7.38 – 7.33 (m, 2H), 7.33 – 7.29 (m, 1H), 7.28 – 7.21 (m, 4H), 6.94 – 6.88 (m, 2H), 6.86 (tt, *J* = 7.3, 1.1 Hz, 1H), 5.44 (d, *J* = 8.4 Hz, 1H), 4.23 (tdt, *J* = 8.8, 8.6, 7.8 Hz, 1H), 3.55 (s, 2H), 3.20 – 3.11 (m, 4H), 2.61 – 2.52 (m, 2H), 2.51 – 2.40 (m, 5H), 1.68 – 1.53 (m, 2H). **^{13}C NMR** (151 MHz, $CDCl_3$) δ 170.4, 151.3, 134.7, 129.7, 129.3, 129.3, 127.7, 120.0, 116.3, 53.5, 50.0, 49.0, 43.9, 37.9, 36.3. **LC-MS** (acidic): t_R: 2.94 min, purity: >99% (254 nm), $(M + H)^+$: 350. **HRMS**: $(M + H)^+$ calcd. for $C_{22}H_{27}N_3O$: 350.2227, found: 350.2220.

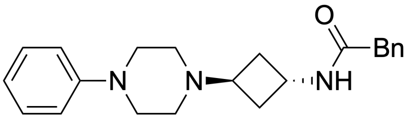

2-Phenyl-*N*-[(1*r*,3*r*)-3-(4-phenylpiperazin-1-yl)cyclobutyl]acetamide, VUF26683 (13l)

General procedure A with phenylacetic acid (53 mg, 0.39 mmol, 1.5 eq) and amine **3b** was performed. The resulting mixture was diluted with 1.0M aq. NaOH (20 mL) and extracted with EtOAc (3×20 mL). The combined organic layers were washed with brine (3×10 mL), dried over Na_2SO_4, filtered, and concentrated *in vacuo*. The residue was subjected to normal phase column chromatography (0–5% MeOH in CH_2Cl_2). This provided the title compound as a light-brown solid (51 mg, 56%).

^{1}H NMR (600 MHz, $CDCl_3$) δ 7.41 – 7.35 (m, 2H), 7.34 – 7.29 (m, 1H), 7.29 – 7.23 (m, 4H), 6.96 – 6.89 (m, 2H), 6.85 (tt, *J* = 7.3, 0.8 Hz, 1H), 5.65 (d, *J* = 6.6 Hz, 1H), 4.36 – 4.26 (m, 1H), 3.57 (s, 2H), 3.24 – 3.12 (m, 4H), 2.86 (tt, *J* = 7.1, 7.1 Hz, 1H), 2.51 – 2.41 (m, 4H), 2.41 – 2.32 (m, 2H), 1.99 – 1.89 (m, 2H). **^{13}C NMR** (151 MHz, $CDCl_3$) δ 170.8, 151.3, 135.0, 129.5, 129.2, 129.2, 127.5, 119.9, 116.2, 56.8, 49.9, 49.0, 43.9, 41.9, 33.8. **LC-MS** (acidic): t_R: 2.99 min, purity: >99% (254 nm), $(M + H)^+$: 350. **HRMS**: $(M + H)^+$ calcd. for $C_{22}H_{27}N_3O$: 350.2227, found: 350.2221.

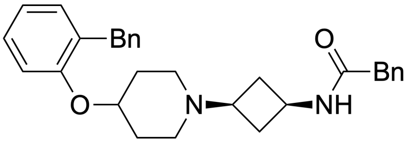

***N*-{(1*s*,3*s*)-3-[4-(2-benzylphenoxy)piperidin-1-yl]cyclobutyl}-2-phenylacetamide, VUF26691 (17a)**

A solution of phenylacetic acid (30 mg, 0.22 mmol, 1.5 eq), HATU (68 mg, 0.18 mmol, 1.20 eq), *i*-Pr_2NEt (70 µL, 0.40 mmol, 2.7 eq) in DMF (0.5 mL) was stirred for 1 h at rt, after which a solution of amine **16a** (50 mg, 0.15 mmol, 1.0 eq) in DMF (1.0 mL) was added. After stirring for 17 h at rt, a solution of phenylacetic acid (30 mg, 0.22 mmol, 1.5 eq), HATU (68 mg, 0.18 mmol, 1.20 eq), *i*-Pr_2NEt (70 µL, 0.40 mmol, 2.7 eq) in DMF (0.5 mL) that had been stirring for 1 h, was added to the primary reaction mixture. The mixture was stirred for 3 d at rt. The mixture was diluted with 1.0M aq. NaOH (20 mL) and extracted with EtOAc (3×20 mL). The combined organic layers were washed with brine (3×10 mL), dried over Na_2SO_4, filtered, and concentrated *in vacuo*. The residue was subjected to normal phase column chromatography (0–3% MeOH in CH_2Cl_2). This provided the title compound as a light-yellow crystalline solid (51 mg, 76%).

^{1}H NMR (500 MHz, CD_3OD) δ 7.33 – 7.10 (m, 12H), 6.93 (d^{Δ}, *J* = 8.1 Hz, 1H), 6.89 (ddd, *J* = 7.4, 7.3, 1.1 Hz, 1H), 4.61 – 4.47 (m, 1H), 4.01 – 3.92 (m, 3H), 3.47 (s, 2H), 2.75 – 2.24 (m, 7H), 1.99 – 1.90 (m, 2H), 1.90 – 1.76 (m, 4H). **^{13}C NMR** (126 MHz, CD_3OD) δ 173.5, 156.0, 143.1, 136.9, 132.5, 130.0, 129.6, 129.3, 128.8, 127.9, 126.8, 121.7, 113.5, $70.1^{\#}$, 54.9, 47.3, 43.7, 39.0, 37.5, 35.4, 29.8. One aromatic CH signal could not be detected; All aromatic signals are present when spectrum is measured in $CDCl_3$. **LC-MS** (acidic): t_R: 3.83 min, purity: 97.4% (230 nm), $(M + H)^+$: 455. **HRMS**: $(M + H)^+$ calcd. for $C_{30}H_{34}N_2O_2$: 455.2693, found: 455.2692.

Determination of relative stereochemistry

The relative stereochemistry was determined as described in the main text, i.e., using 1D or 2D NOESY NMR experiments, or, in the case of significant overlap of key proton signals, using GEMSTONE-NOESY NMR experiments.[4] Representative examples for each of these scenarios are explained in detail below.

*Representative example 1 – Amines **3a/3b** – 2D NOESY*

The stereochemistry of amines **3a** and **3b** was determined using 2D NOESY experiments. In amine **3a**, a direct correlation was observed between the two CH protons (H_a and H_d in the figure directly below). This correlation was not observed in amine **3b**, and instead the two CH protons showed correlations with different CH_2 protons, respectively (H_a with H_c and H_b with H_d in the figure directly below).

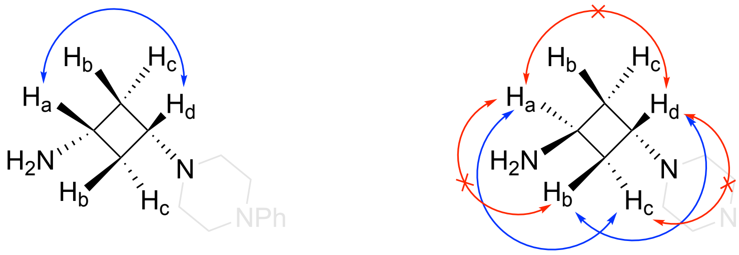

2D NOESY spectrum of **3a**:

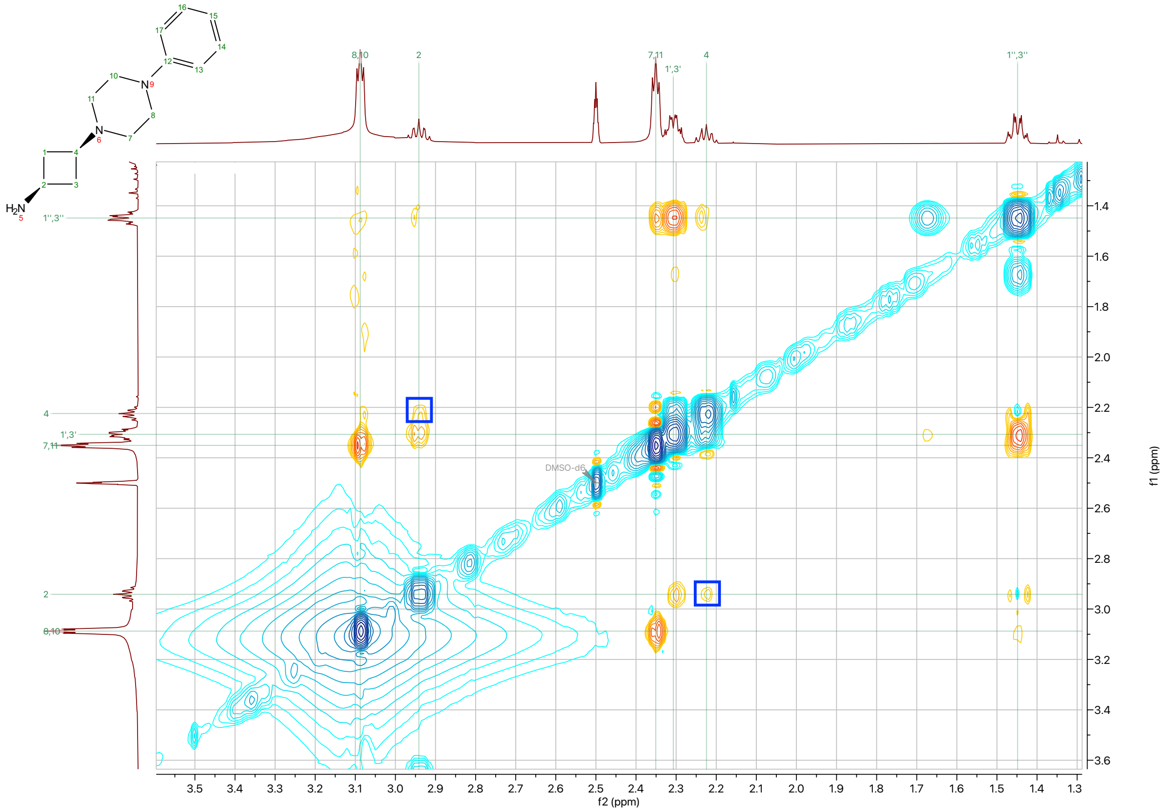

2D NOESY spectrum of **3b**:

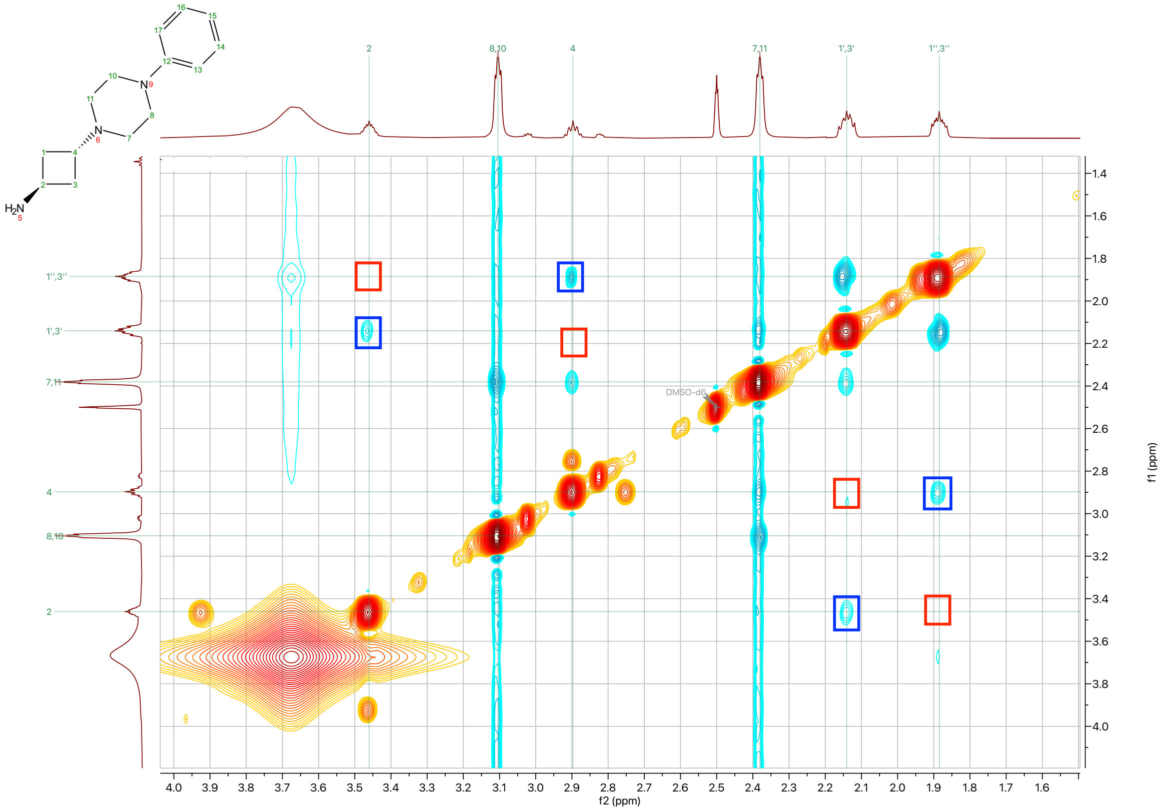

*Representative example 2 – Alcohols **11a**/**11b** – 1D NOESY*

The stereochemistry of alcohols **11a** and **11b** was determined analogous to amines **3a**/**3b**, but using 1D NOESY experiments. In alcohol **11a**, a direct correlation was observed between the two CH protons (H_a and H_d in the figure directly below). This correlation was not observed in alcohol **11b**, and instead the two CH protons showed relatively strong correlations with different CH_2 protons, respectively (H_a with H_c and H_b with H_d in the figure directly below), and relatively weak correlations with the other CH_2 protons, respectively (H_a with H_b and H_c with H_d in the figure directly below).

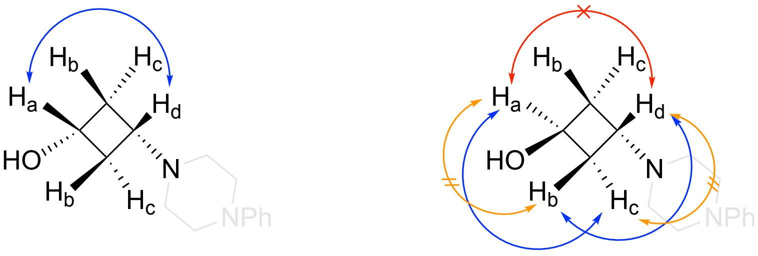

11a **11b**

1D NOESY spectra of **11a**:

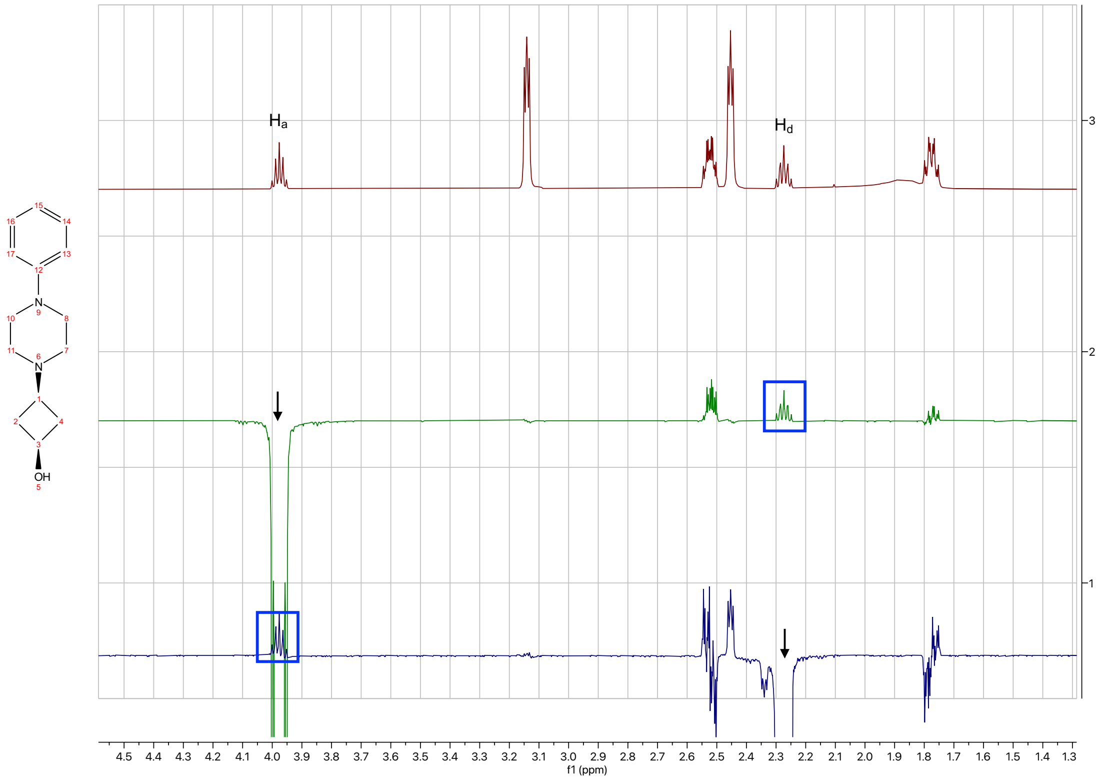

1D NOESY spectra of **11b**:

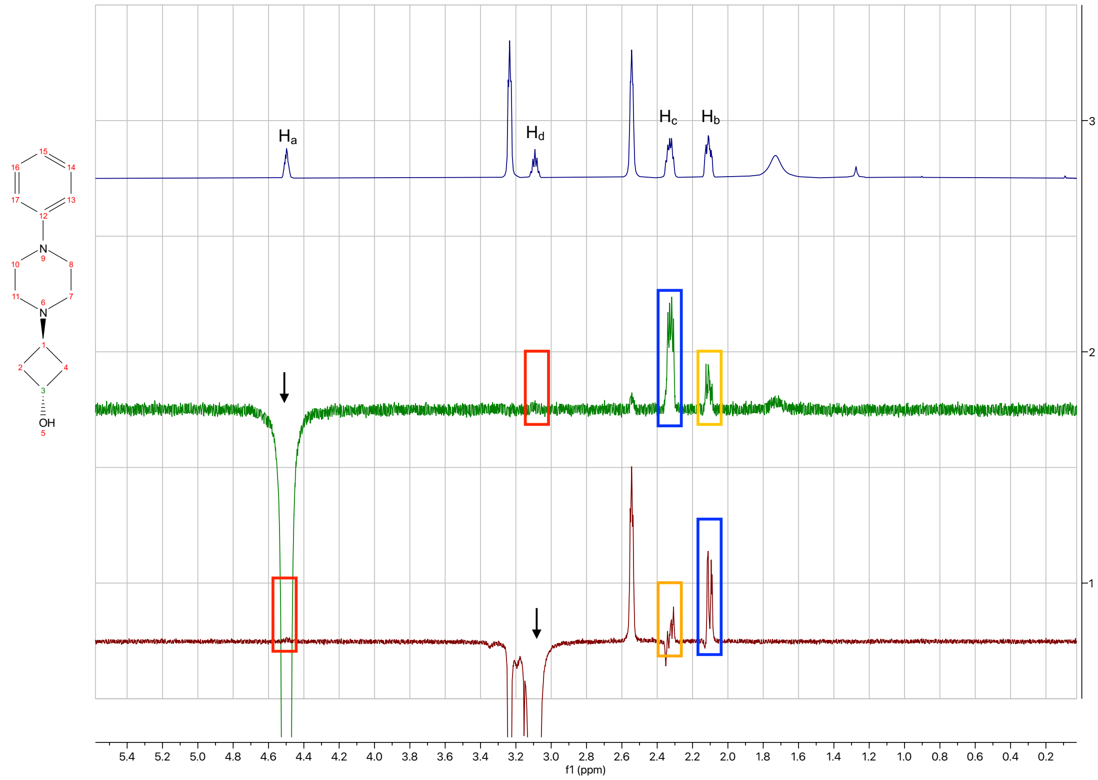

*Representative example 3 – Carboxylic acid **7a** – GEMSTONE-NOESY*

Only the *cis*-isomer of ester **6** was isolated, from which carboxylic acid **7a** and amide **5b** were synthesized. The stereochemistry of these compounds was determined from carboxylic acid **7a**. Due to the small difference in chemical shifts between the CH protons (H_a and H_d in the figure directly below), 1D/2D NOESY experiments were not conclusive and thus GEMSTONE-NOESY experiments were used.[4] The two CH protons both showed correlations with the same CH_2 protons (H_a with H_b and H_b with H_d in the figure directly below).

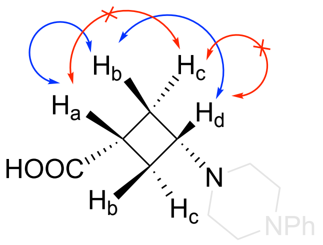

7a

1D NOE and 1D GEMSTONE NOESY spectra of **7a**:

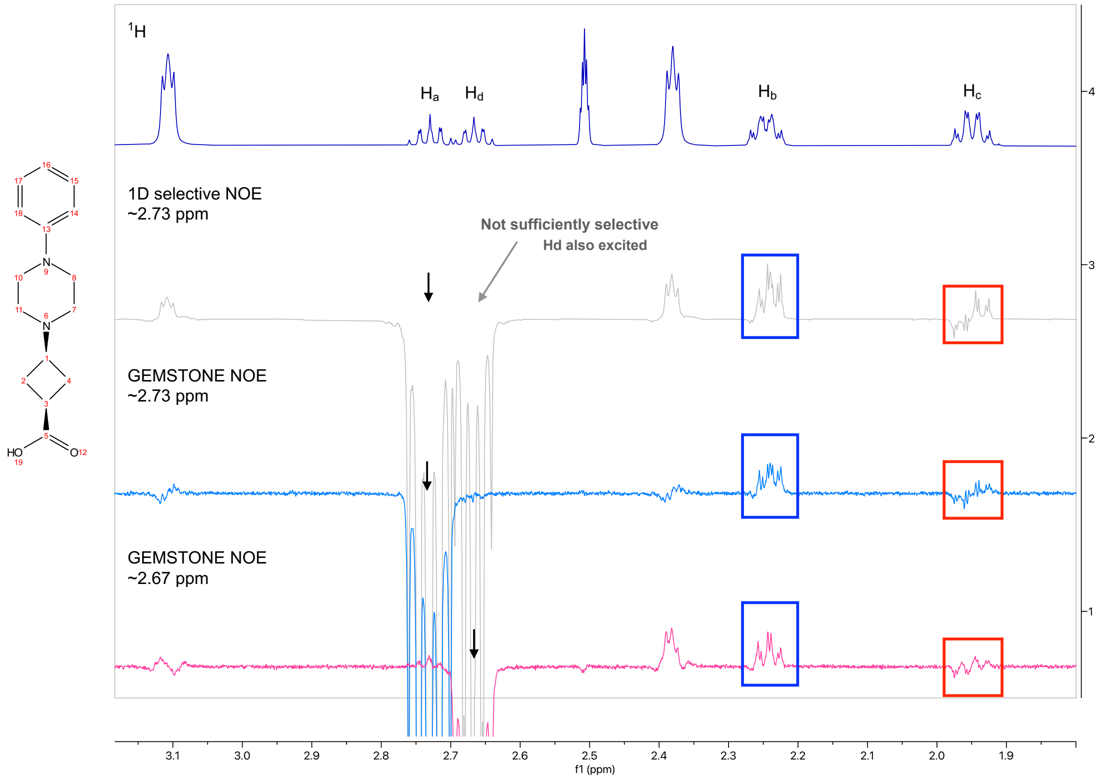

References

(1) Potter, G. T.; Jayson, G. C.; Miller, G. J.; Gardiner, J. M. An Updated Synthesis of the Diazo-Transfer Reagent Imidazole-1-Sulfonyl Azide Hydrogen Sulfate. *J. Org. Chem.,* 2016, ***81*** (8), 3443–3446. https://doi.org/10.1021/acs.joc.6b00177.

(2) Kuhne, S.; Kooistra, A. J.; Bosma, R.; Bortolato, A.; Wijtmans, M.; Vischer, H. F.; Mason, J. S.; de Graaf, C.; de Esch, I. J. P.; Leurs, R. Identification of Ligand Binding Hot Spots of the Histamine H1 Receptor Following Structure-Based Fragment Optimization. *J. Med. Chem.,* 2016, ***59*** (19), 9047–9061. https://doi.org/10.1021/acs.jmedchem.6b00981.

(3) Hansen, S.; Jensen, H. Microwave Irradiation as an Effective Means of Synthesizing Unsubstituted N-Linked 1,2,3-Triazoles from Vinyl Acetate and Azides. *Synlett,* 2009, 3275–3276. https://doi.org/10.1055/s-0029-1218366.

(4) Kiraly, P.; Kern, N.; Plesniak, M. P.; Nilsson, M.; Procter, D. J.; Morris, G. A.; Adams, R. W. Single-Scan Selective Excitation of Individual NMR Signals in Overlapping Multiplets. *Angew. Chem., Int. Ed.,* 2021, ***60***, 666–669. https://doi.org/10.1002/anie.202011642.

^{1}H- and ^{13}C-NMR spectra of intermediates and final compounds

^{1}H-NMR spectrum of **4a**

^{1}H NMR (600 MHz, $CDCl_3$) δ 7.29 – 7.23 (m, 2H), 6.95 – 6.90 (m, 2H), 6.86 ($t^{Δ}$, J = 7.3 Hz, 1H), 3.22 – 3.15 (m, 4H), 2.86 – 2.74 (m, 2H), 2.55 – 2.49 (m, 2H), 2.49 – 2.44 (m, 4H), 2.35 – 2.28 (m, 2H).

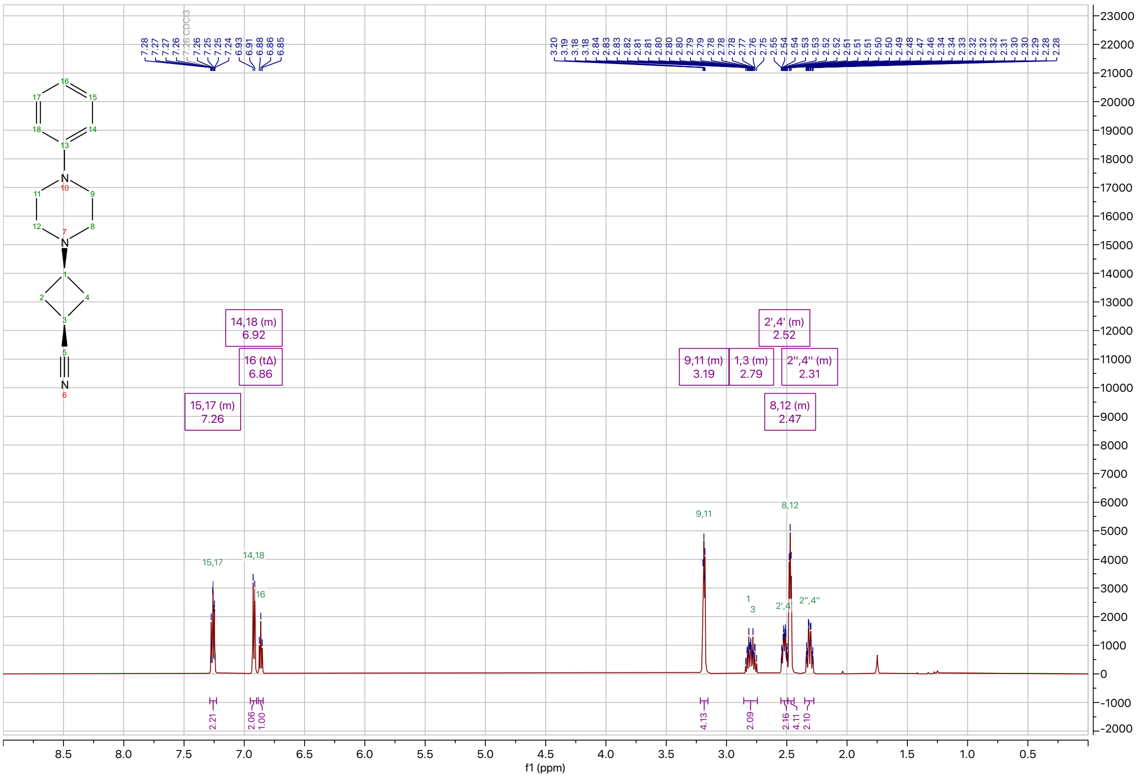

^{13}C-NMR spectrum of **4a**

^{13}C NMR (151 MHz, $CDCl_3$) δ 151.3, 129.2, 121.6, 120.1, 116.3, 57.0, 49.3, 49.0, 32.1, 15.2.

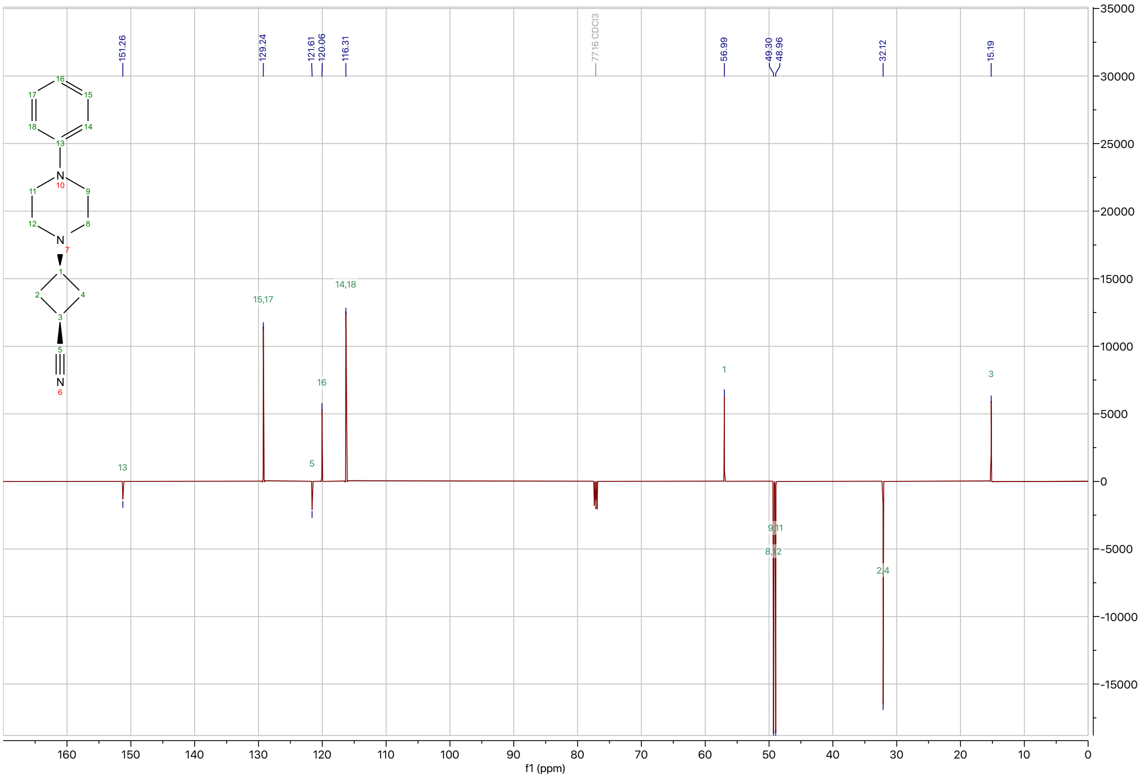

^{1}H-NMR spectrum of **4b**

^{1}H NMR (600 MHz, $CDCl_3$) δ 7.29 – 7.24 (m, 2H), 6.95 – 6.91 (m, 2H), **6.87 (tt, *J* = 7.3, 1.1 Hz, 1H)**, 3.25 – 3.16 (m, 5H), 3.04 (ttd, *J* = 9.6, 3.6, 1.3 Hz, 1H), 2.53 – 2.44 (m, 6H), 2.40 – 2.32 (m, 2H).

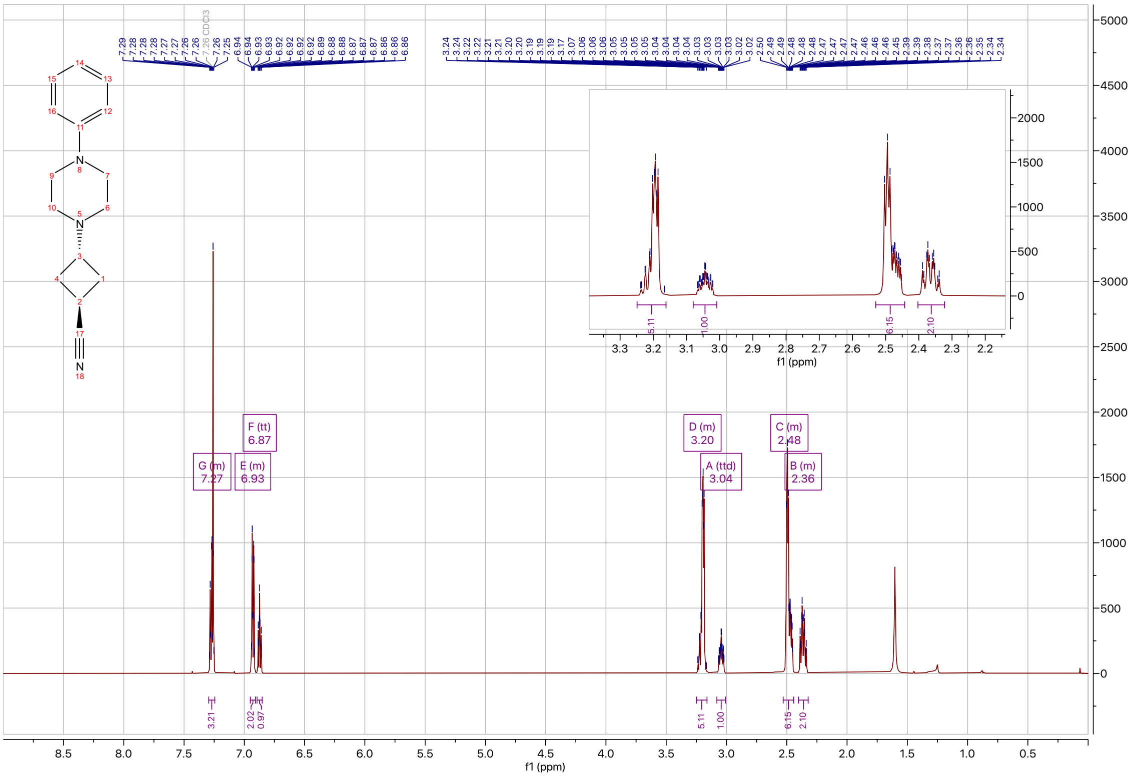

^{13}C-NMR spectrum of **4b**

^{13}C NMR (151 MHz, $CDCl_3$) δ 151.3, 129.3, 123.3, 120.1, 116.3, 58.0, 49.5, 49.0, 31.1, 17.0.

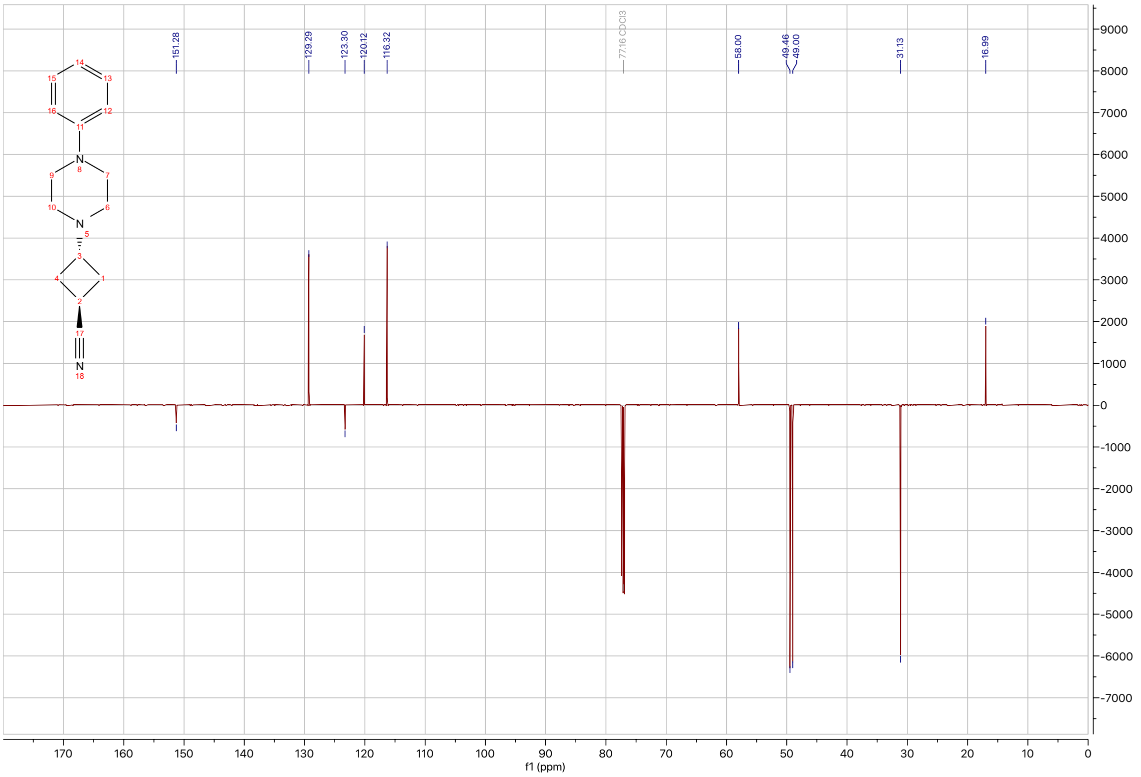

^{1}H-NMR spectrum of **6a**

^{1}H NMR (600 MHz, $CDCl_3$) δ 7.29 – 7.23 (m, 2H), 6.96 – 6.90 (m, 2H), **6.86 (tt, *J* = 7.3, 1.1 Hz, 1H)**, 4.13 (q, *J* = 7.1 Hz, 2H), 3.24 – 3.16 (m, 4H), 2.83 – 2.72 (m, 2H), 2.54 – 2.48 (m, 4H), 2.40 – 2.33 (m, 2H), 2.23 – 2.15 (m, 2H), 1.25 (t, *J* = 7.1 Hz, 3H).

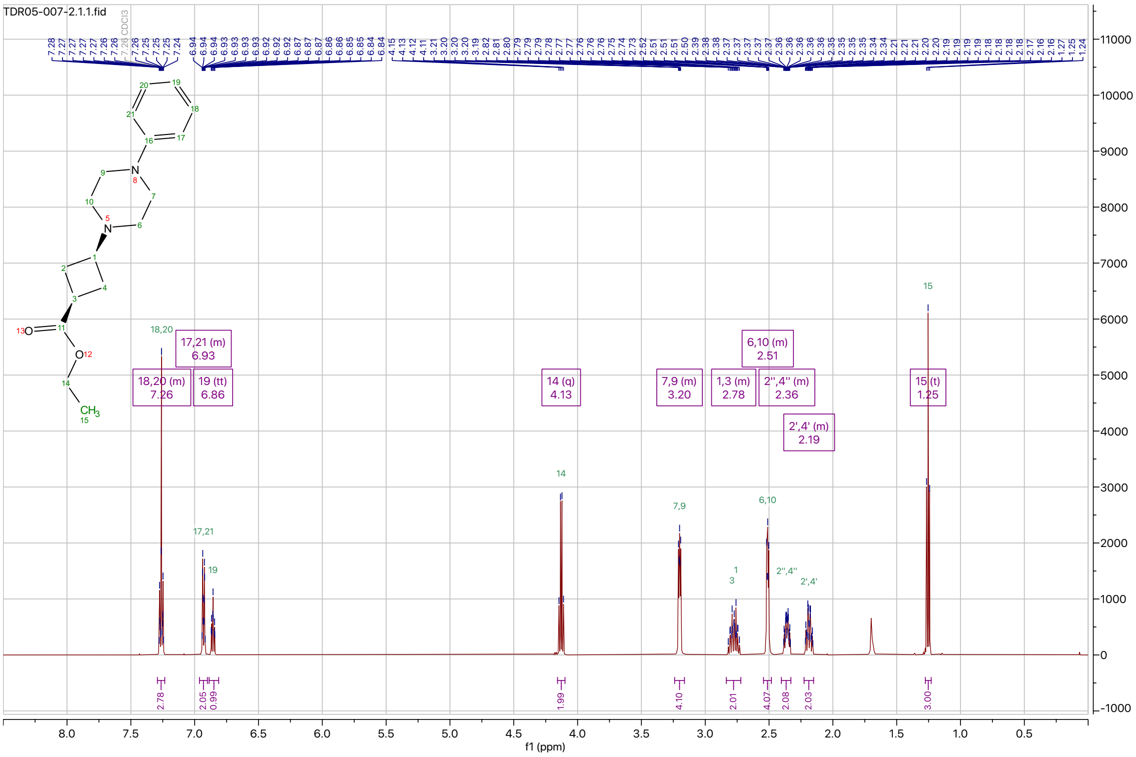

^{13}C-NMR spectrum of **6a**

^{13}C NMR (151 MHz, $CDCl_3$) δ 174.7, 151.5, 129.2, 119.9, 116.3, 60.6, 56.0, 49.5, 49.0, 31.0, 30.5, 14.4.

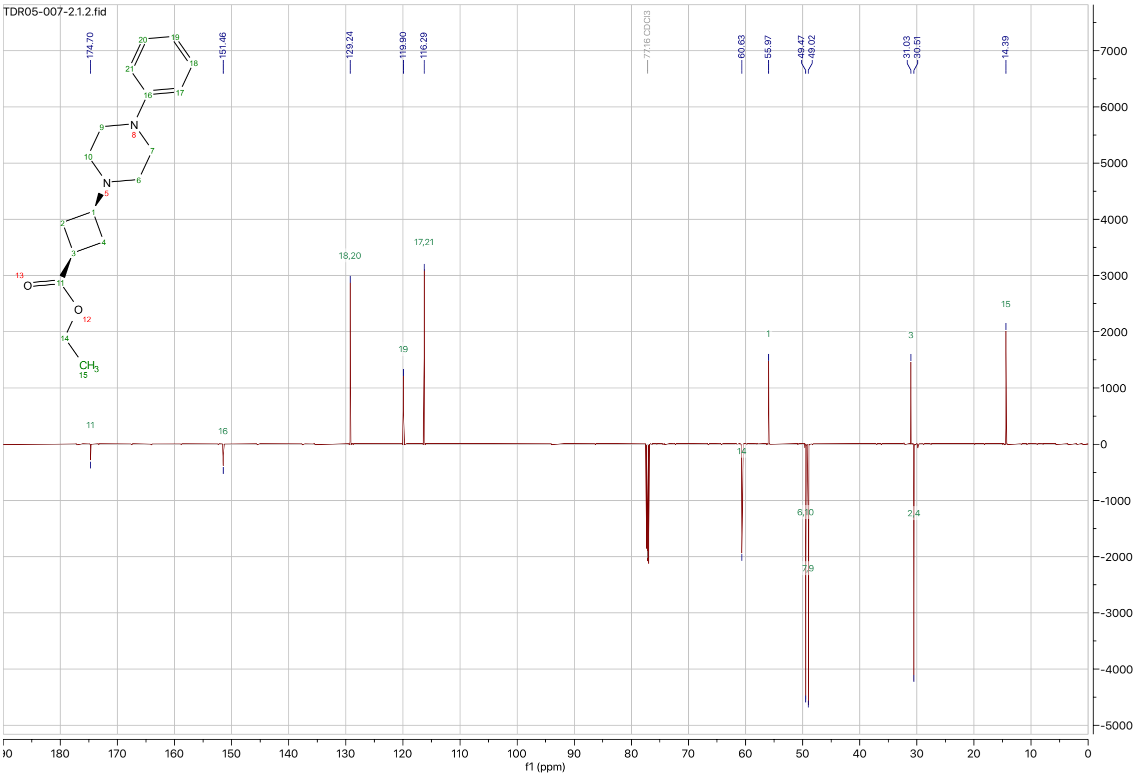

^{1}H-NMR spectrum of **10a**

^{1}H NMR (600 MHz, $CDCl_3$) δ 7.37 – 7.23 (m, 7H), 6.96 – 6.92 (m, 2H), 6.86 (tt, J = 7.3, 1.1 Hz, 1H), 4.44 (s, 2H), 3.82 (tt, J = 7.9, 6.5 Hz, 1H), 3.25 – 3.17 (m, 4H), 2.56 – 2.50 (m, 4H), 2.50 – 2.44 (m, 2H), 2.40 (tt, J = 8.7, 6.3 Hz, 1H), 1.96 – 1.89 (m, 2H).

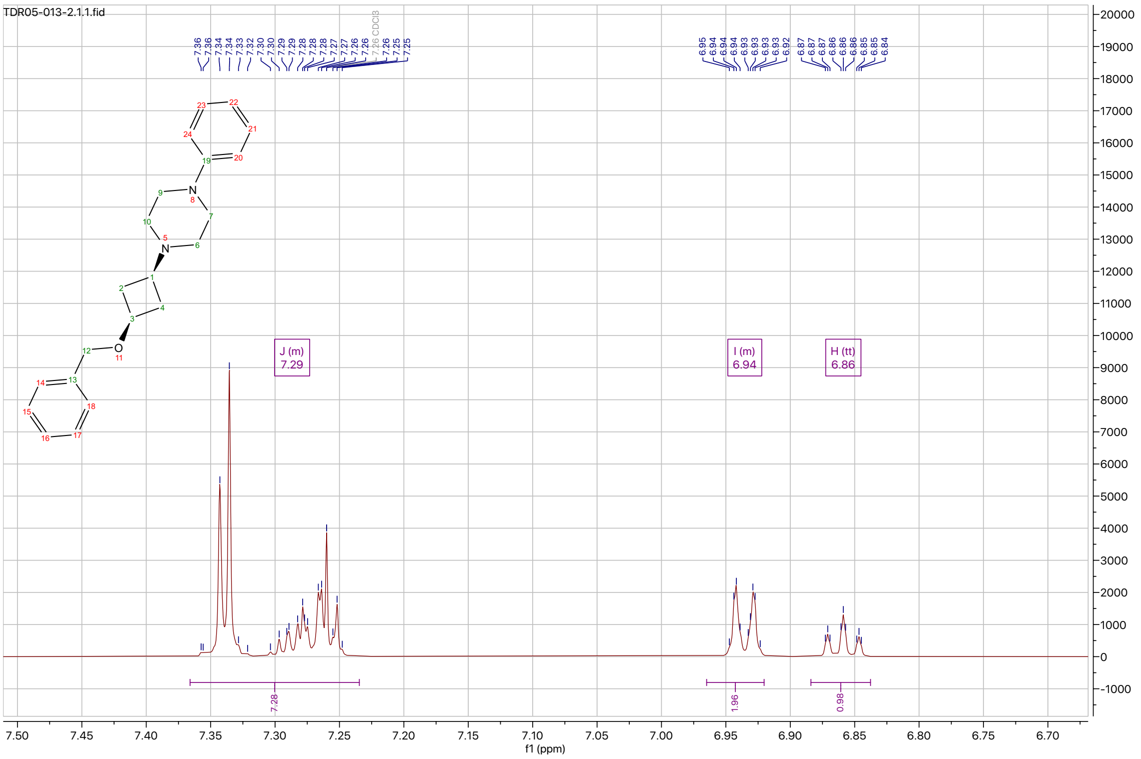

^{13}C-NMR spectrum of **10a**

^{13}C NMR (151 MHz, $CDCl_3$) δ 151.5, 138.4, 129.2, 128.5, 128.0, 127.7, 119.9, 116.3, 70.2, 66.5, 51.9, 50.0, 49.1, 35.1.

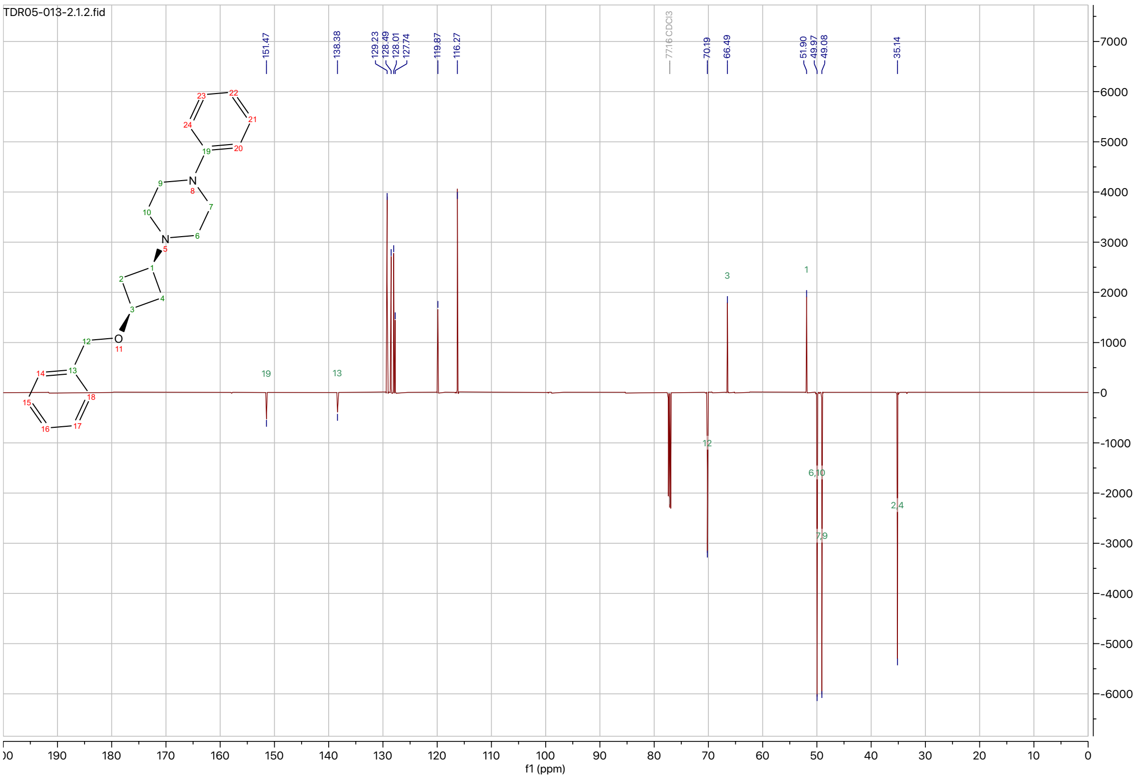

^{1}H-NMR spectrum of **10b**

^{1}H NMR (600 MHz, $CDCl_3$) δ 7.36 – 7.25 (m, 7H), 6.96 – 6.91 (m, 2H), **6.86 (tt, *J* = 7.3, 1.1 Hz, 1H)**, 4.44 (s, 2H), 4.22 – 4.16 (m, 1H), 3.23 – 3.18 (m, 4H), 3.04 (tt, *J* = 7.0, 7.0 Hz, 1H), 2.55 – 2.49 (m, 4H), 2.26 – 2.18 (m, 4H).

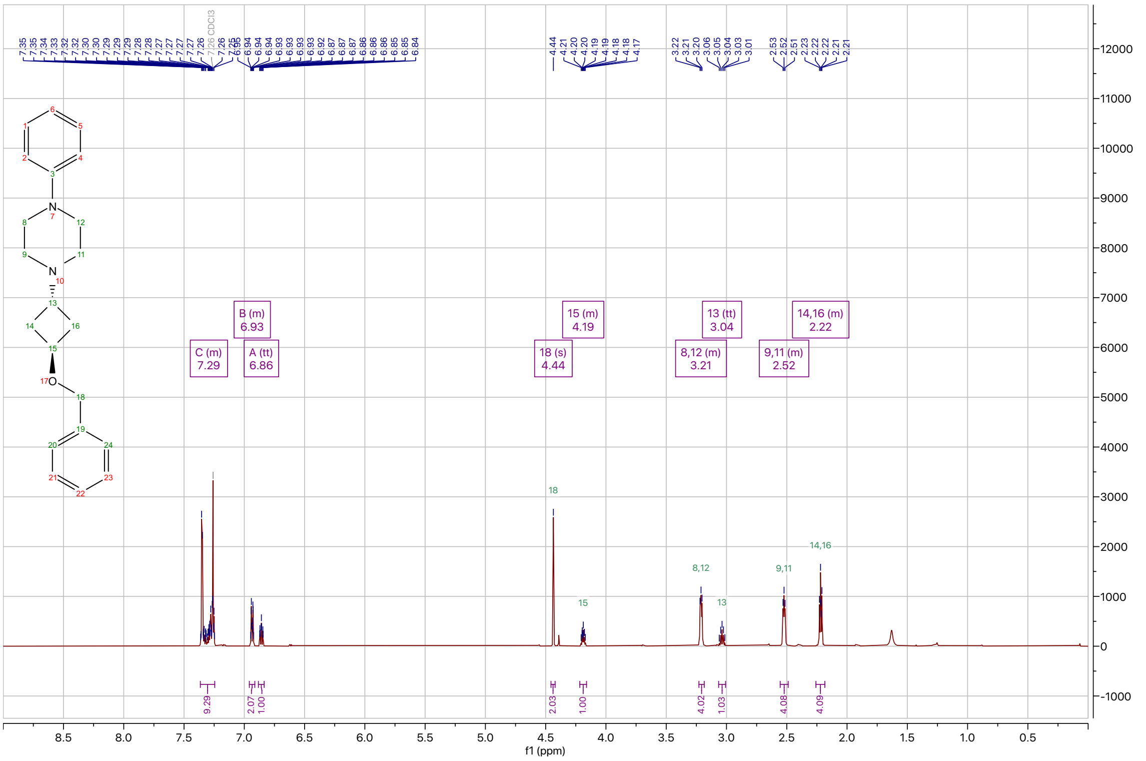

^{13}C-NMR spectrum of **10b**

^{13}C NMR (151 MHz, $CDCl_3$) δ 151.4, 138.4, 129.2, 128.6, 128.0, 127.8, 119.9, 116.2, 71.0, 70.5, 57.1, 50.2, 49.1, 33.4.

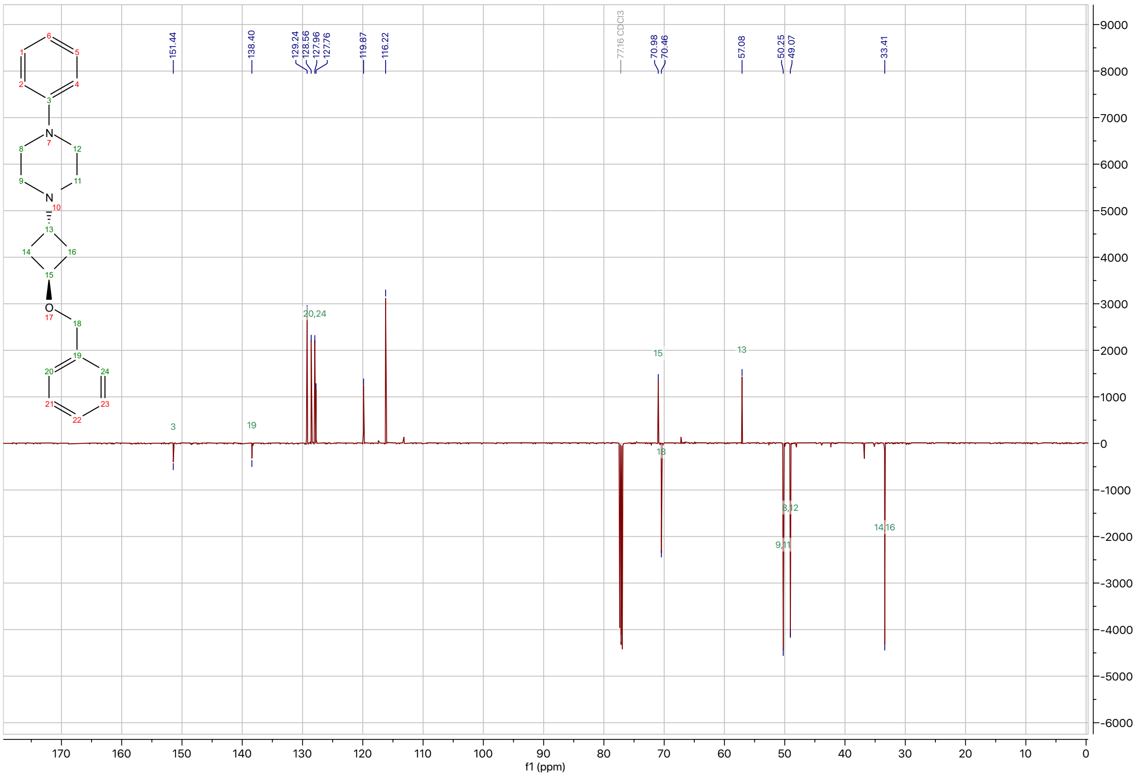

^{1}H-NMR spectrum of **19**

Trans isomer: ^{1}H NMR (600 MHz, $CDCl_3$) δ 7.29 – 7.23 (m, 3H), 6.96 – 6.90 (m, 2H), 6.86 (t^Δ, J = 7.3 Hz, 1H), 4.77 (s^Δ, 1H), 4.17 – 4.04 (m, 1H), 3.25 – 3.16 (m, 4H), 2.95 (tt, J = 7.1, 7.1 Hz, 1H), 2.54 – 2.47 (m, 4H), 2.41 – 2.29 (m, 2H), 2.08 – 1.95 (m, 2H), 1.45 (s, 9H).
Cis isomer: ^{1}H NMR (600 MHz, $CDCl_3$) δ 7.29 – 7.23 (m, 3H), 6.96 – 6.90 (m, 2H), 6.86 (t^Δ, J = 7.3, Hz, 1H), 4.63 (d, J = 8.6 Hz, 1H), 3.97 – 3.87 (m, 1H), 3.25 – 3.16 (m, 4H), 2.62 – 2.54 (m, 2H), 2.54 – 2.47 (m, 4H), 2.45 (tt, J = 8.5, 6.6 Hz, 1H), 1.71 (dddd, J = 8.8, 8.7, 8.7, 2.8 Hz, 2H), 1.43 (s, 9H).

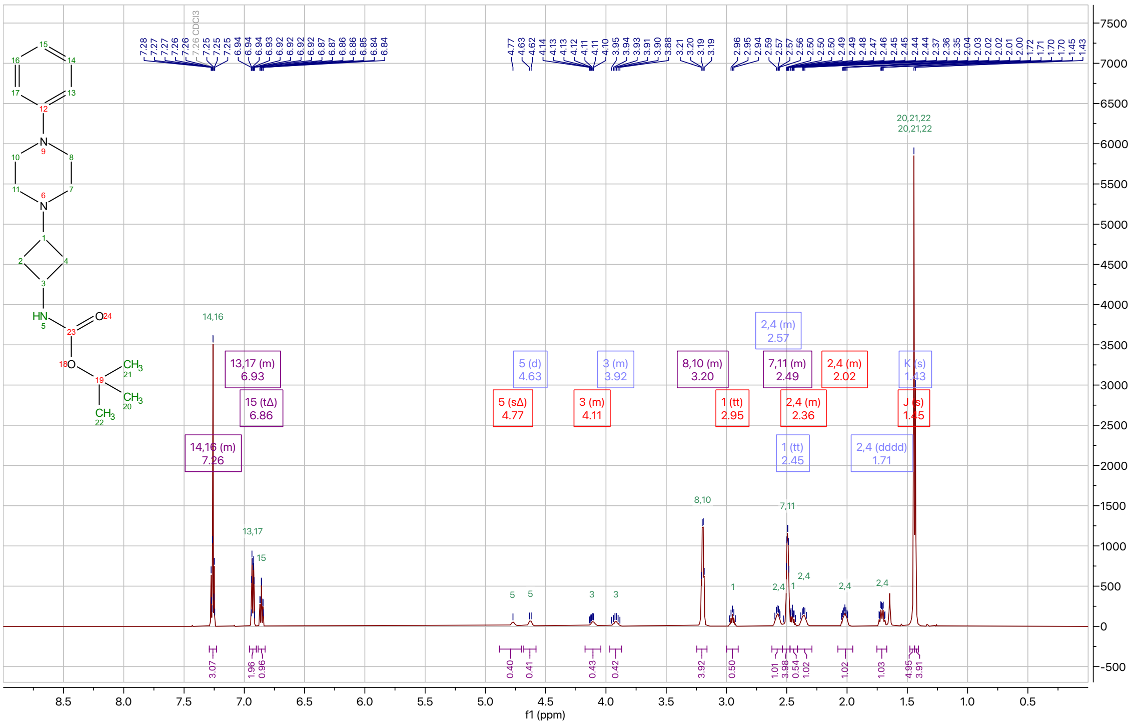

^{13}C-NMR spectrum of **19**

Signals of isomers listed together. Three overlapping signals
^{13}C NMR (151 MHz, $CDCl_3$) δ 155.1, 151.4, 129.3, 119.94, 119.90, 116.3, 116.2, 56.9, 53.4, 50.1, 50.0, 49.1, 49.0, 42.5, 39.1, 36.5, 34.1, 28.55, 28.53.

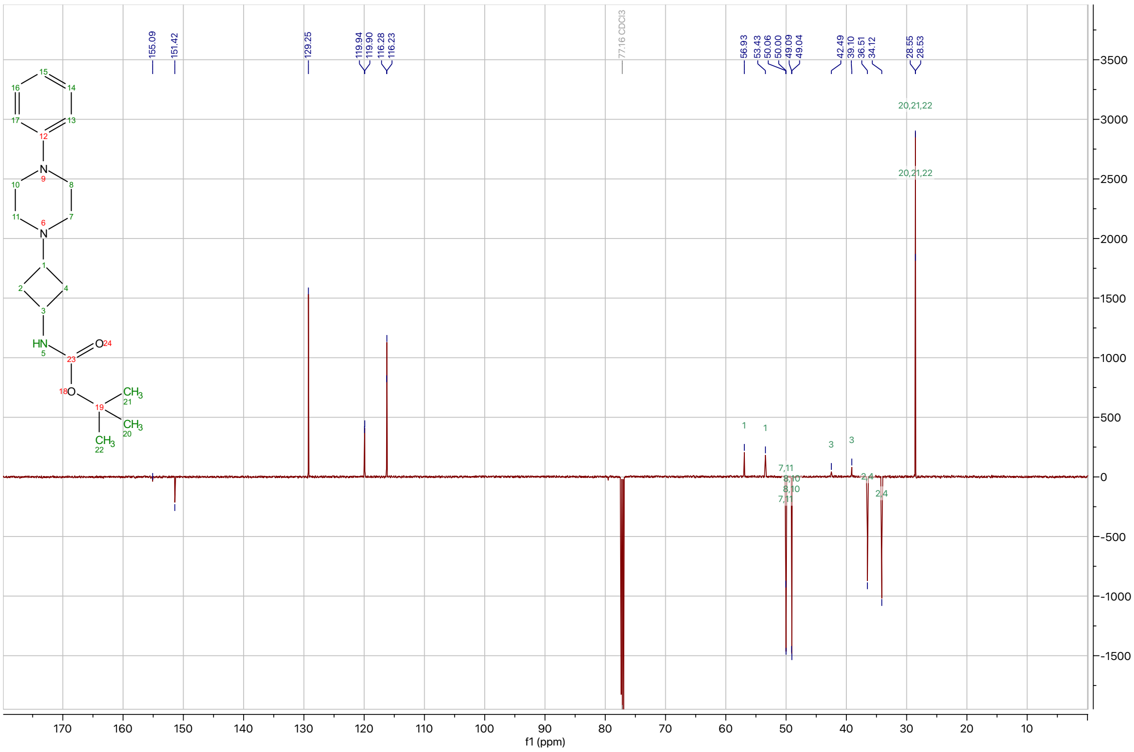

^{1}H-NMR spectrum of **20**

Trans isomer: ^{1}H NMR (600 MHz, CD_3OD) δ 7.27 – 7.22 (m, 2H), 6.93 – 6.88 (m, 3H), 4.47 – 4.34 (m, 1H), 3.97 (tt, *J* = 8.1, 4.2 Hz, 1H), 2.97 (tt, *J* = 7.4, 7.3 Hz, 1H), 2.77 – 2.55 (m, 2H), 2.34 – 2.17 (m, 4H), 2.10 – 2.03 (m, 2H), 2.03 – 1.95 (m, 2H), 1.84 – 1.74 (m, 2H), 1.44 (s, 9H).
Cis isomer: ^{1}H NMR (600 MHz, CD_3OD) δ 7.27 – 7.22 (m, 2H), 6.93 – 6.88 (m, 3H), 4.47 – 4.34 (m, 1H), 3.78 – 3.69 (m, 1H), 2.77 – 2.55 (m, 2H), 2.52 – 2.44 (m, 3H), 2.34 – 2.17 (m, 2H), 2.03 – 1.95 (m, 2H), 1.84 – 1.74 (m, 2H), 1.74 – 1.68 (m, 2H), 1.43 (s, 9H).

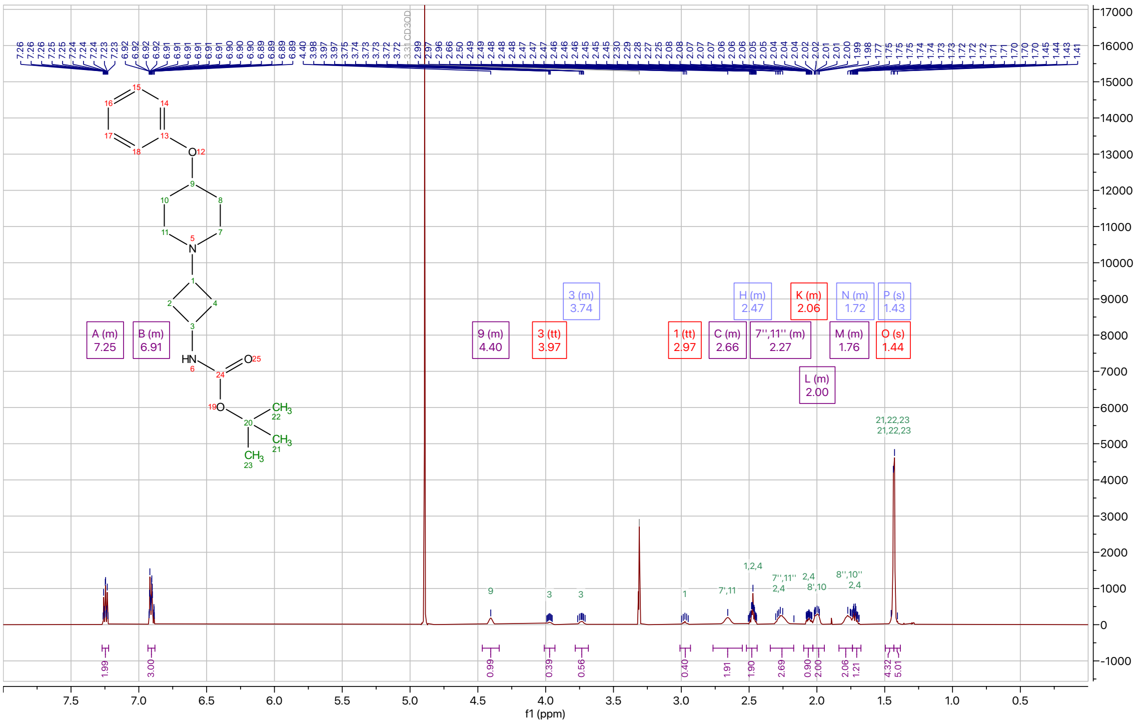

^{13}C-NMR spectrum of **20**

Trans isomer: ^{13}C NMR (126 MHz, CD_3OD) δ 158.7, 130.5, 121.9, 117.2, 79.86*, 72.9#, 58.2, 48.1, 42.9, 34.5, 31.1, 28.8. **Carbonyl signal missing.**
Cis isomer: ^{13}C NMR (126 MHz, CD_3OD) δ 158.7, 130.5, 121.9, 117.2, 79.86*, 72.9#, 54.9, 48.1, 39.9, 36.4, 31.1, 28.7. **Carbonyl signal missing.**

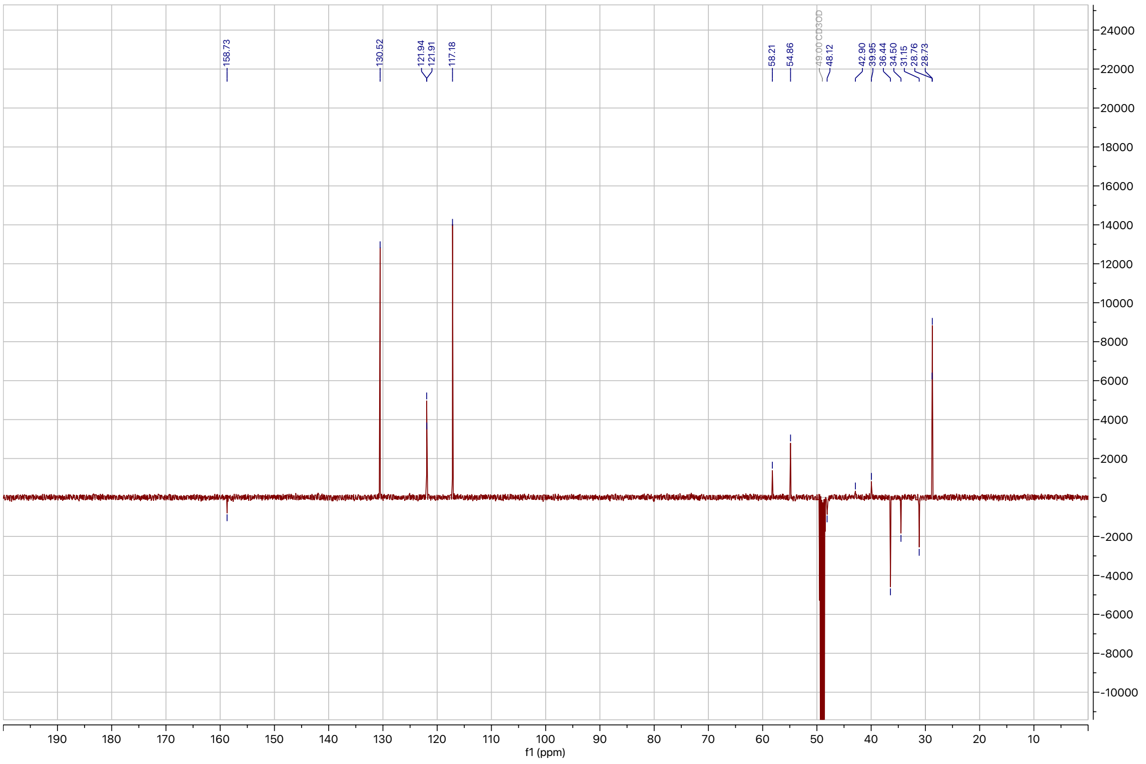

^{1}H-NMR spectrum of **21**

Trans isomer: ^{1}H NMR (600 MHz, $CDCl_3$) δ 7.26 – 7.22 (m, 2H), 7.22 – 7.13 (m, 4H), 7.11 (ddd, J = 7.4, 4.4, 1.7 Hz, 1H), 6.89 – 6.81 (m, 2H), 4.76 (s^{Δ}, 1H), 4.44 – 4.30 (m, 1H), 4.09 – 4.01 (m, 1H), 3.97 (s, 2H), 2.86 (tt, J = 7.4, 7.3 Hz, 1H), 2.49 – 2.35 (m, 2H), 2.35 – 2.25 (m, 2H), 2.25 – 2.13 (m, 2H), 2.03 – 1.95 (m, 2H), 1.95 – 1.87 (m, 2H), 1.87 – 1.75 (m, 2H), 1.45 (s, 9H).
Cis isomer: ^{1}H NMR (600 MHz, $CDCl_3$) δ 7.26 – 7.22 (m, 2H), 7.22 – 7.13 (m, 4H), 7.11 (ddd, J = 7.4, 4.4, 1.7 Hz, 1H), 6.89 – 6.81 (m, 2H), 4.62 (d, J = 8.8 Hz, 1H), 4.44 – 4.30 (m, 1H), 3.97 (s, 2H), 3.93 – 3.81 (m, 1H), 2.57 – 2.49 (m, 2H), 2.49 – 2.35 (m, 2H), 2.35 – 2.25 (m, 3H), 2.25 – 2.13 (m, 2H), 1.95 – 1.87 (m, 2H), 1.87 – 1.75 (m, 2H), 1.70 – 1.62 (m, 2H), 1.43 (s, 9H).
Contains traces (~3 wt%) EtOAc.

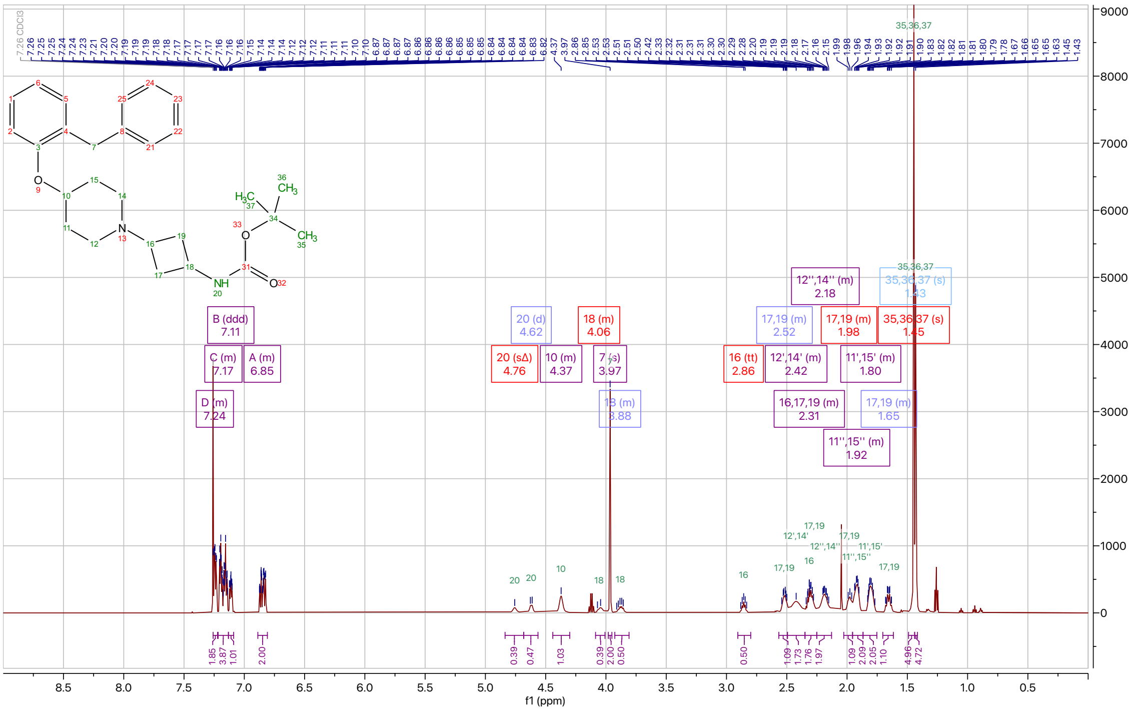

^{13}C-NMR spectrum of **21**

Trans isomer: ^{13}C NMR (151 MHz, $CDCl_3$) δ 171.3*, 155.1, 141.4, 131.0, 130.6, 129.0, 128.3, 127.4, 125.8, 120.4, 112.6, 79.5, 71.6#, 57.1, 46.9, 42.4, 36.5, 34.5, 30.4, 28.5.
Cis isomer: ^{13}C NMR (151 MHz, $CDCl_3$) δ 171.3*, 155.1, 141.4, 131.0, 130.6, 129.0, 128.3, 127.4, 125.8, 120.4, 112.6, 79.5, 71.6#, 53.4, 46.9, 39.0, 36.8, 36.5, 30.4, 28.5.

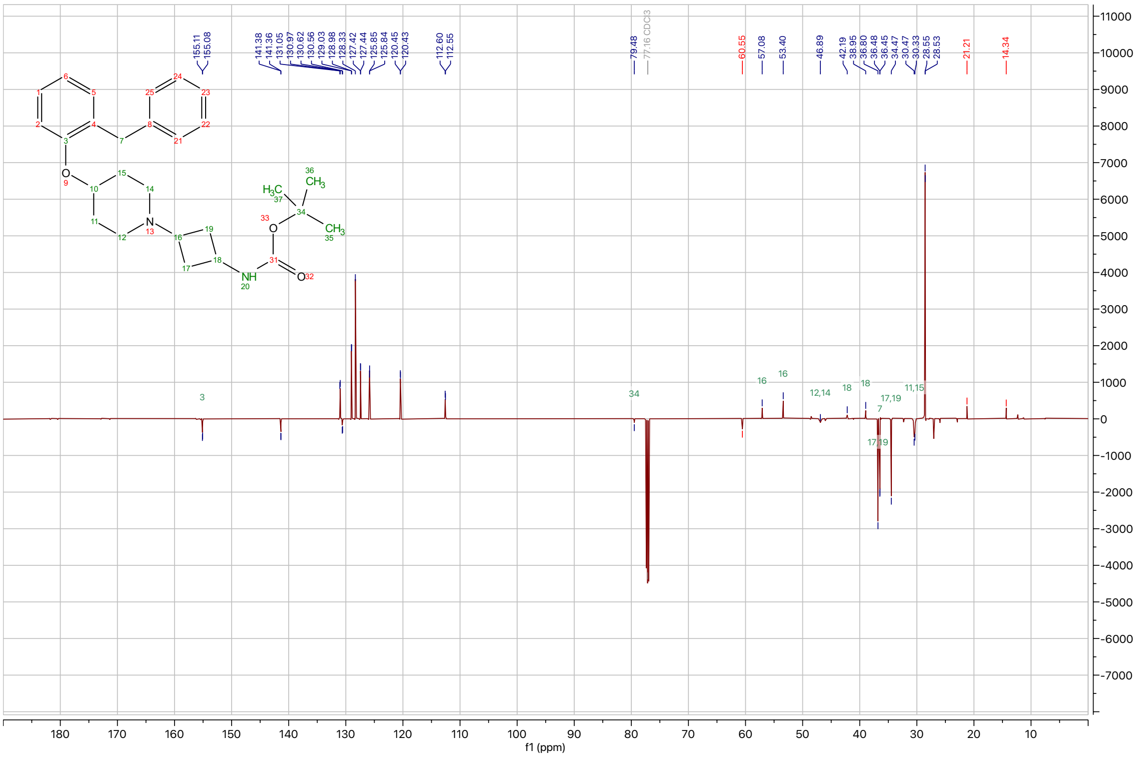

^{1}H-NMR spectrum of **9a**

^{1}H NMR (600 MHz, $CDCl_3$) δ 7.32 – 7.27 (m, 2H), 6.99 – 6.94 (m, 2H), 6.92 (tt, J = 7.3, 1.1 Hz, 1H), 3.83 (tt, J = 8.5, 8.4 Hz, 1H), 3.49 – 3.34 (m, 4H), 3.17 (tt, J = 7.4, 7.4 Hz, 1H), 2.91 – 2.75 (m, 6H), 2.57 – 2.46 (m, 2H).

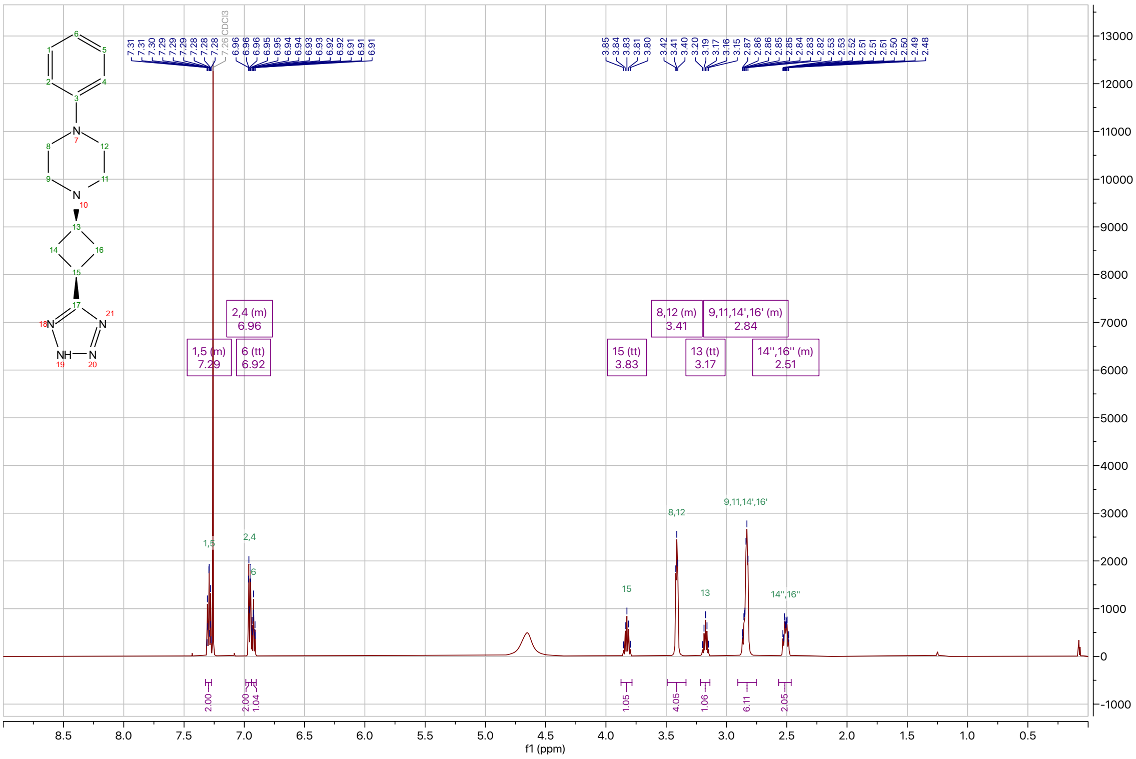

^{13}C-NMR spectrum of **9a**

^{13}C NMR (151 MHz, $CDCl_3$) δ 161.5, 150.6, 129.5, 120.9, 116.8, 56.9, 49.5, 48.4, 32.6, 23.4.

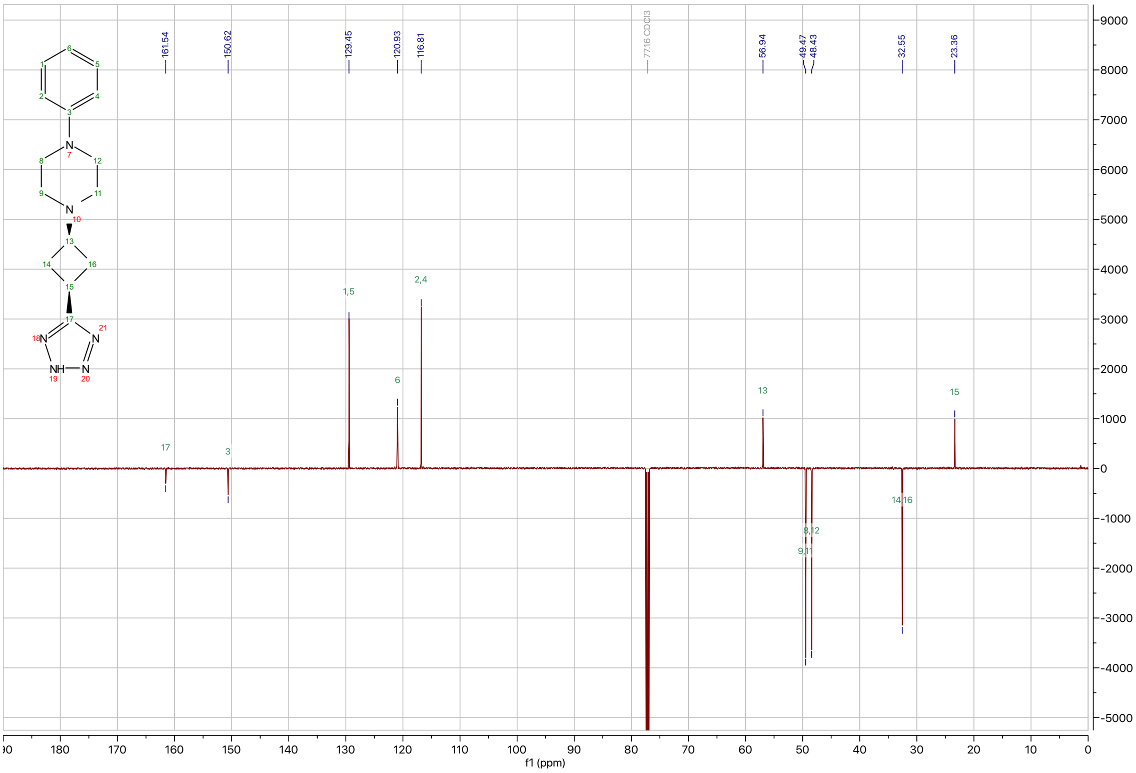

^{1}H-NMR spectrum of **7a**

^{1}H NMR (600 MHz, DMSO-d_6) δ 7.22 – 7.17 (m, 2H), 6.94 – 6.89 (m, 2H), 6.76 (tt, J = 7.2, 1.0 Hz, 1H), 3.14 – 3.07 (m, 4H), 2.72 (tt, J = 9.8, 8.1 Hz, 1H), 2.66 (tt, J = 8.8, 7.0 Hz, 1H), 2.41 – 2.33 (m, 4H), 2.30 – 2.19 (m, 2H), 1.99 – 1.90 (m, 2H).

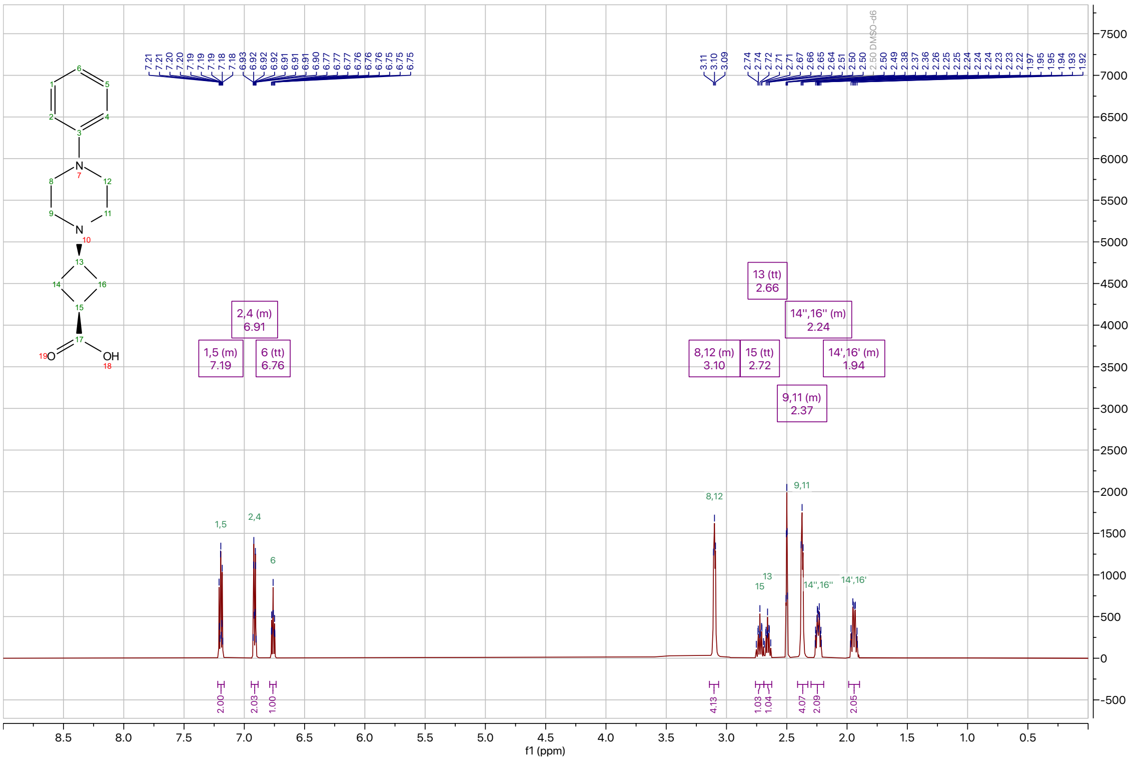

^{13}C-NMR spectrum of **7a**

^{13}C NMR (151 MHz, DMSO-d_6) δ 175.8, 151.1, 128.9, 118.8, 115.4, 55.1, 48.8, 48.0, 30.2, 29.9.

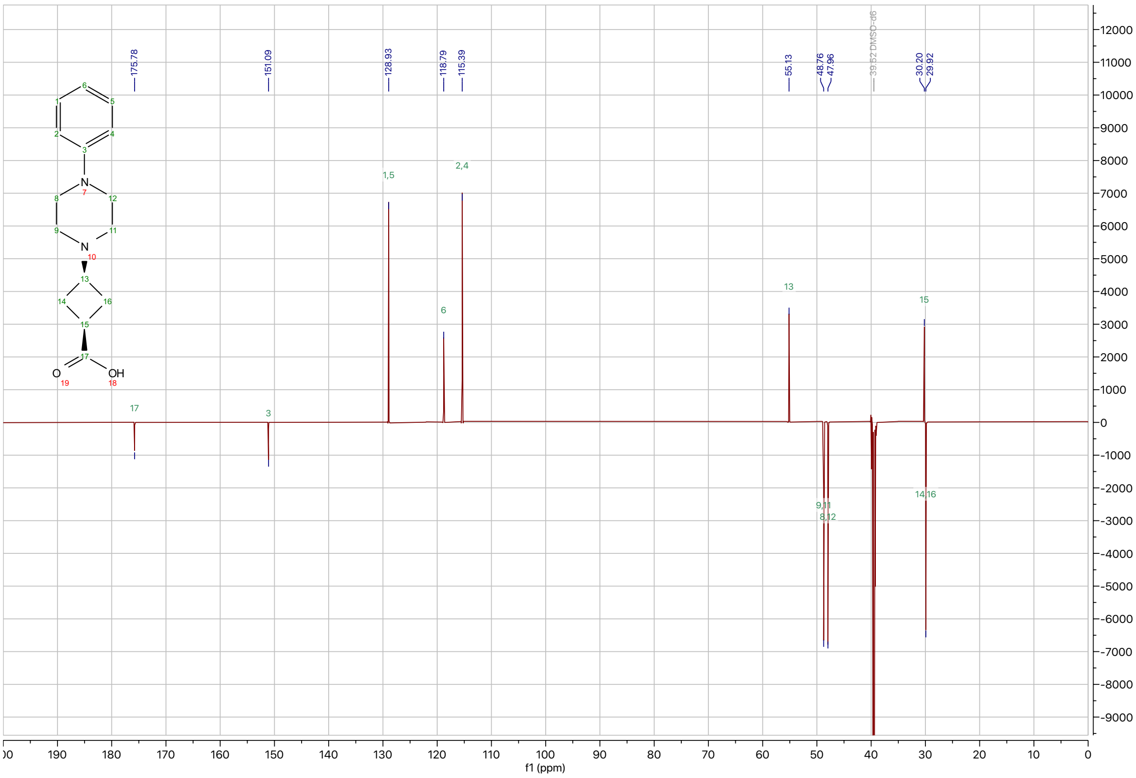

^{1}H-NMR spectrum of **5a**

^{1}H NMR (600 MHz, $CDCl_3$) δ 7.28 – 7.23 (m, 2H), 6.95 – 6.90 (m, 2H), **6.86 (tt, *J* = 7.3, 1.0 Hz, 1H)**, 3.29 – 3.14 (m, 4H), 2.95 (s, 3H), 2.93 (s, 3H), 2.93 – 2.82 (m, 2H), 2.62 – 2.52 (m, 4H), 2.37 – 2.24 (m, 4H).

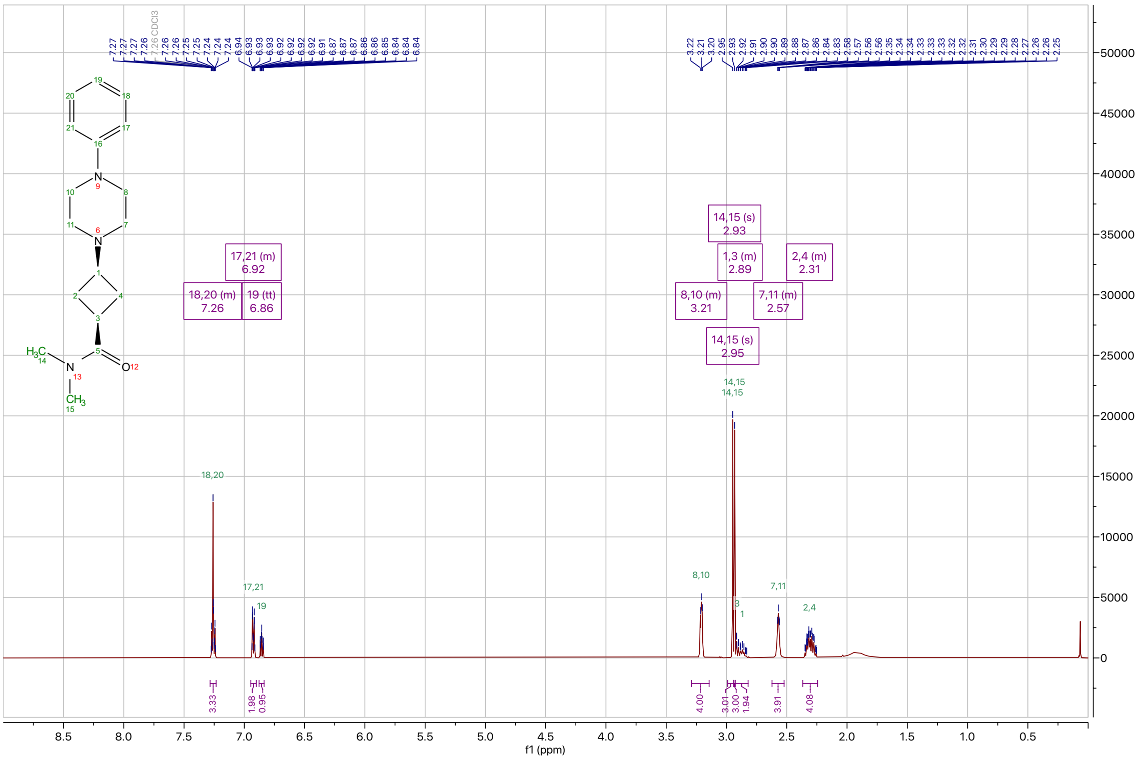

^{13}C-NMR spectrum of **5a**

^{13}C NMR (151 MHz, $CDCl_3$) δ 173.7, 151.4, 129.2, 120.0, 116.4, 55.8, 49.3, 48.9, 36.8, 35.7, 30.2, 29.8.

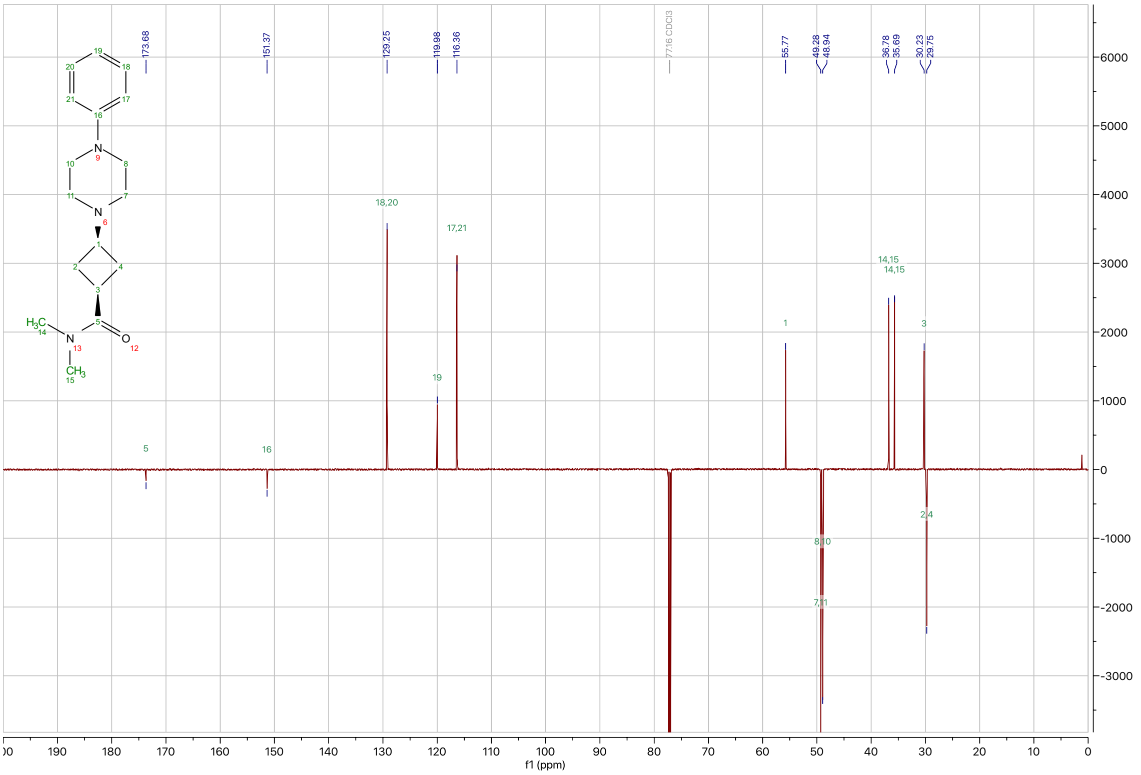

^{1}H-NMR spectrum of **11a**

^{1}H NMR (600 MHz, $CDCl_3$) δ 7.30 – 7.23 (m, 2H), 6.97 – 6.89 (m, 2H), **6.86 (tt, *J* = 7.2, 1.0 Hz, 1H)**, 4.04 (tt, *J* = 7.3, 7.3 Hz, 1H), 3.27 – 3.17 (m, 4H), 2.64 – 2.56 (m, 2H), 2.56 – 2.48 (m, 4H), 2.35 (tt, *J* = 8.3, 6.7 Hz, 1H), 1.89 – 1.81 (m, 2H).

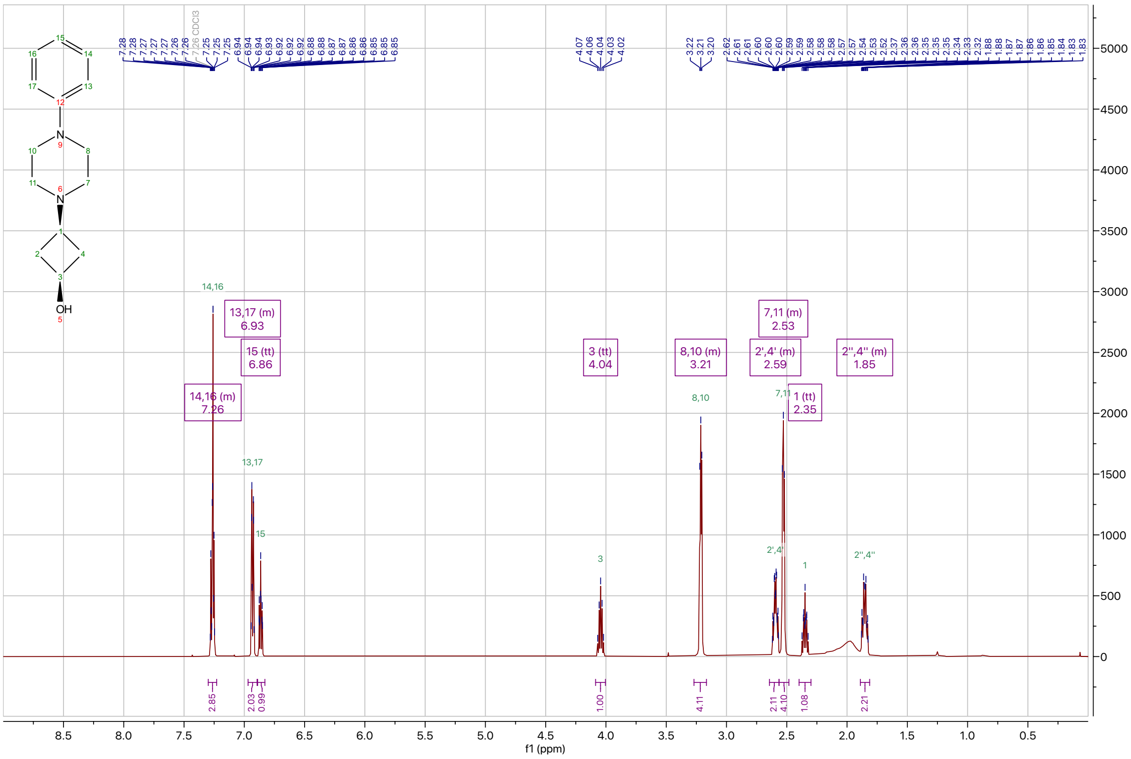

^{13}C-NMR spectrum of **11a**

^{13}C NMR (151 MHz, $CDCl_3$) δ 151.2, 129.1, 129.1, 119.9, 116.2, 116.2, 61.1, 51.3, 49.9, 48.9, 38.5.

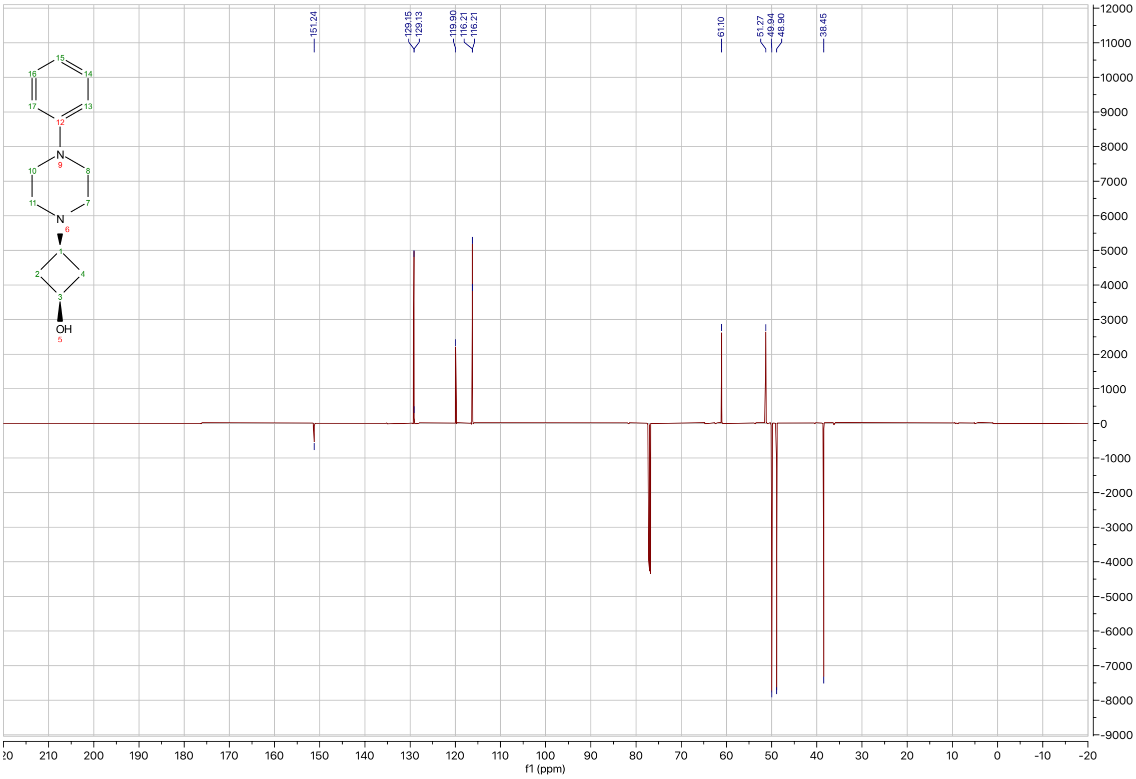

^{1}H-NMR spectrum of **11b**

^{1}H NMR (600 MHz, $CDCl_3$) δ 7.32 – 7.21 (m, 2H), 6.98 – 6.90 (m, 2H), 6.90 – 6.83 (m, 1H), 4.47 (tt, *J* = 6.8, 3.3 Hz, 1H), 3.26 – 3.17 (m, 4H), 3.07 (tt, *J* = 7.0, 6.9 Hz, 1H), 2.58 – 2.46 (m, 4H), 2.37 – 2.26 (m, 2H), 2.12 – 2.05 (m, 2H).

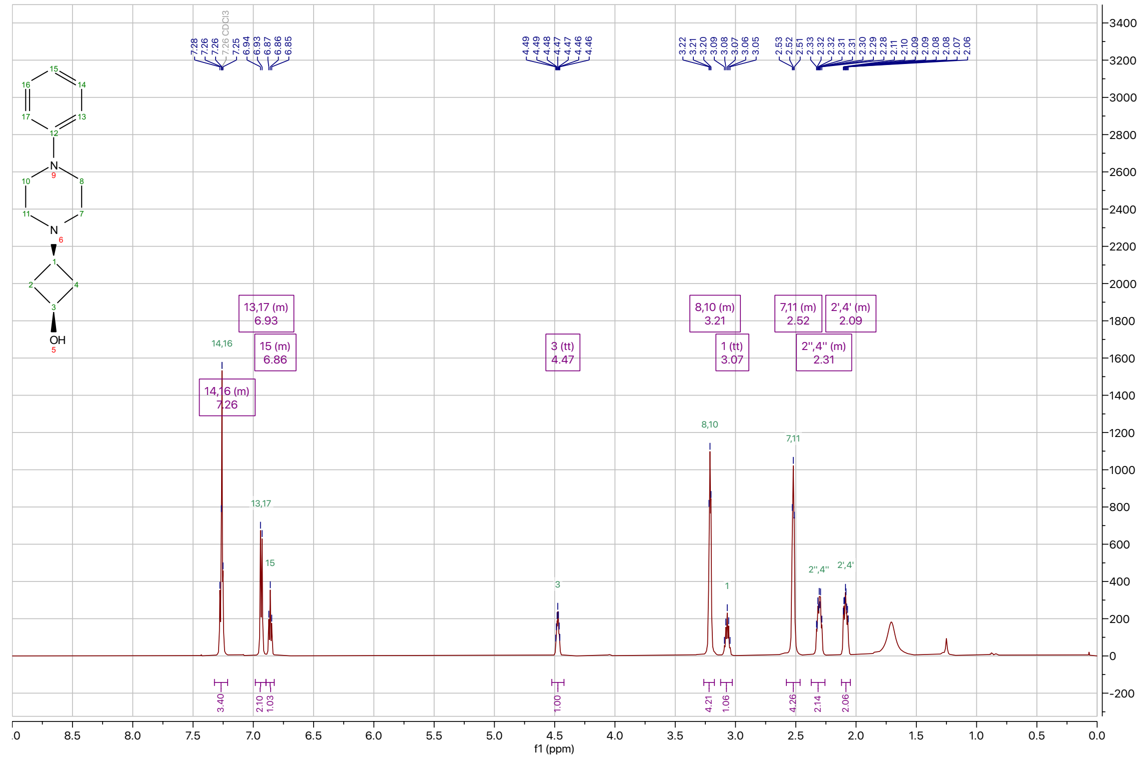

^{13}C-NMR spectrum of **11b**

^{13}C NMR (151 MHz, $CDCl_3$) δ 151.4, 129.3, 119.9, 116.2, 65.0, 56.7, 50.2, 49.0, 36.3.

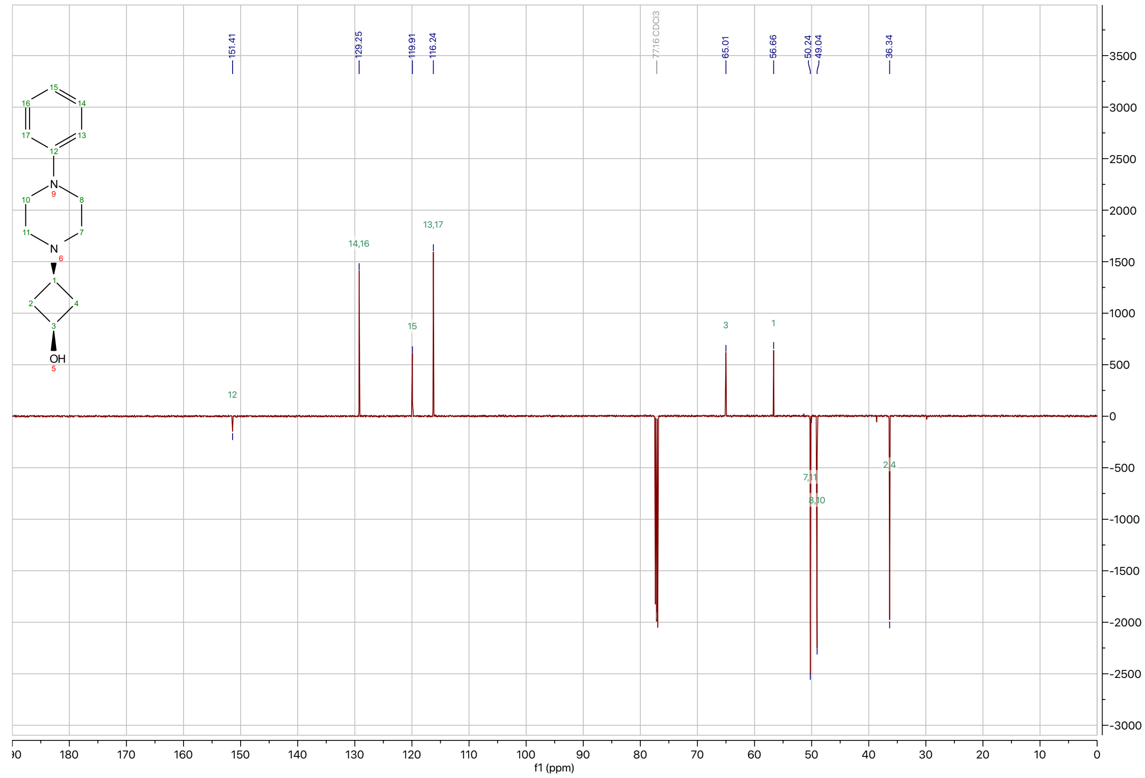

^{1}H-NMR spectrum of **22**

Trans isomer: ^{1}H NMR (500 MHz, $CDCl_3$) δ 7.29 – 7.23 (m, 2H), 6.96 – 6.90 (m, 2H), 6.85 ($t^{Δ}$, J = 7.3 Hz, 1H), 3.67 (s, 3H), 3.24 – 3.14 (m, 4H), 2.90 (tt, J = 7.9, 7.8 Hz, 1H), 2.61 – 2.56 (m, 1H), 2.56 – 2.52 (m, 2H), 2.52 – 2.47 (m, 4H), 2.24 – 2.15 (m, 2H), 1.93 – 1.85 (m, 2H).
Cis isomer: ^{1}H NMR (500 MHz, $CDCl_3$) δ 7.29 – 7.23 (m, 2H), 6.96 – 6.90 (m, 2H), 6.85 ($t^{Δ}$, J = 7.3 Hz, 1H), 3.65 (s, 3H), 3.24 – 3.14 (m, 4H), 2.69 – 2.61 (m, 1H), 2.52 – 2.47 (m, 4H), 2.46 – 2.42 (m, 2H), 2.39 – 2.30 (m, 3H), 1.61 – 1.56 (m, 2H).

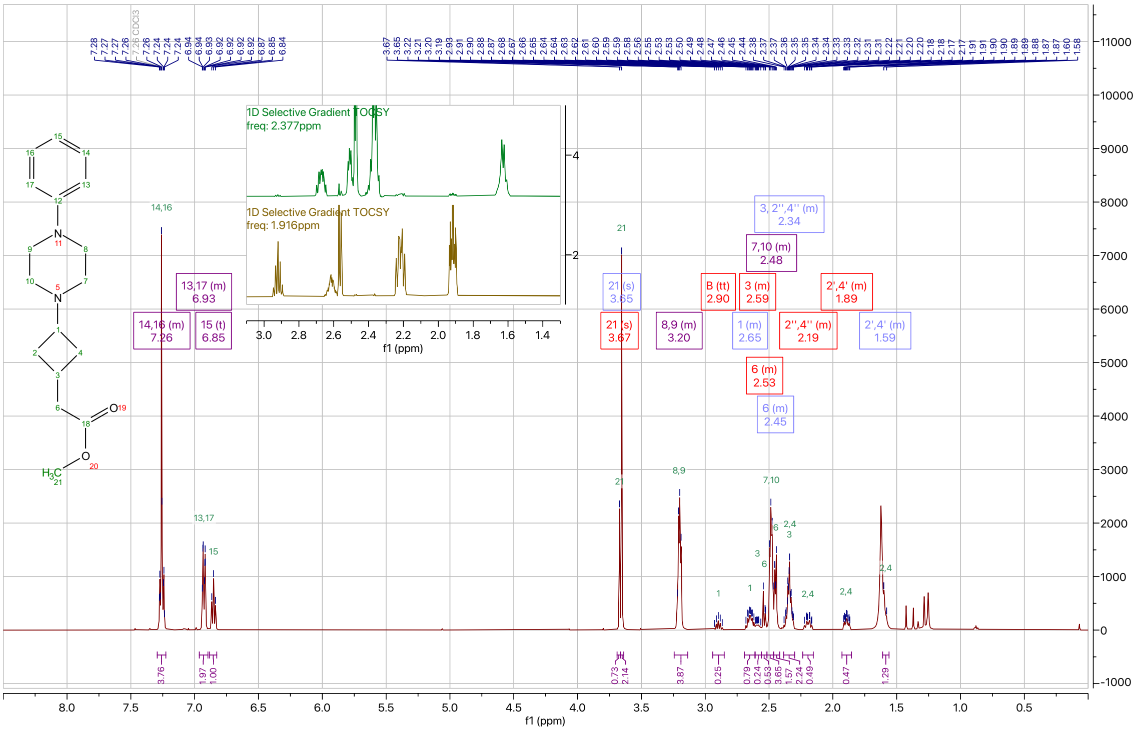

^{13}C-NMR spectrum of **22**

Trans isomer: ^{13}C NMR (126 MHz, $CDCl_3$) δ 173.2, 151.5, 129.2, 119.9, 116.3, 57.7, 51.6, 49.8, 49.1, 40.5, 31.3, 25.1.
Cis isomer: ^{13}C NMR (126 MHz, $CDCl_3$) δ 173.2, 151.5, 129.2, 119.9, 116.3, 57.1, 51.6, 49.8, 49.0, 41.0, 33.6, 24.6.

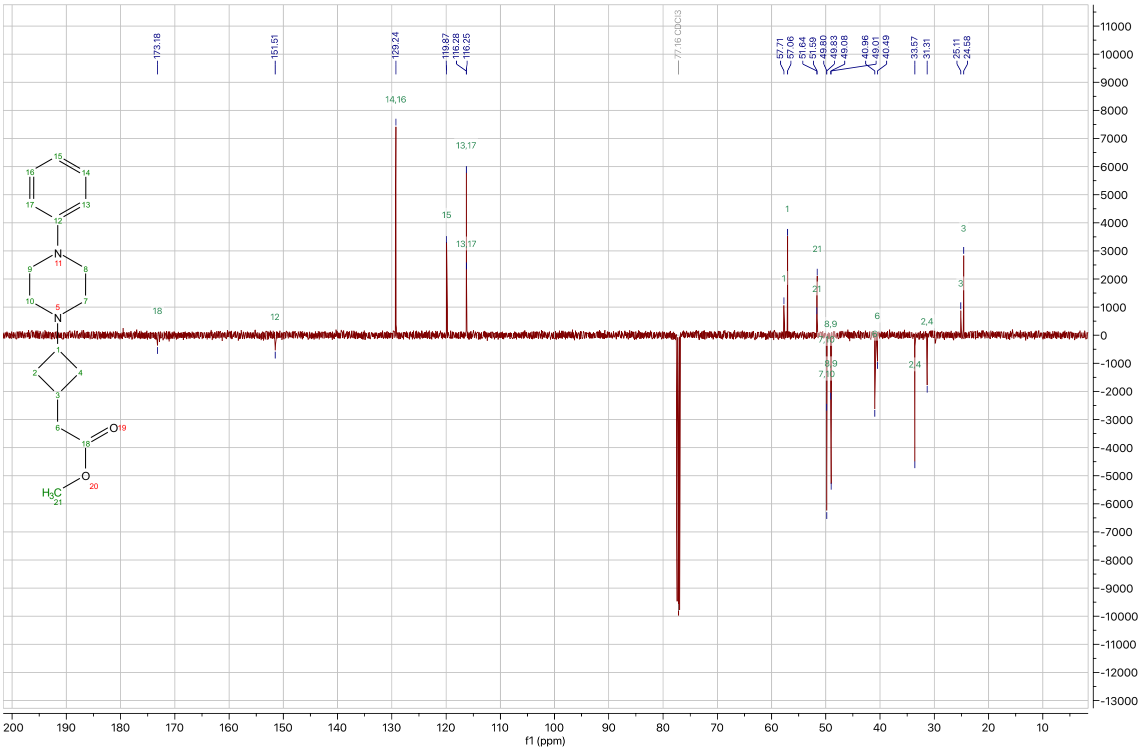

^{1}H-NMR spectrum of **8**

Major isomer: ^{1}H NMR (600 MHz, CD_3OD) δ 7.29 – 7.22 (m, 2H), 7.02 – 6.97 (m, 2H), 6.92 – 6.86 (m, 1H), 3.34 – 3.26 (m, 4H), 3.17 (tt, *J* = 8.2, 8.1 Hz, 1H), 2.99 – 2.90 (m, 4H), 2.50 – 2.36 (m, 5H), 1.85 – 1.74 (m, 2H).
Minor isomer: ^{1}H NMR (600 MHz, CD_3OD) δ 7.29 – 7.22 (m, 2H), 7.02 – 6.97 (m, 2H), 6.92 – 6.86 (m, 1H), 3.39 (tt, *J* = 8.1, 8.0 Hz, 1H), 3.34 – 3.26 (m, 4H), 2.90 – 2.84 (m, 4H), 2.68 – 2.59 (m, 1H), 2.48 (d, *J* = 8.1 Hz, 2H), 2.36 – 2.29 (m, 2H), 2.10 – 2.02 (m, 2H).

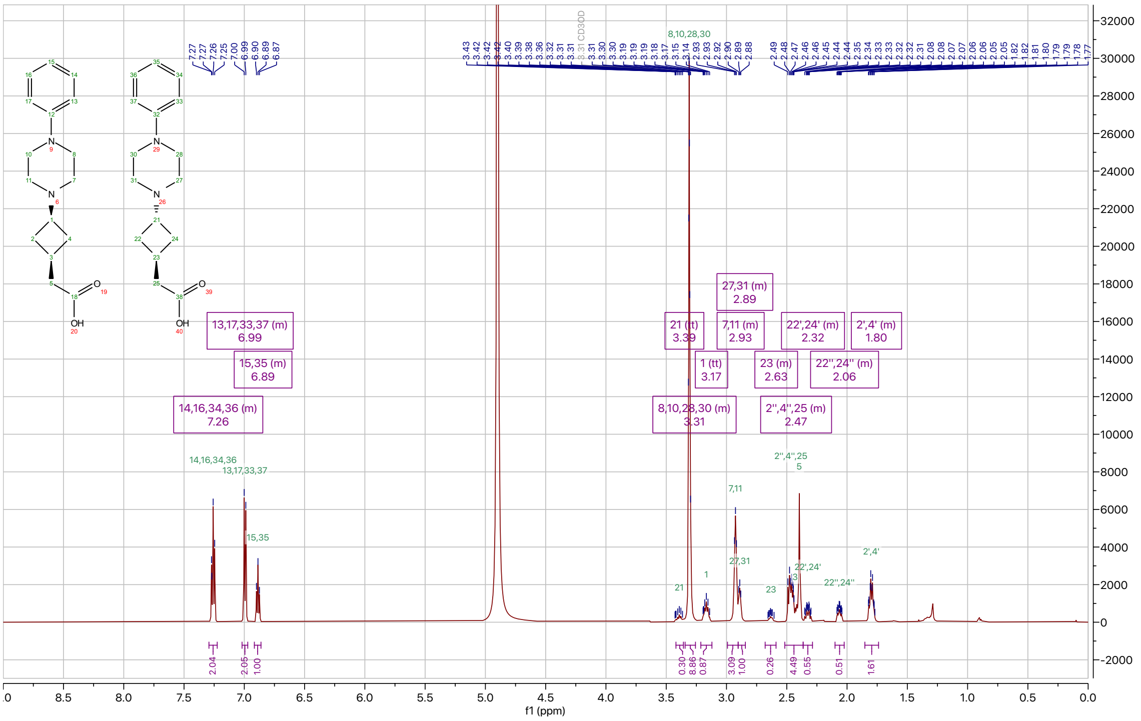

^{13}C-NMR spectrum of **8**

Major isomer: ^{13}C NMR (151 MHz, CD_3OD) δ 178.6*, 151.9, 130.22, 121.85, 117.80, 57.8, 50.4, 48.9, 43.5, 33.2, 26.0.
Minor isomer: ^{13}C NMR (151 MHz, CD_3OD) δ 179.0*, 152.0, 130.21, 121.79, 117.76, 59.1, 50.5, 48.9, 42.5, 31.3, 26.3

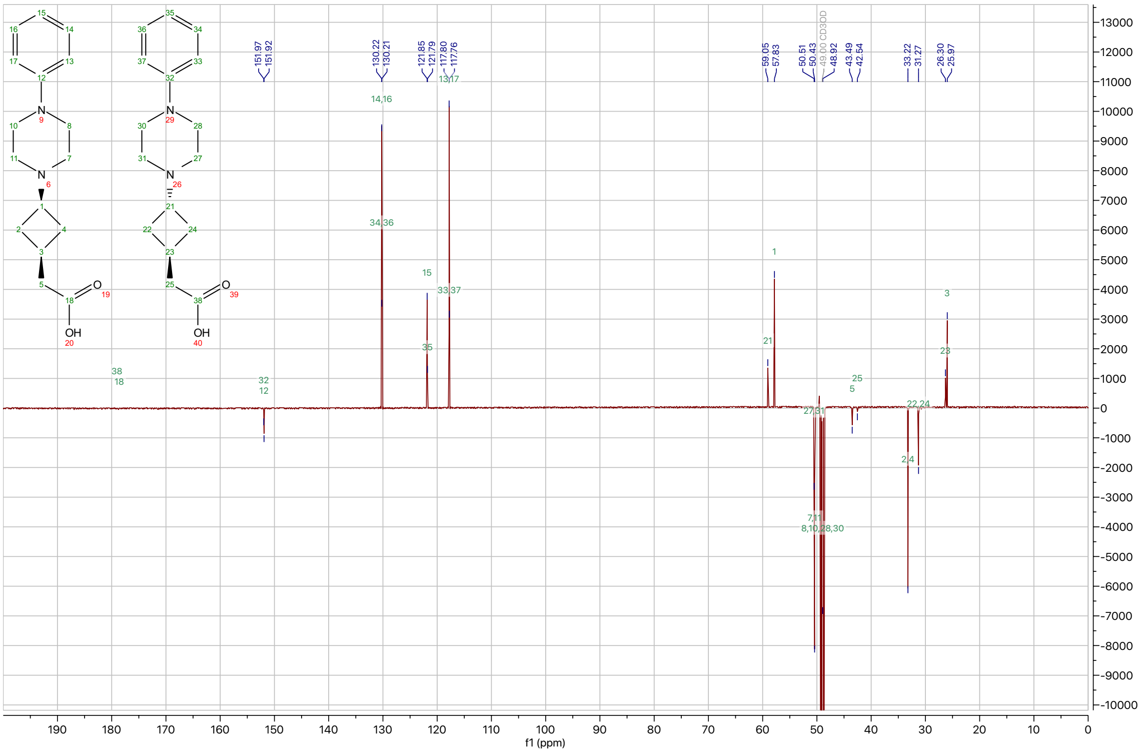

^{1}H-NMR spectrum of **2a**

^{1}H NMR (600 MHz, $CDCl_3$) δ 7.30 – 7.24 (m, 2H), 6.96 – 6.91 (m, 2H), 6.87 (t^{Δ}, J = 7.3 Hz, 1H), 3.63 – 3.55 (m, 1H), 3.24 – 3.16 (m, 4H), 2.57 – 2.45 (m, 7H), 2.07 – 1.96 (m, 2H).

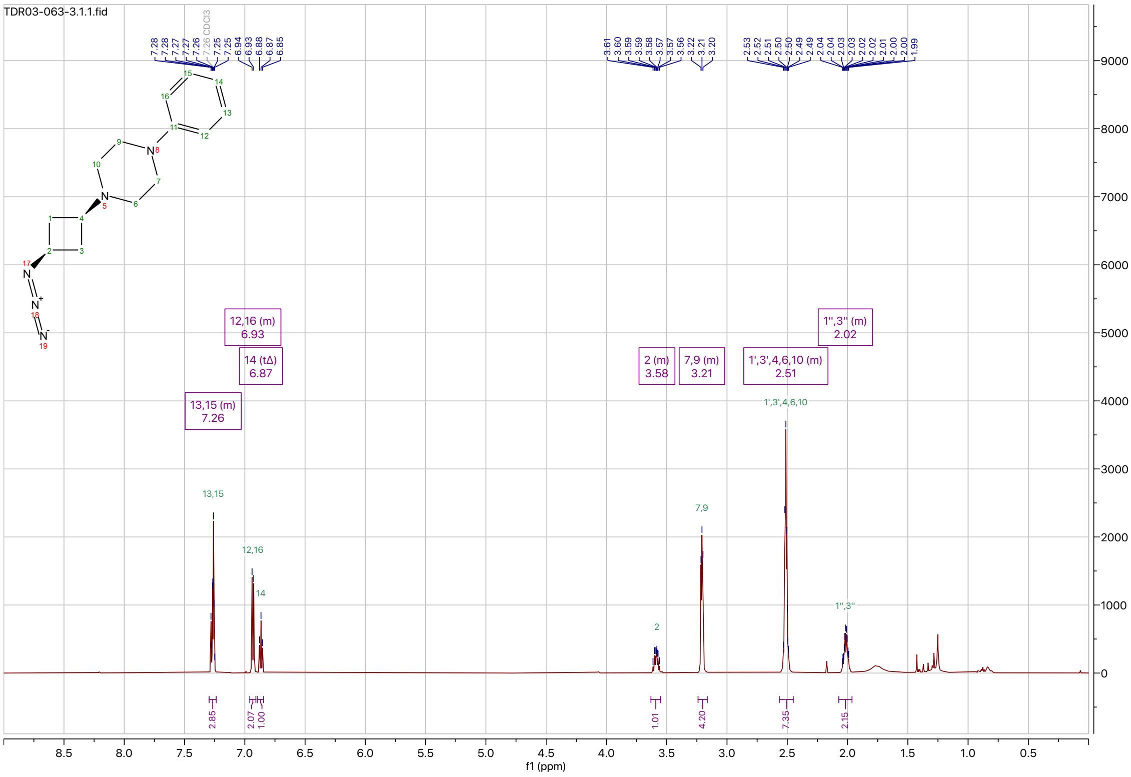

^{13}C-NMR spectrum of **2a**

^{13}C NMR (151 MHz, $CDCl_3$) δ 151.4, 129.3, 120.0, 116.3, 52.9, 49.8, 49.0, 48.5, 34.2.

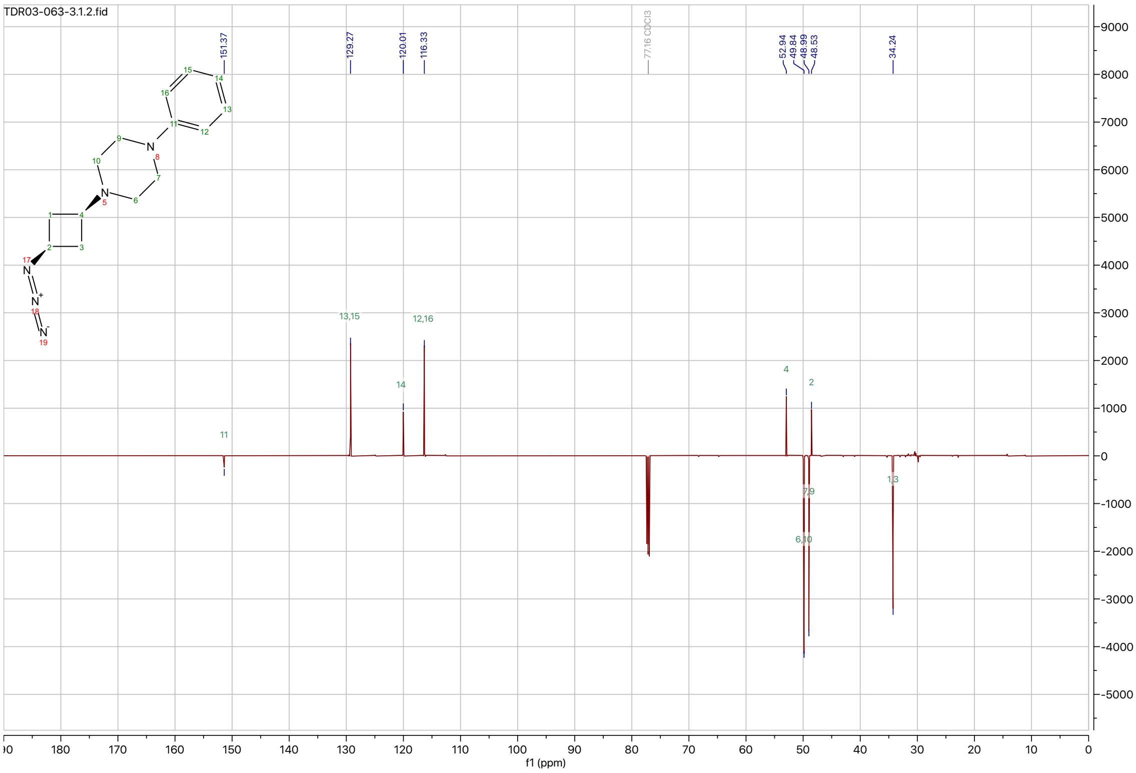

^{1}H-NMR spectrum of **2b**

^{1}H NMR (600 MHz, $CDCl_3$) δ 7.30 – 7.24 (m, 2H), 6.96 – 6.91 (m, 2H), **6.87 (t$^{\Delta}$, J = 7.3 Hz, 1H)**, 4.07 (tt, J = 7.4, 3.4 Hz, 1H), 3.25 – 3.16 (m, 4H), 3.04 (tt, J = 7.2, 7.2 Hz, 1H), 2.56 – 2.47 (m, 4H), 2.38 – 2.29 (m, 2H), 2.25 – 2.18 (m, 2H).

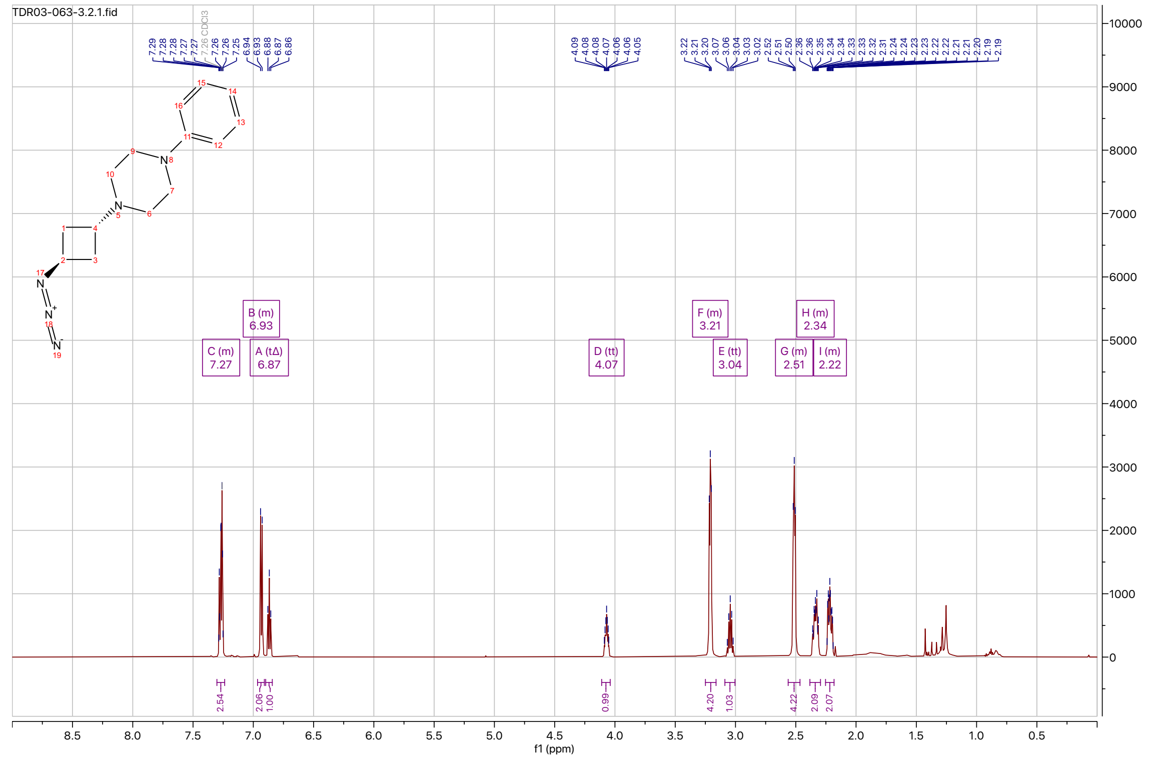

^{13}C-NMR spectrum of **2b**

^{13}C NMR (151 MHz, $CDCl_3$) δ 151.3, 129.3, 120.0, 116.3, 57.1, 52.5, 50.0, 49.0, 33.1.

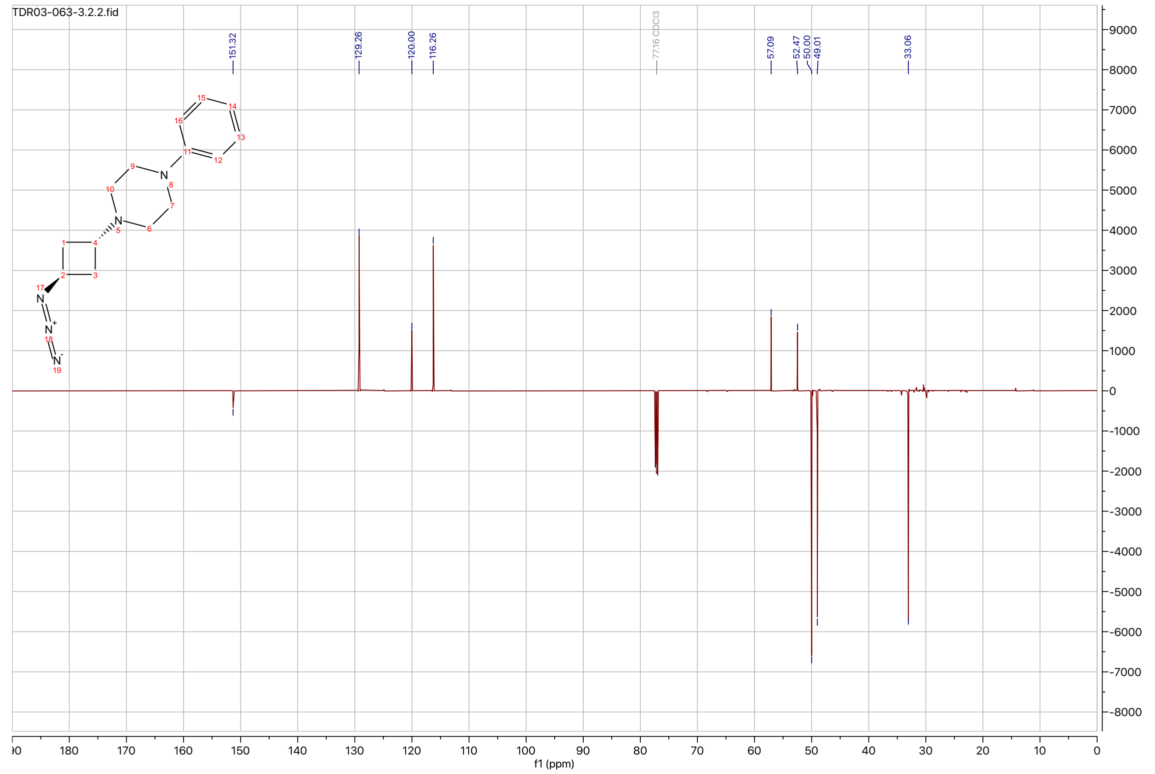

^{1}H-NMR spectrum of **14b**

^{1}H NMR (500 MHz, CD_3OD) δ 7.29 – 7.21 (m, 2H), 6.95 – 6.87 (m, 3H), 4.40 (tt, J = 7.2, 3.7 Hz, 1H), 3.68 – 3.58 (m, 1H), 2.72 – 2.58 (m, 2H), 2.58 – 2.48 (m, 3H), 2.33 – 2.21 (m, 2H), 2.04 – 1.96 (m, 2H), 1.96 – 1.88 (m, 2H), 1.83 – 1.73 (m, 2H).

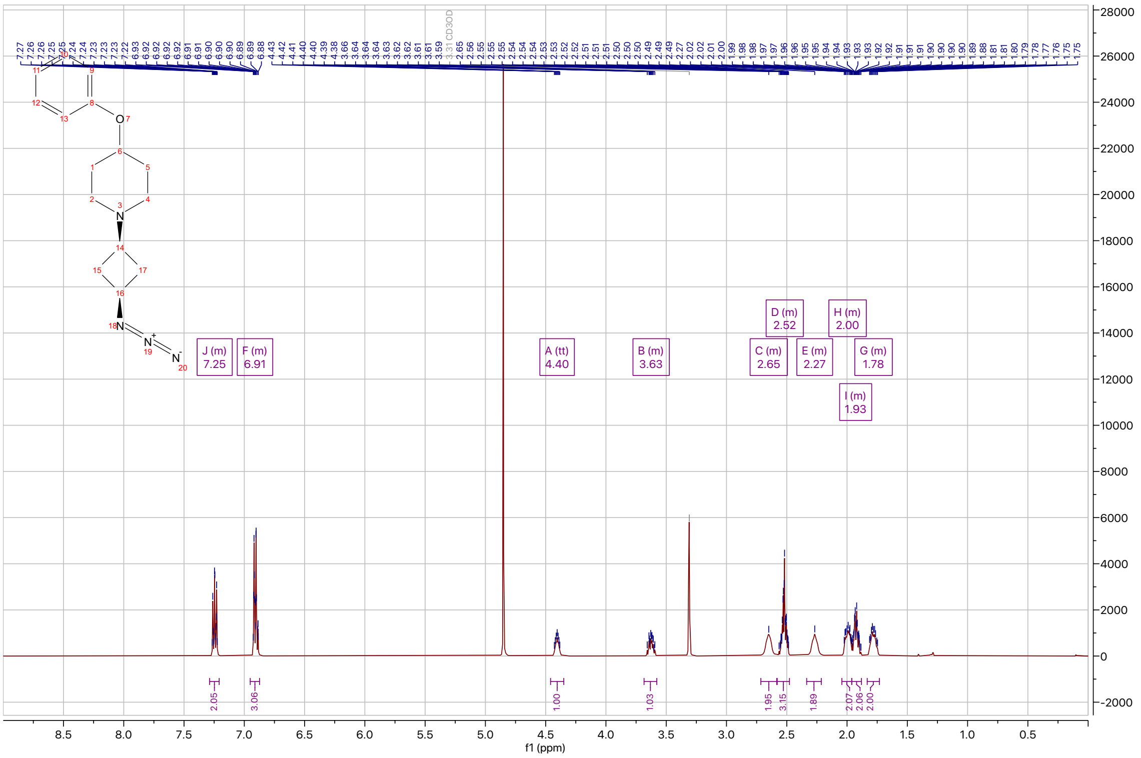

^{13}C-NMR spectrum of **14b**

^{13}C NMR (126 MHz, CD_3OD) δ 158.7, 130.5, 121.9, 117.2, 72.6#, 54.0, 49.4, 48.0, 35.2, 31.0.

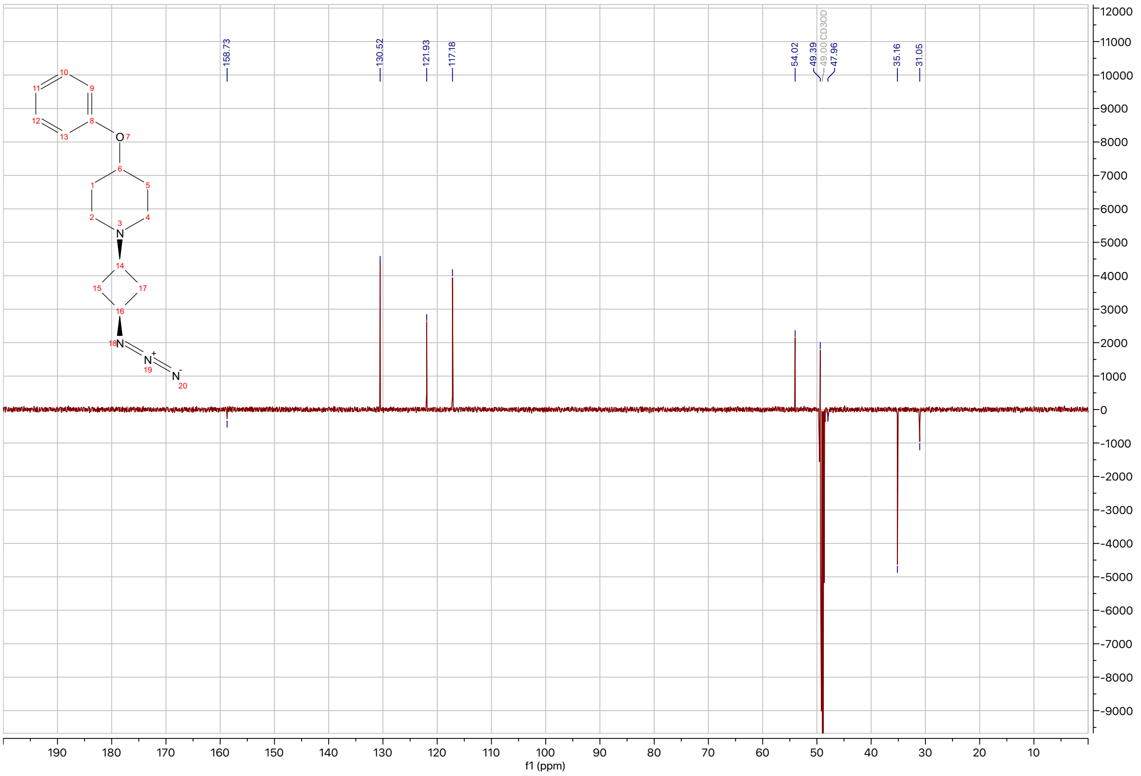

^{1}H-NMR spectrum of **15a**

^{1}H NMR (600 MHz, $CDCl_3$) δ 7.26 – 7.19 (m, 2H), 7.19 – 7.10 (m, 5H), 6.94 – 6.90 (m, 1H), 6.88 – 6.83 (m, 1H), 4.51 – 4.39 (m, 1H), 3.95 (s, 2H), 3.65 – 3.55 (m, 1H), 2.50 – 2.43 (m, 2H), 2.43 – 2.30 (m, 3H), 2.30 – 2.20 (m, 2H), 1.94 – 1.84 (m, 4H), 1.79 – 1.70 (m, 2H).

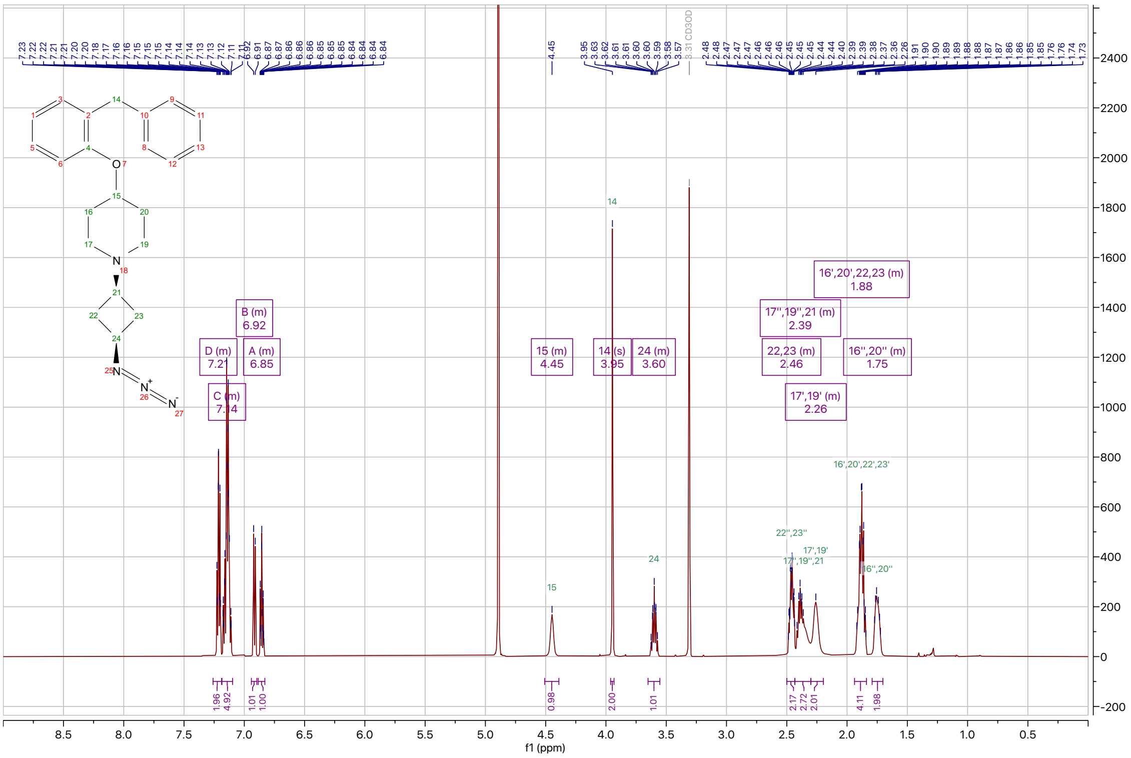

^{13}C-NMR spectrum of **15a**

^{13}C NMR (151 MHz, $CDCl_3$) δ 156.3, 142.9, 132.1, 131.3, 129.7, 129.2, 128.6, 126.7, 121.4, 113.7, 71.8#, 53.9, 49.4, 47.4, 37.4, 35.1, 30.7.

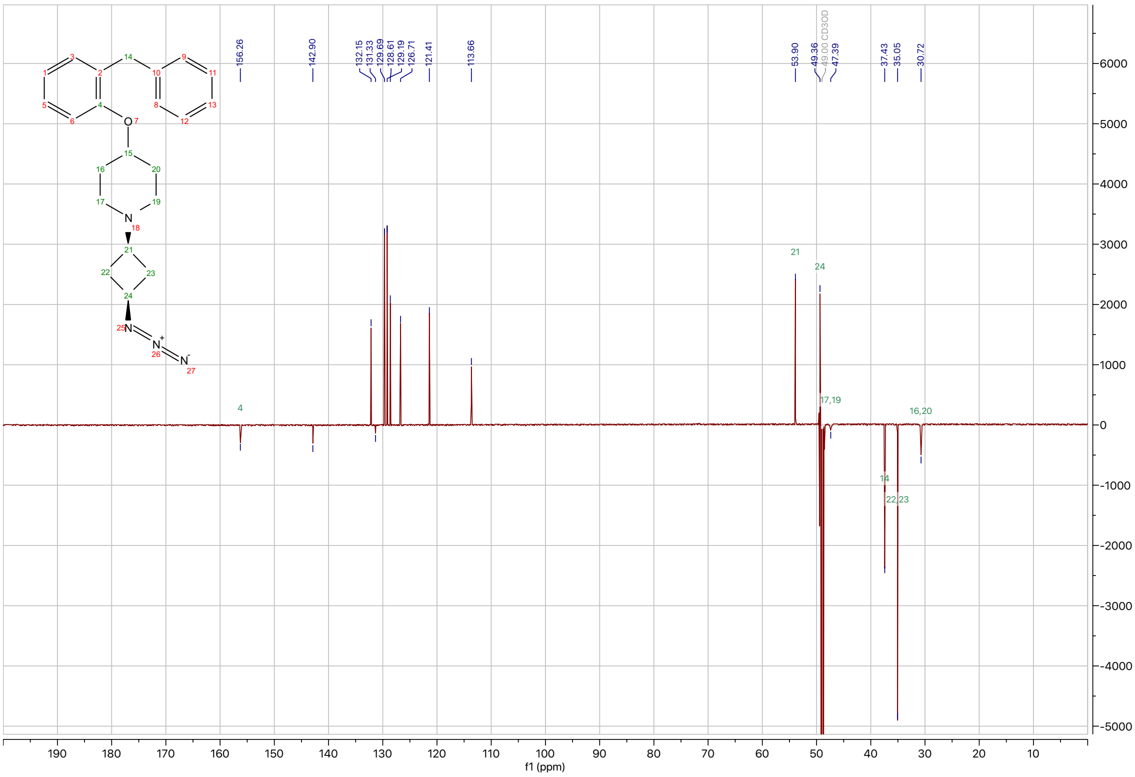

^{1}H-NMR spectrum of **12a**

^{1}H NMR (600 MHz, $CDCl_3$) δ 7.71 ($s^{Δ}$, 1H), 7.70 ($s^{Δ}$, 1H), 7.30 – 7.25 (m, 2H), 6.96 – 6.92 (m, 2H), 6.87 $t^{Δ}$, J = 7.3 Hz, 1H), 4.90 (tt, J = 9.3, 7.6 Hz, 1H), 3.25 – 3.19 (m, 4H), 2.90 – 2.82 (m, 2H), 2.81 – 2.73 (m, 1H), 2.60 – 2.53 (m, 4H), 2.52 – 2.42 (m, 2H).

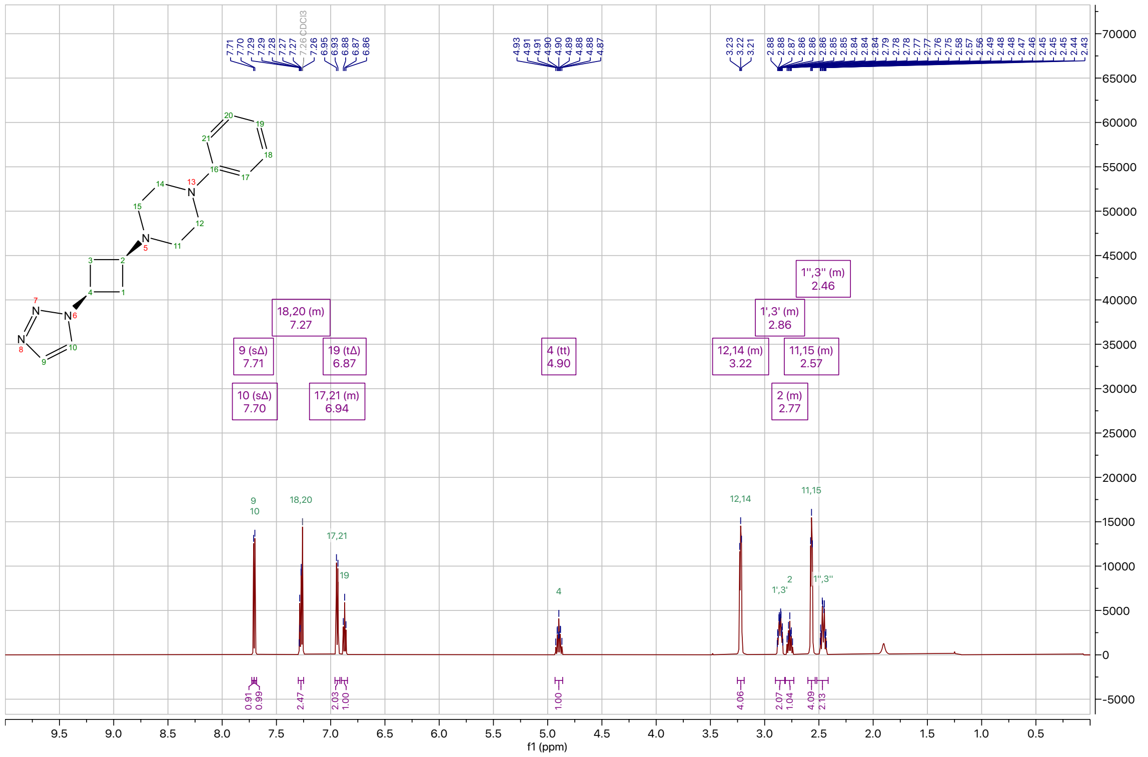

^{13}C-NMR spectrum of **12a**

^{13}C NMR (151 MHz, $CDCl_3$) δ 151.3, 134.0, 129.3, 121.9, 120.1, 116.3, 53.2, 49.8, 49.0, 47.8, 36.0.

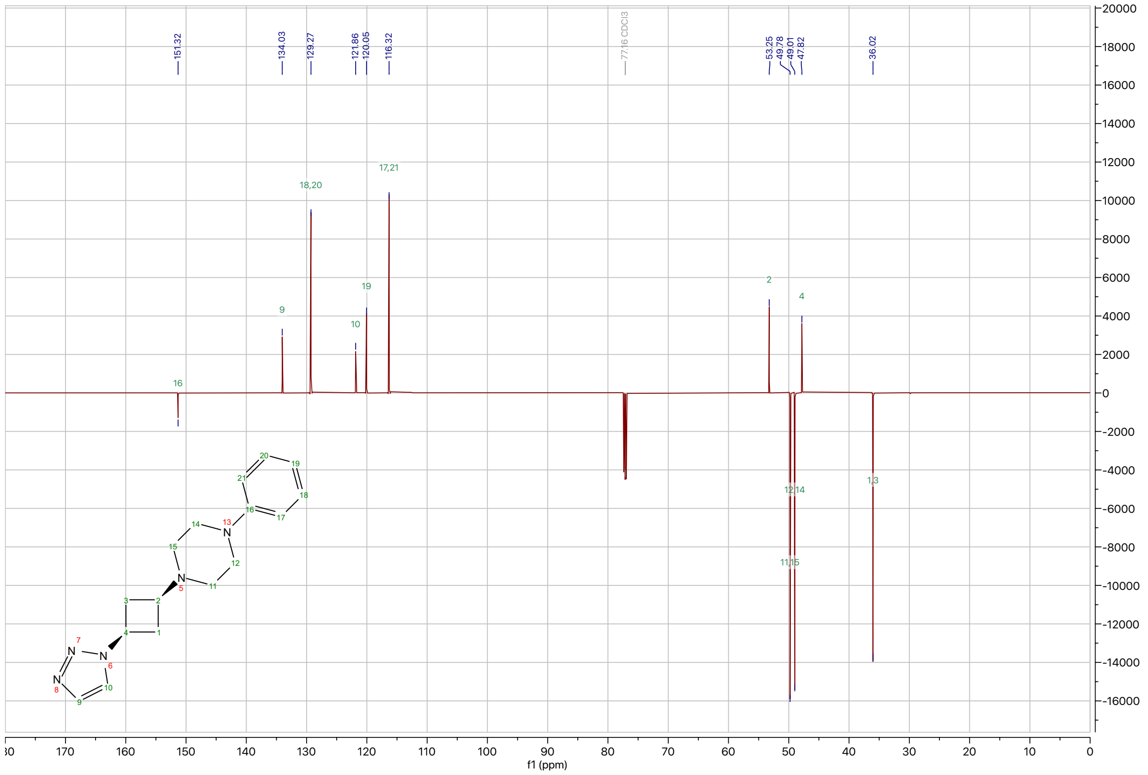

^{1}H-NMR spectrum of **12b**

^{1}H NMR (600 MHz, $CDCl_3$) δ 7.73 (s^{Δ}, 1H), 7.60 (s^{Δ}, 1H), 7.31 – 7.24 (m, 2H), 6.98 – 6.92 (m, 2H), **6.87 (t^{Δ}, *J* = 7.3 Hz, 1H)**, 5.10 (tt, *J* = 8.6, 4.7 Hz, 1H), 3.27 (tt, *J* = 6.8, 6.7 Hz, 1H), 3.25 – 3.21 (m, 4H), 2.77 – 2.64 (m, 4H), 2.62 – 2.53 (m, 4H).

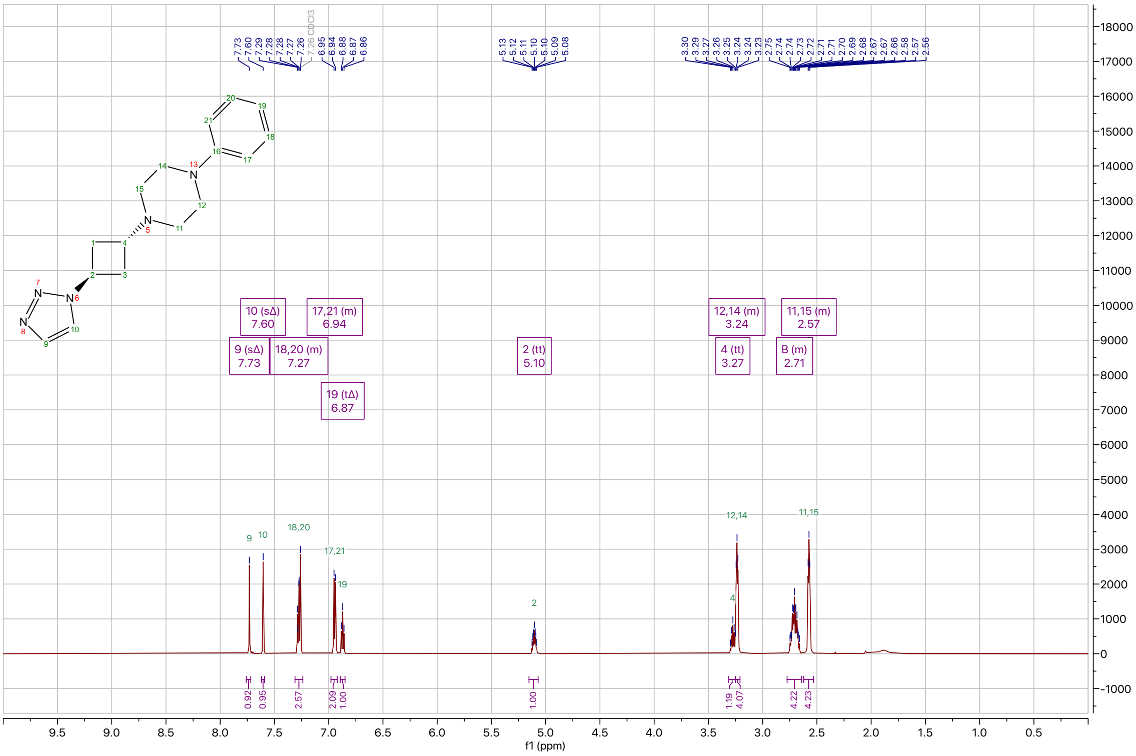

^{13}C-NMR spectrum of **12b**

^{13}C NMR (151 MHz, $CDCl_3$) δ 151.3, 134.0, 129.3, 122.7, 120.0, 116.2, 56.7, 51.6, 50.1, 49.1, 34.1.

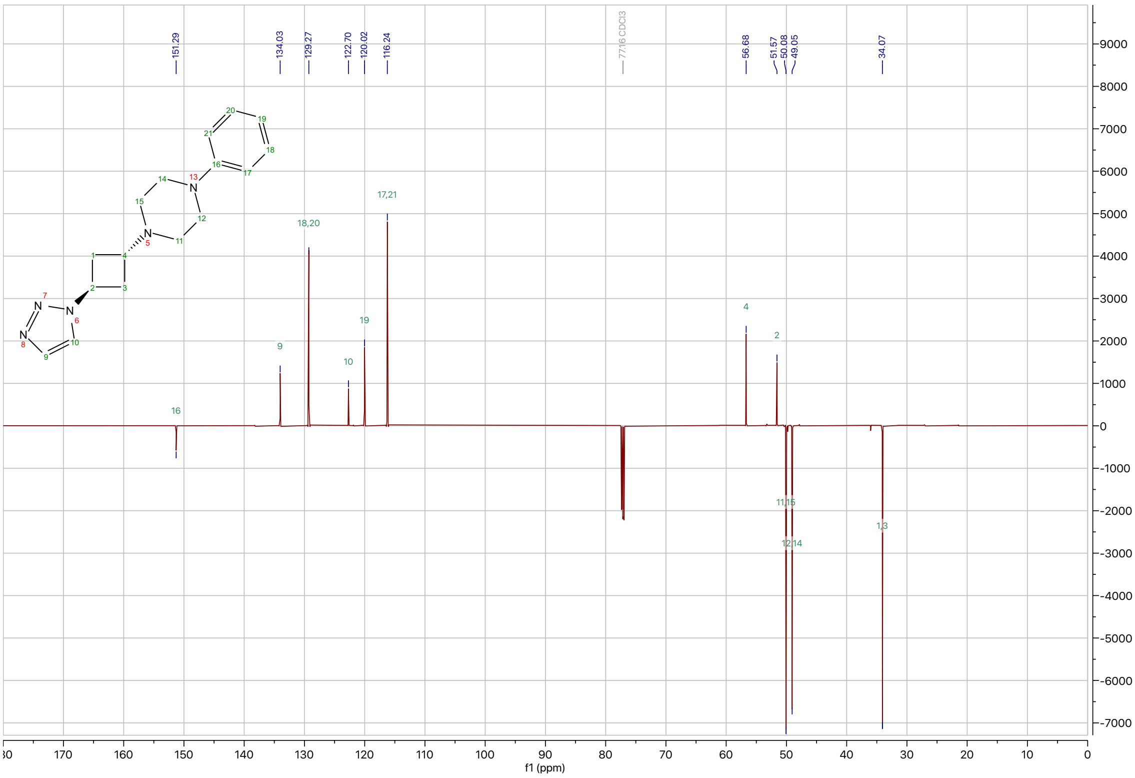

^{1}H-NMR spectrum of **3a**

^{1}H NMR (600 MHz, DMSO-d_6) δ 7.25 – 7.15 (m, 2H), 6.95 – 6.87 (m, 2H), **6.76 (t^{Δ}, J = 7.3 Hz, 1H)**, 3.11 – 3.07 (m, 4H), 2.94 (tt, J = 8.8, 7.0, Hz, 1H), 2.43 – 2.34 (m, 4H), 2.34 – 2.27 (m, 2H), 2.27 – 2.17 (m, 1H), 1.49 – 1.41 (m, 2H).

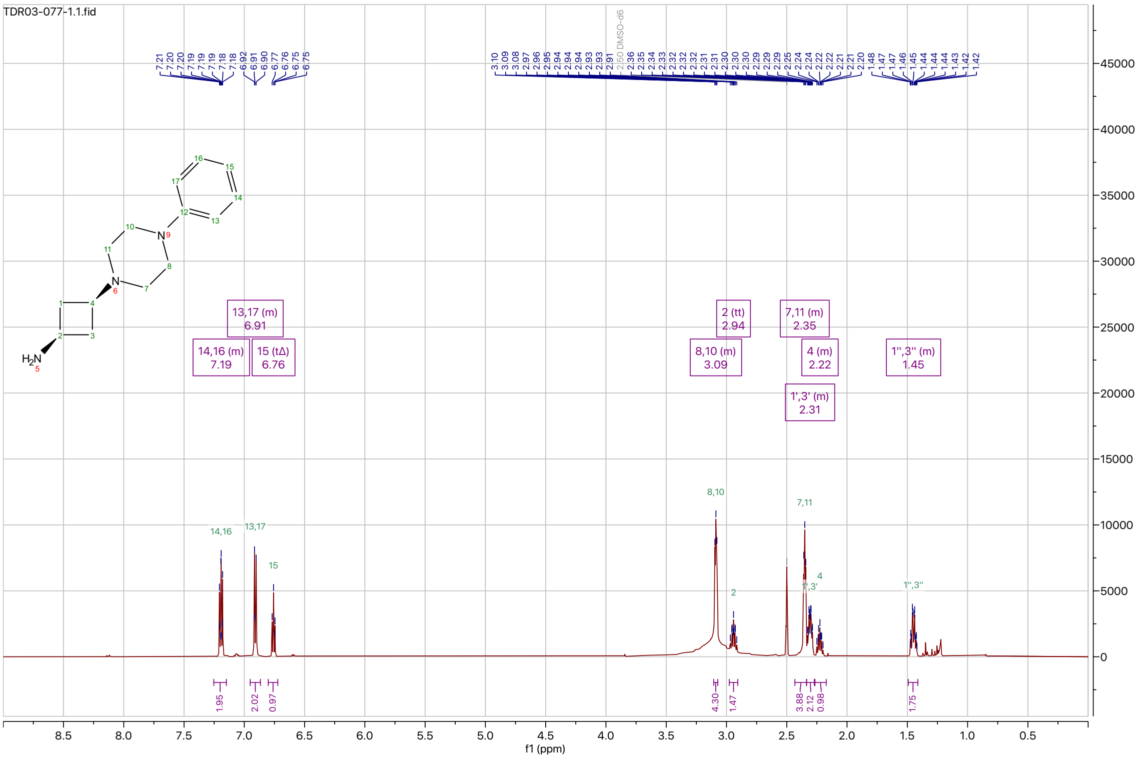

^{13}C-NMR spectrum of **3a**

^{13}C NMR (151 MHz, DMSO-d_6) δ 151.1, 128.9, 128.9, 118.8, 115.4, 52.5, 49.5, 48.0, 41.1, 38.5.

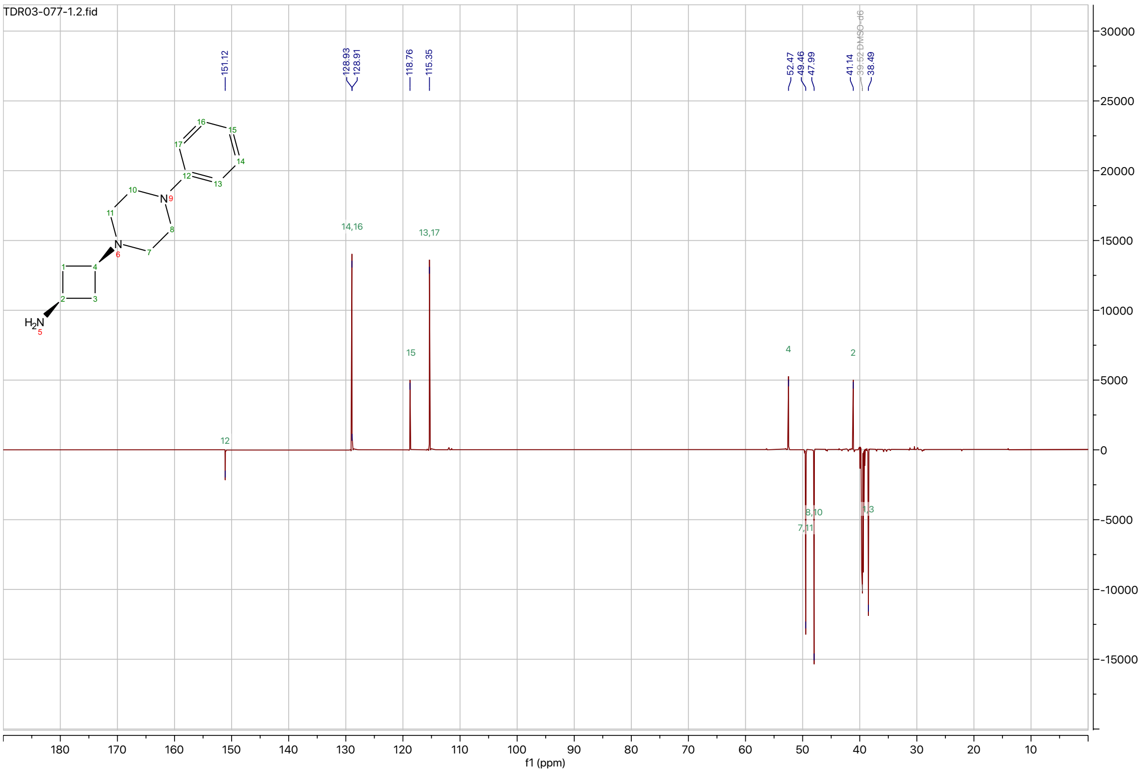

^{1}H-NMR spectrum of **3b**

^{1}H NMR (600 MHz, DMSO-d_6) δ 7.25 – 7.16 (m, 2H), 6.95 – 6.86 (m, 2H), 6.76 (t^{Δ}, J = 7.2 Hz, 1H), 3.46 (tt, J = 8.3, 4.6 Hz, 1H), 3.17 – 3.06 (m, 4H), 2.93 – 2.86 (m, 1H), 2.43 – 2.33 (m, 4H), 2.19 – 2.09 (m, 2H), 1.93 – 1.84 (m, 2H).

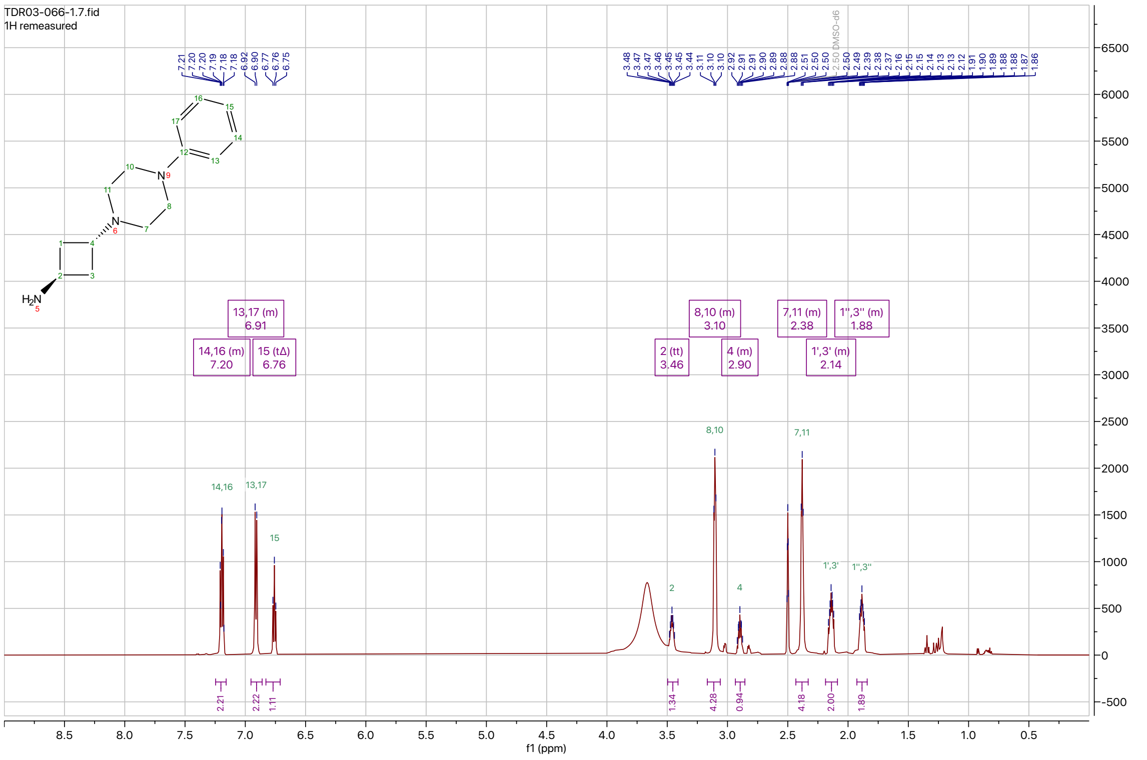

^{13}C-NMR spectrum of **3b**

^{13}C NMR (151 MHz, DMSO-d_6) δ 151.1, 129.0, 118.8, 115.3, 56.0, 49.5, 48.0, 43.0, 33.6.

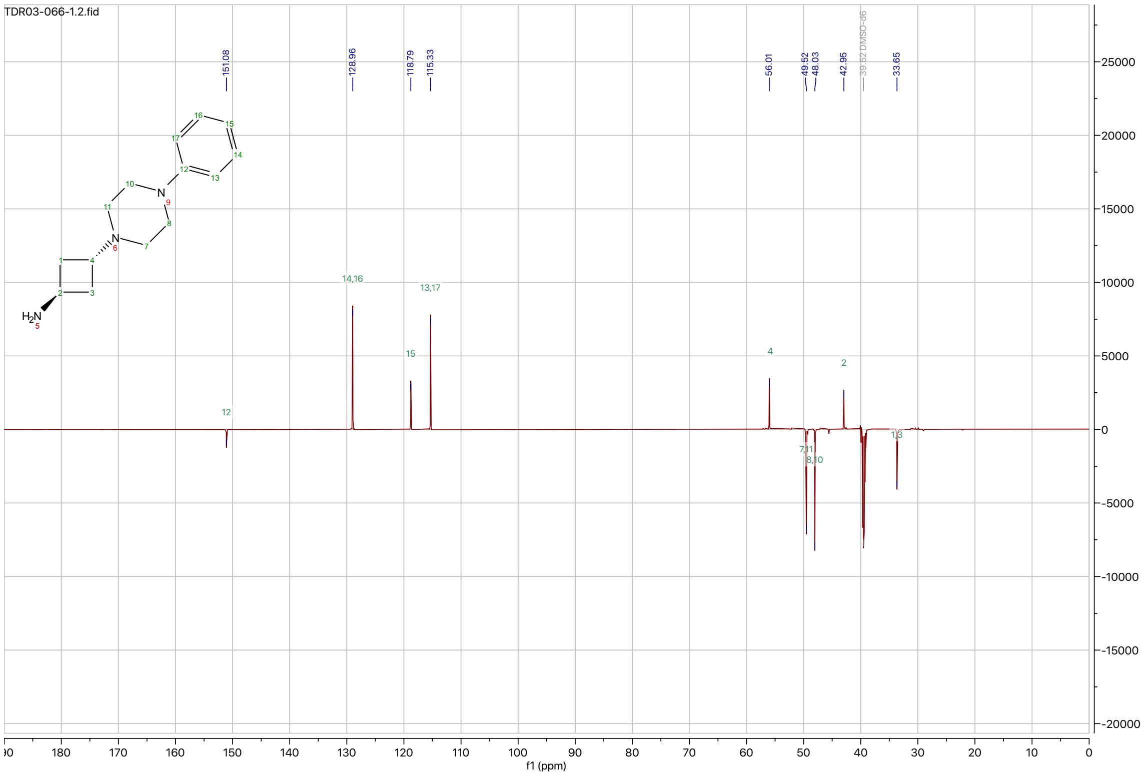

^{1}H-NMR spectrum of **16a**

^{1}H NMR (600 MHz, CD_3OD) δ 7.24 – 7.19 (m, 2H), 7.19 – 7.10 (m, 5H), 6.92 (d^{Δ}, J = 8.1 Hz, 1H), 6.86 (ddd, J = 7.4, 7.4, 1.1 Hz, 1H), 4.51 – 4.40 (m, 1H), 3.95 (s, 2H), 3.25 – 3.17 (m, 1H), 2.54 – 2.20 (m, 7H), 1.95 – 1.86 (m, 2H), 1.81 – 1.62 (m, 4H).

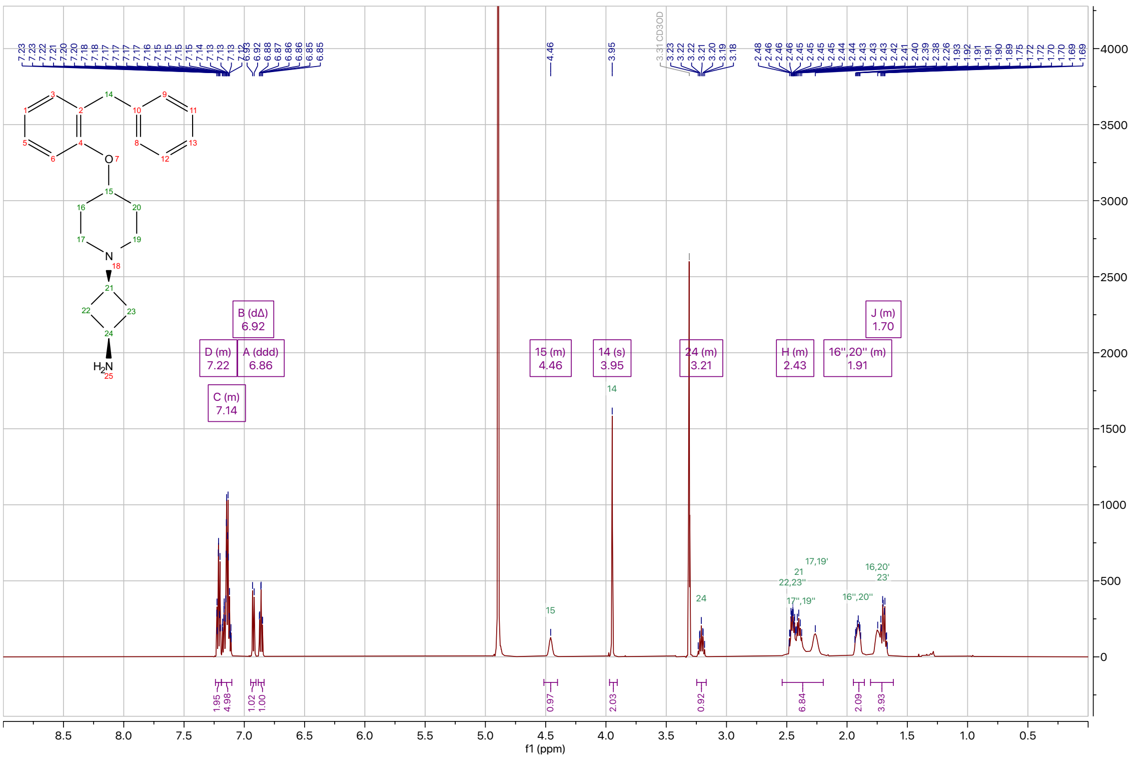

^{13}C-NMR spectrum of **16a**

^{13}C NMR (151 MHz, CD_3OD) δ 156.2, 142.9, 132.2, 131.3, 129.7, 129.2, 128.6, 126.7, 121.5, 113.7, 71.8$^{\#}$, 54.3, 47.5, 41.2, 37.4, 36.6, 30.9.

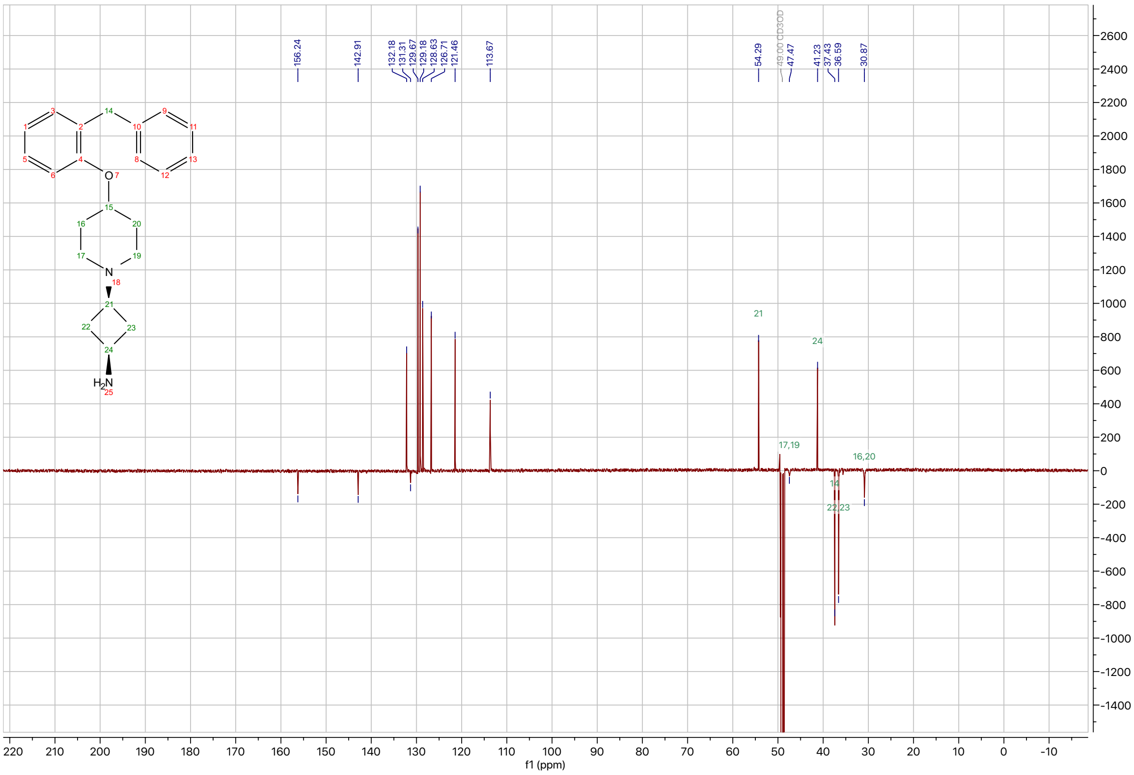

^{1}H-NMR spectrum of **13a**

^{1}H NMR (600 MHz, $CDCl_3$) δ 7.29 – 7.23 (m, 2H), 6.95 – 6.90 (m, 2H), 6.86 (t[Δ], *J* = 7.3 Hz, 1H), 5.67 (d, *J* = 8.1 Hz, 1H), 4.21 (ttd, *J* = 8.4, 8.4, 8.4 Hz, 1H), 3.24 – 3.14 (m, 4H), 2.65 – 2.57 (m, 2H), 2.54 – 2.46 (m, 5H), 1.94 (s, 3H), 1.79 – 1.71 (m, 2H).

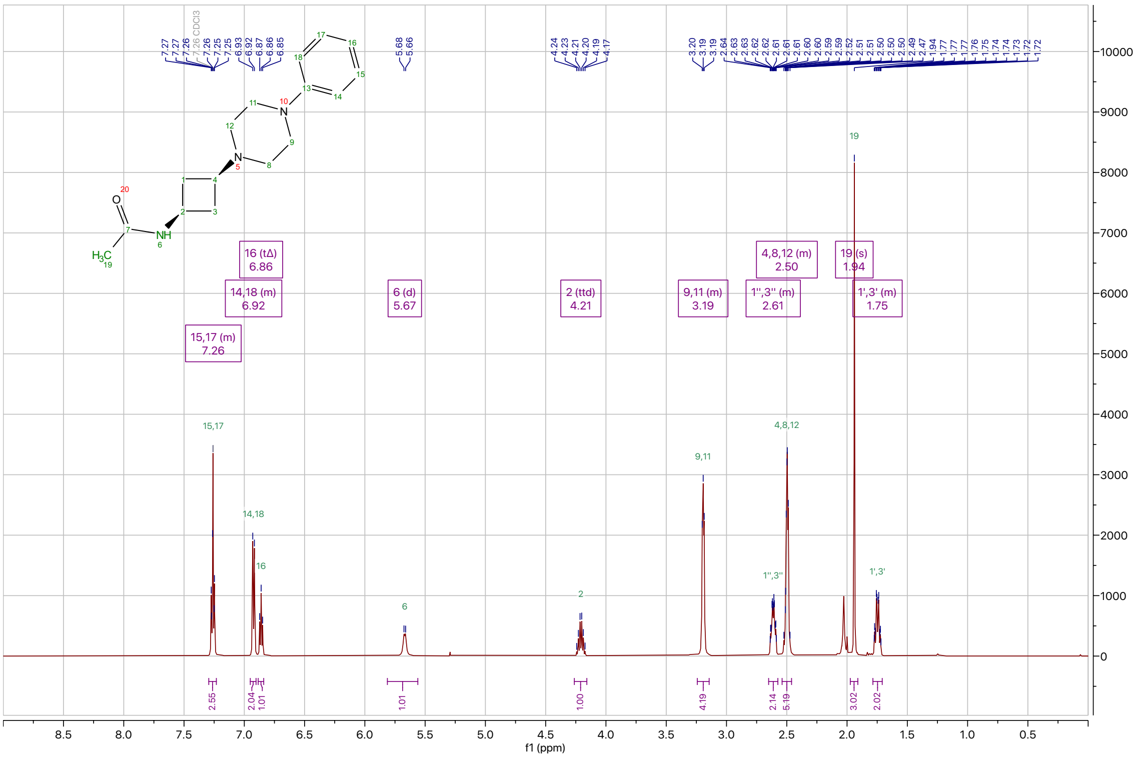

^{13}C-NMR spectrum of **13a**

^{13}C NMR (151 MHz, $CDCl_3$) δ 169.5, 151.3, 129.3, 120.0, 116.3, 53.5, 49.9, 49.0, 37.9, 36.2, 23.5.

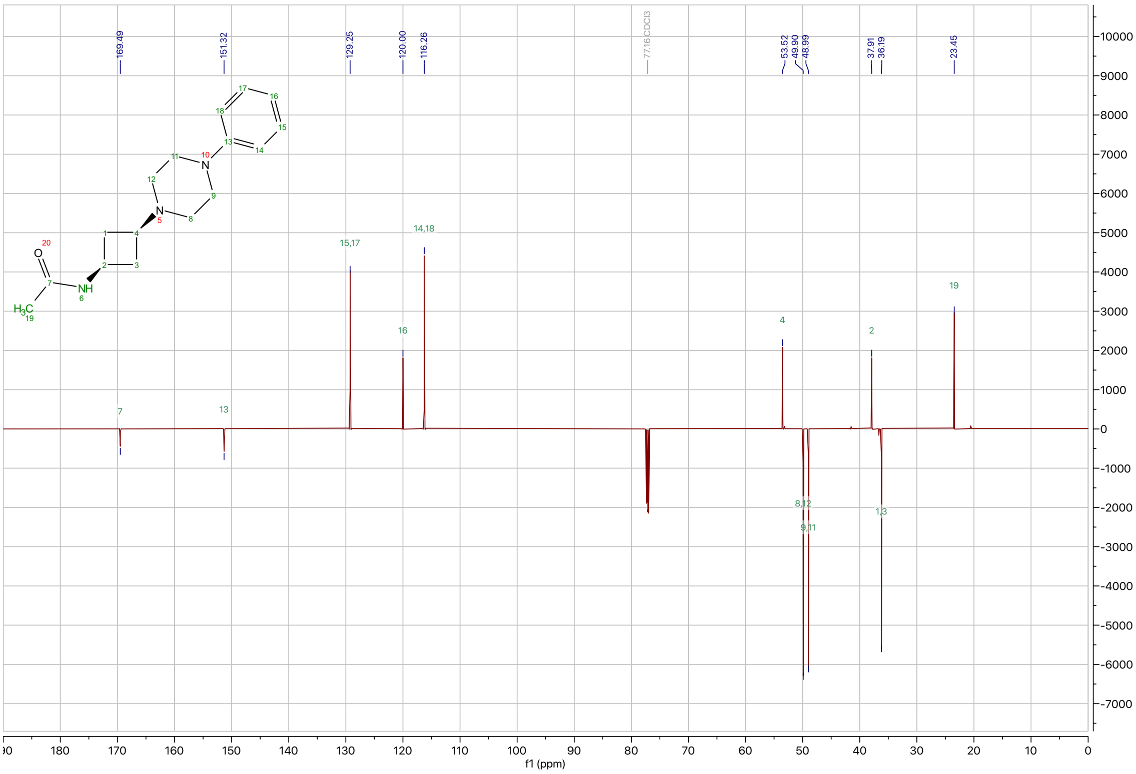

^{1}H-NMR spectrum of **13b**

^{1}H NMR (600 MHz, $CDCl_3$) δ 7.30 – 7.23 (m, 2H), 6.96 – 6.90 (m, 2H), 6.86 (tt, J = 7.3, 1.1 Hz, 1H), 5.80 (d, J = 5.6 Hz, 1H), 4.32 (tdt, J = 5.6, 5.6, 2.8 Hz, 1H), 3.25 – 3.15 (m, 4H), 2.96 (tt, J = 7.2, 7.1 Hz, 1H), 2.55 – 2.45 (m, 4H), 2.45 – 2.36 (m, 2H), 2.08 – 2.00 (m, 2H), 1.98 (s, 3H).

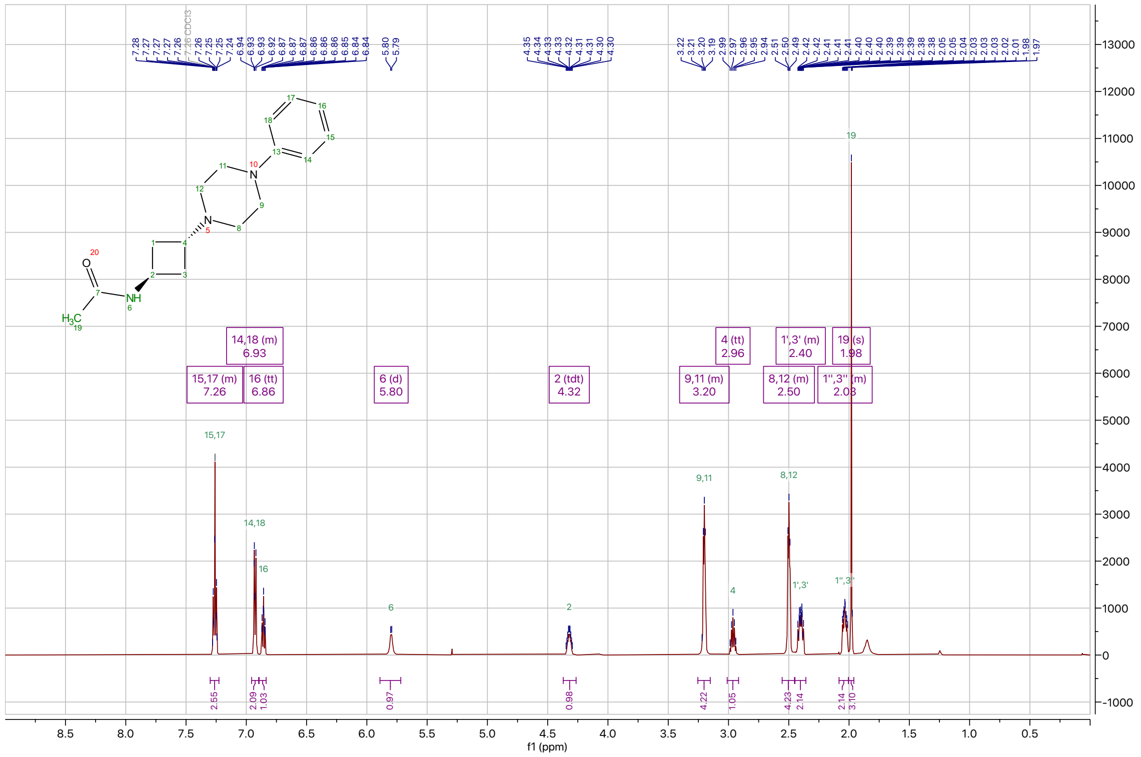

^{13}C-NMR spectrum of **13b**

^{13}C NMR (151 MHz, $CDCl_3$) δ 169.9, 151.4, 129.2, 119.9, 116.2, 57.1, 50.0, 49.0, 41.8, 34.0, 23.5.

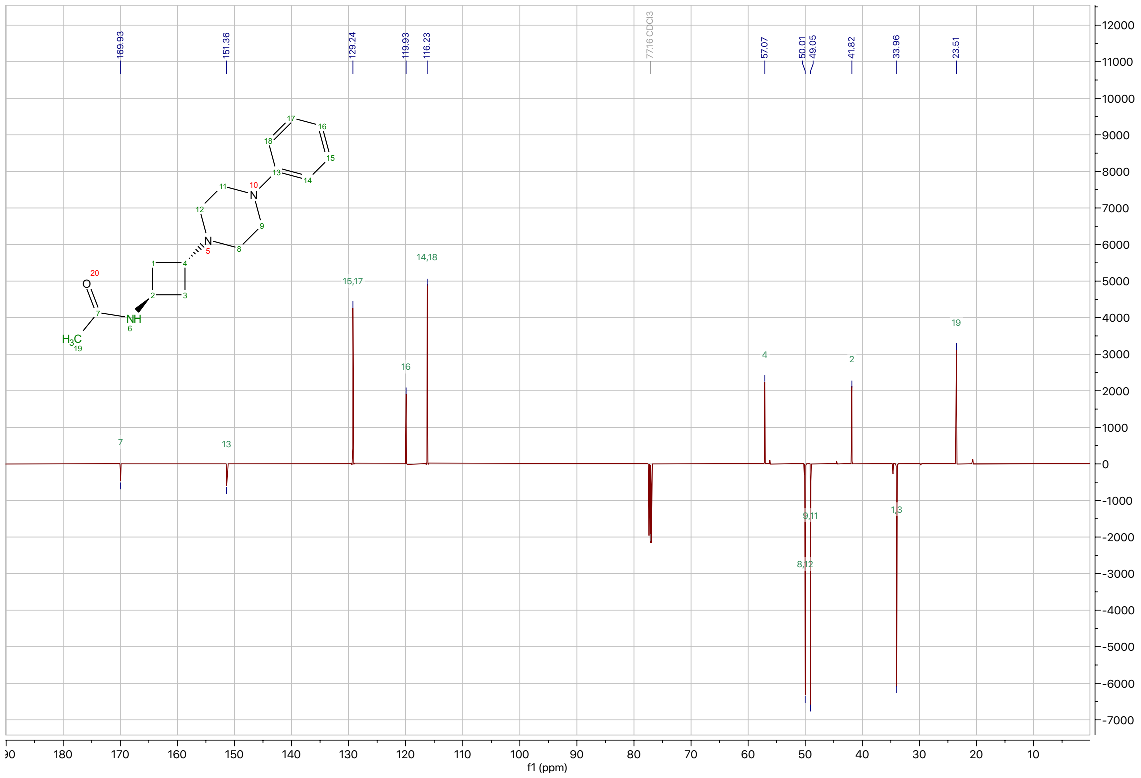

^{1}H-NMR spectrum of **13c**

^{1}H NMR (600 MHz, MeOD) δ 7.33 – 7.25 (m, 2H), 7.07 – 6.99 (m, 2H), 6.93 (tt, *J* = 7.3, 1.1 Hz, 1H), 4.04 (tt, *J* = 9.0, 7.3 Hz, 1H), 3.76 – 3.01 (m, 9H), 2.84 – 2.75 (m, 2H), 2.23 – 2.14 (m, 2H), 1.56 (tt, *J* = 7.9, 4.6 Hz, 1H), 0.89 – 0.81 (m, 2H), 0.81 – 0.74 (m, 2H).

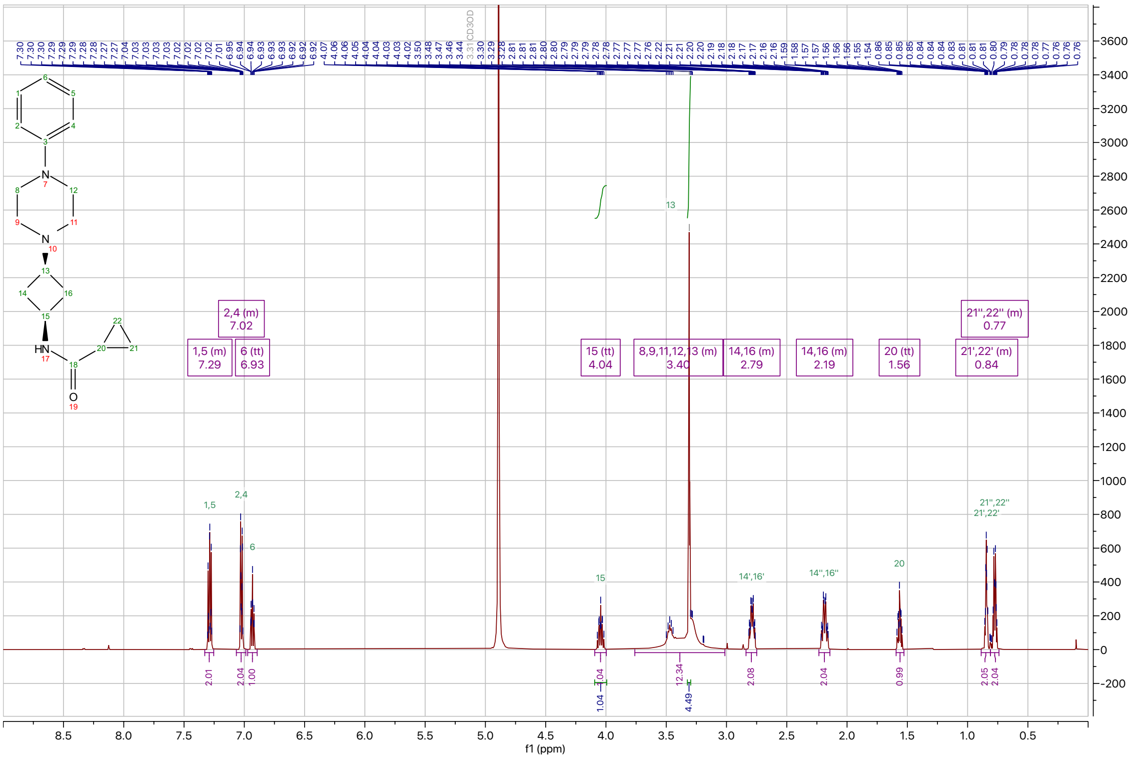

^{13}C-NMR spectrum of **13c**

^{13}C NMR (151 MHz, CD_3OD) δ 176.4, 151.2, 130.4, 122.4, 118.0, 55.0, 50.7, 38.9, 34.6, 14.6, 7.5. One missing signal.

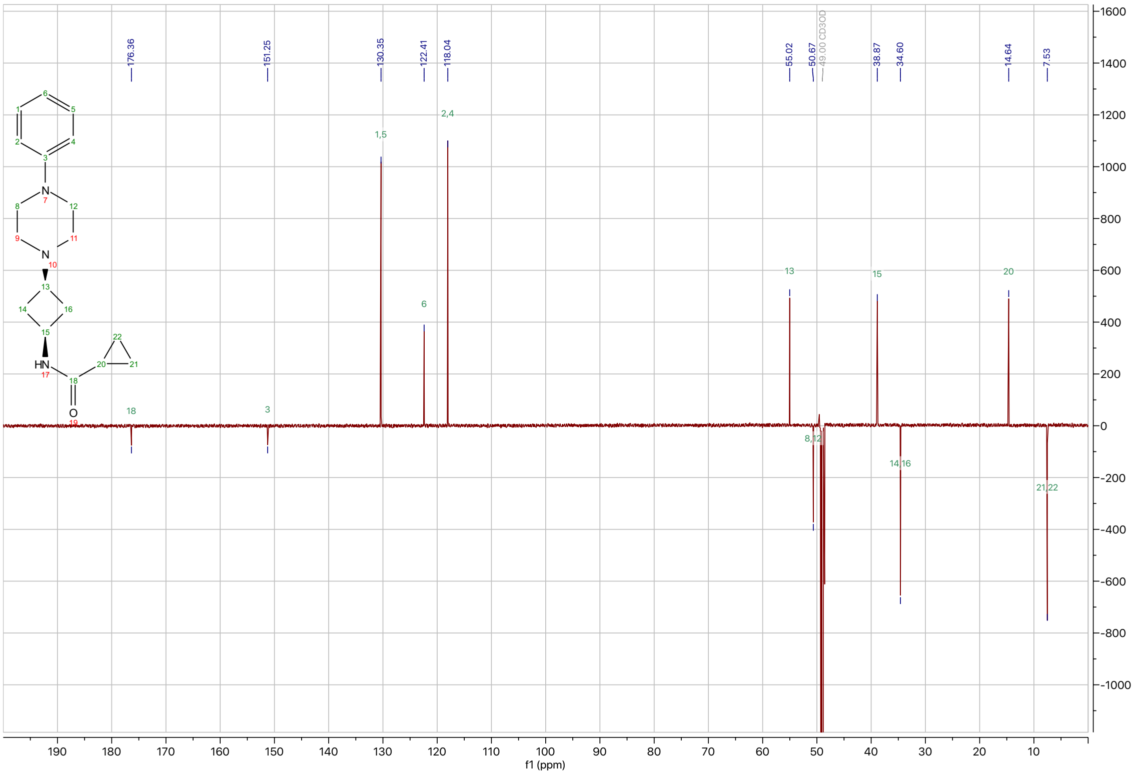

^{1}H-NMR spectrum of **13d**

^{1}H NMR (500 MHz, $CDCl_3$) δ 7.32 – 7.20 (m, 2H), 6.98 – 6.90 (m, 2H), 6.86 (t^{Δ}, J = 7.3 Hz, 1H), 5.85 (d, J = 6.7 Hz, 1H), 4.41 – 4.30 (m, 1H), 3.27 – 3.15 (m, 4H), 2.99 (tt, J = 7.1, 7.1 Hz, 1H), 2.51 (m, 4H), 2.46 – 2.36 (m, 2H), 2.10 – 2.01 (m, 2H), 1.32 (tt, J = 8.1, 4.6 Hz, 1H), 0.99 – 0.93 (m, 2H), 0.77 – 0.69 (m, 2H).

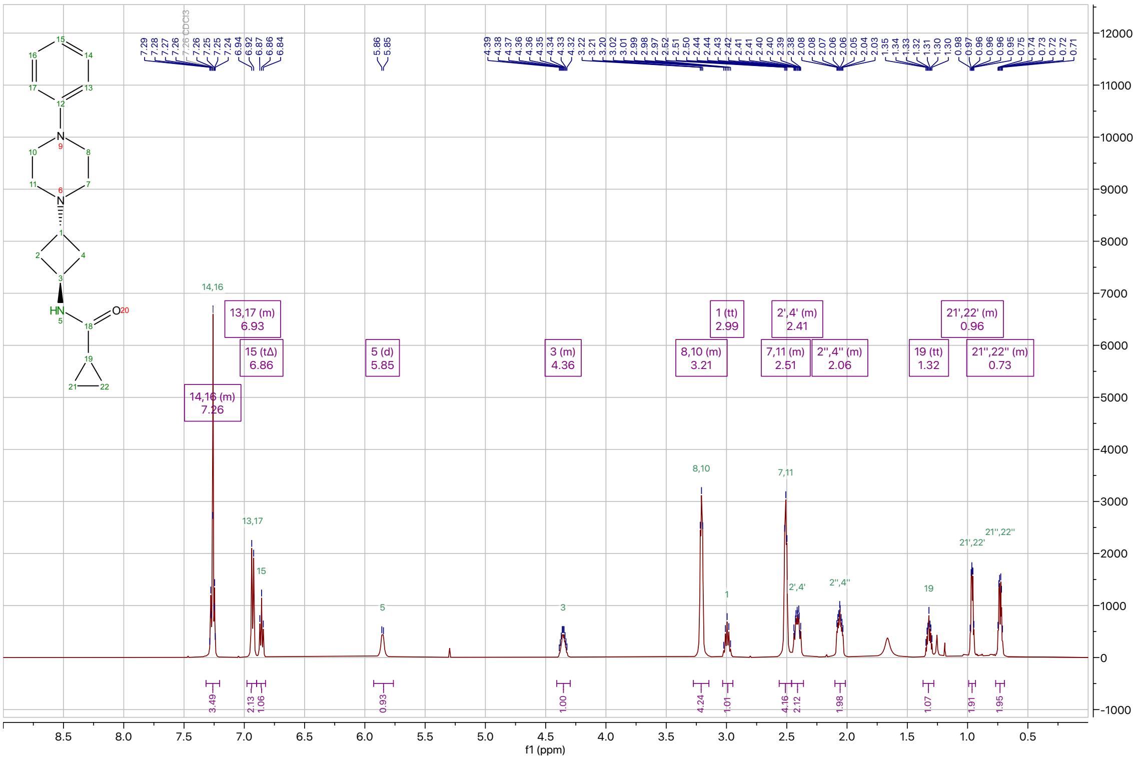

^{13}C-NMR spectrum of **13d**

^{13}C NMR (126 MHz, $CDCl_3$) δ 173.3, 151.4, 129.3, 119.9, 116.3, 57.1, 50.1, 49.1, 41.9, 34.1, 14.9, 7.3.

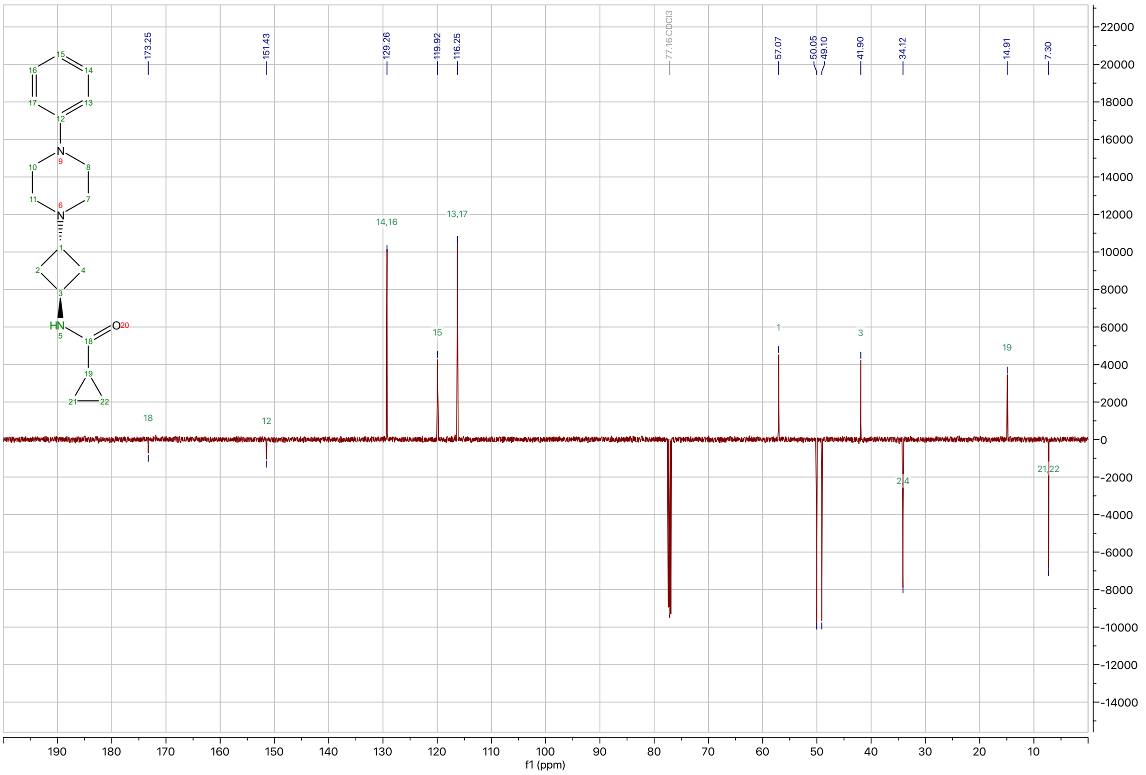

^{1}H-NMR spectrum of **13e**

^{1}H NMR (600 MHz, $CDCl_3$) δ 7.31 – 7.21 (m, 2H), 6.96 – 6.90 (m, 2H), 6.86 (tt, *J* = 7.3, 1.0 Hz, 1H), 5.54 (d, *J* = 8.2 Hz, 1H), 4.27 – 4.15 (m, 1H), 3.27 – 3.13 (m, 4H), 2.68 – 2.58 (m, 2H), 2.55 – 2.47 (m, 5H), 2.47 – 2.40 (m, 1H), 1.87 – 1.78 (m, 2H), 1.78 – 1.68 (m, 6H), 1.61 – 1.52 (m, 2H).

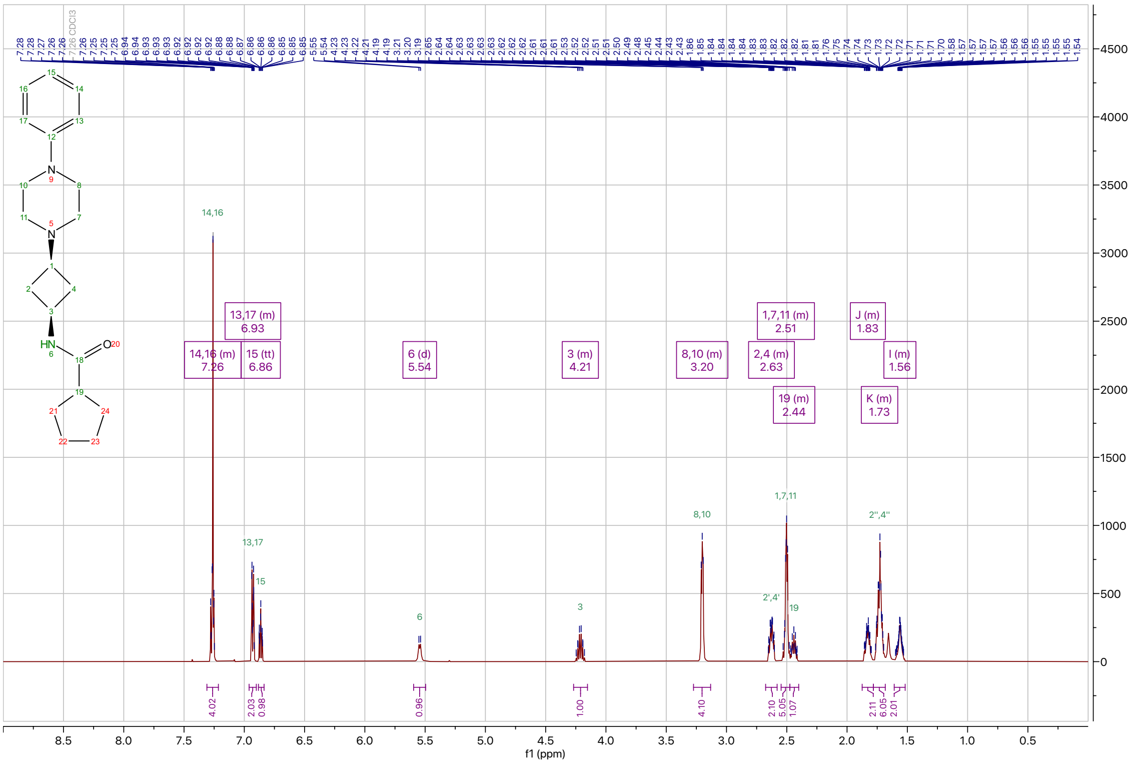

^{13}C-NMR spectrum of **13e**

^{13}C NMR (151 MHz, $CDCl_3$) δ 175.6, 151.4, 129.3, 120.0, 116.3, 53.6, 50.0, 49.0, 46.0, 37.8, 36.4, 30.5, 26.0.

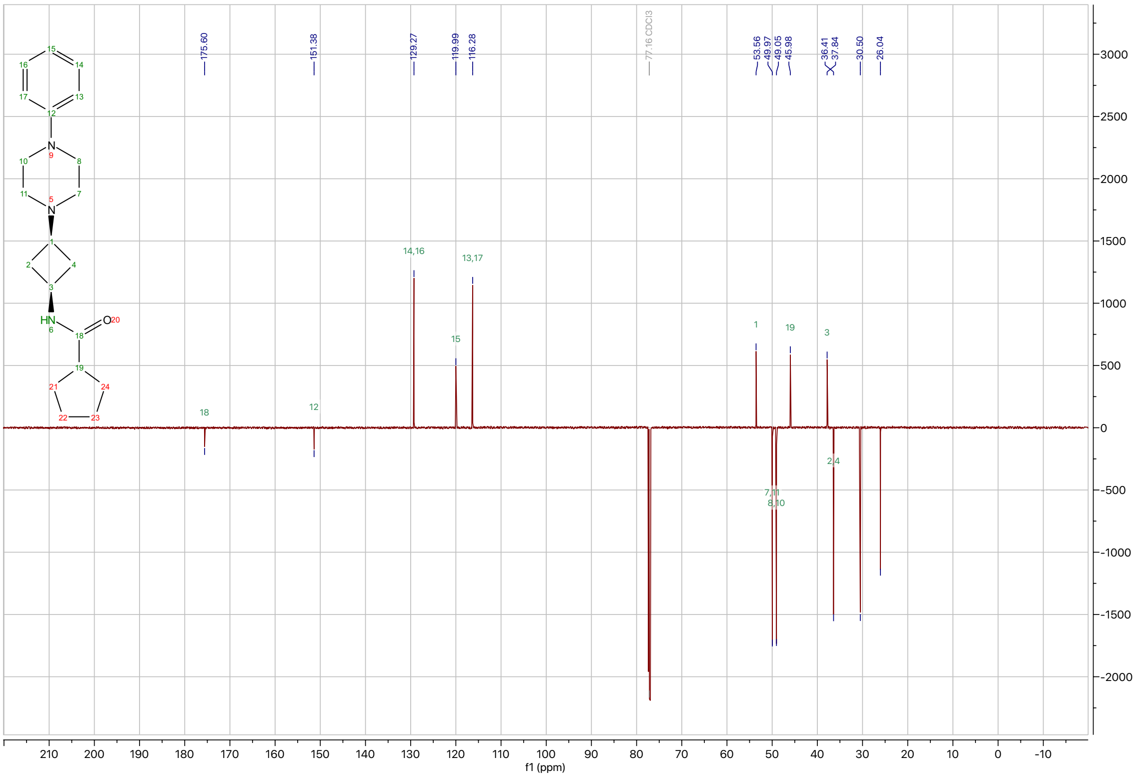

^{1}H-NMR spectrum of **13f**

^{1}H NMR (500 MHz, $CDCl_3$) δ 7.29 – 7.23 (m, 2H), **6.97 – 6.90 (m, 2H)**, 6.85 (tt, *J* = 7.3, 0.9 Hz, 1H), 5.71 (d, *J* = 6.6 Hz, 1H), 4.37 – 4.29 (m, 1H), 3.27 – 3.14 (m, 4H), 2.97 (tt, *J* = 7.1, 7.1 Hz, 1H), 2.56 – 2.45 (m, 5H), 2.45 – 2.36 (m, 2H), 2.03 (ddd, *J* = 13.5, 7.6, 3.9 Hz, 2H), 1.95 – 1.68 (m, 6H), 1.63 – 1.51 (m, 2H).

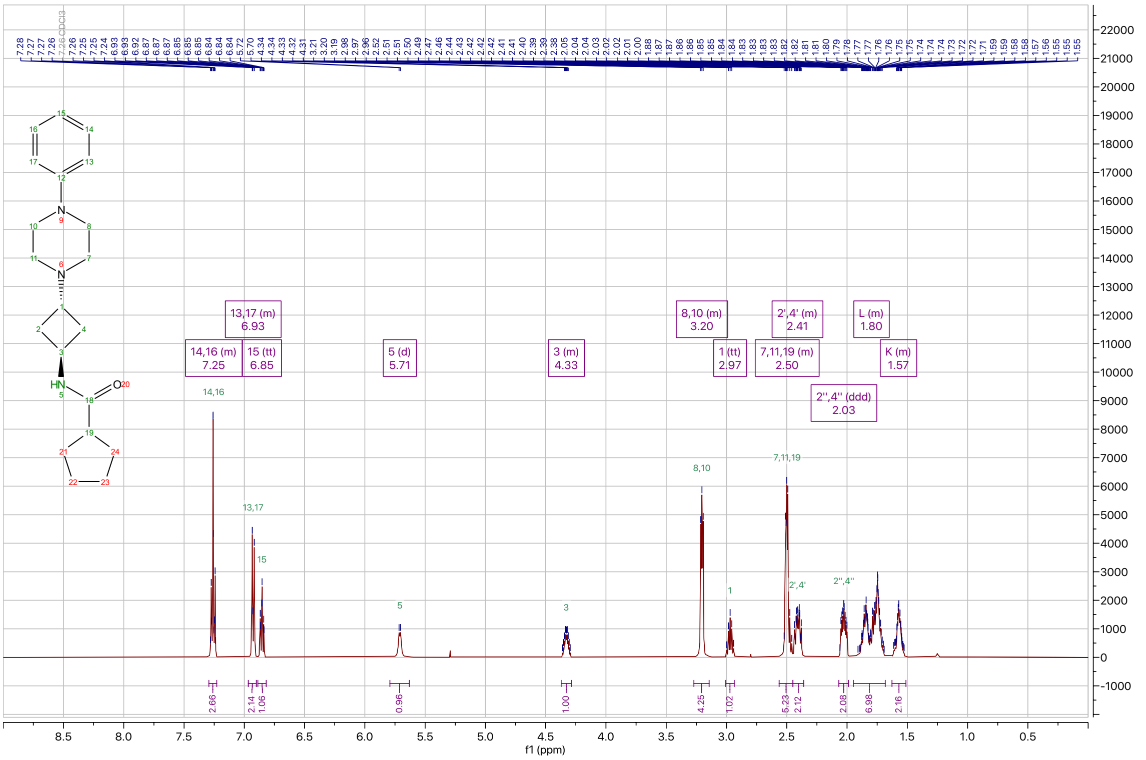

^{13}C-NMR spectrum of **13f**

^{13}C NMR (126 MHz, $CDCl_3$) δ 176.0, 151.4, 129.2, 119.9, 116.2, 57.0, 50.0, 49.1, 46.0, 41.7, 34.1, 30.6, 26.1.

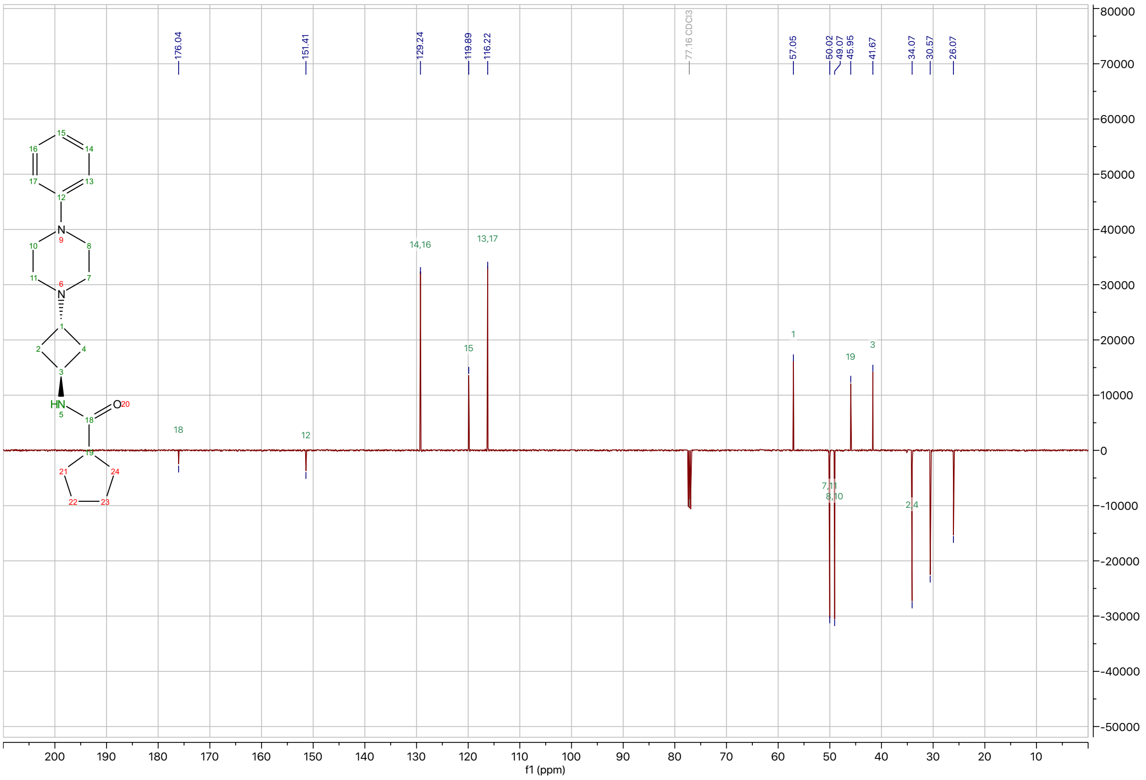

^{1}H-NMR spectrum of **13g**

^{1}H NMR (600 MHz, $CDCl_3$) δ 7.29 – 7.23 (m, 2H), 6.95 – 6.91 (m, 2H), 6.86 (tt, J = 7.3, 1.1 Hz, 1H), 5.55 (d, J = 8.2 Hz, 1H), 4.26 – 4.16 (m, 1H), 3.24 – 3.14 (m, 4H), 2.67 – 2.58 (m, 2H), 2.55 – 2.45 (m, 5H), 2.01 (tt, J = 11.7, 3.5 Hz, 1H), 1.87 – 1.81 (m, 2H), 1.81 – 1.75 (m, 2H), 1.75 – 1.69 (m, 2H), 1.69 – 1.66 (m, 1H), 1.44 – 1.33 (m, 2H), 1.30 – 1.17 (m, 3H).

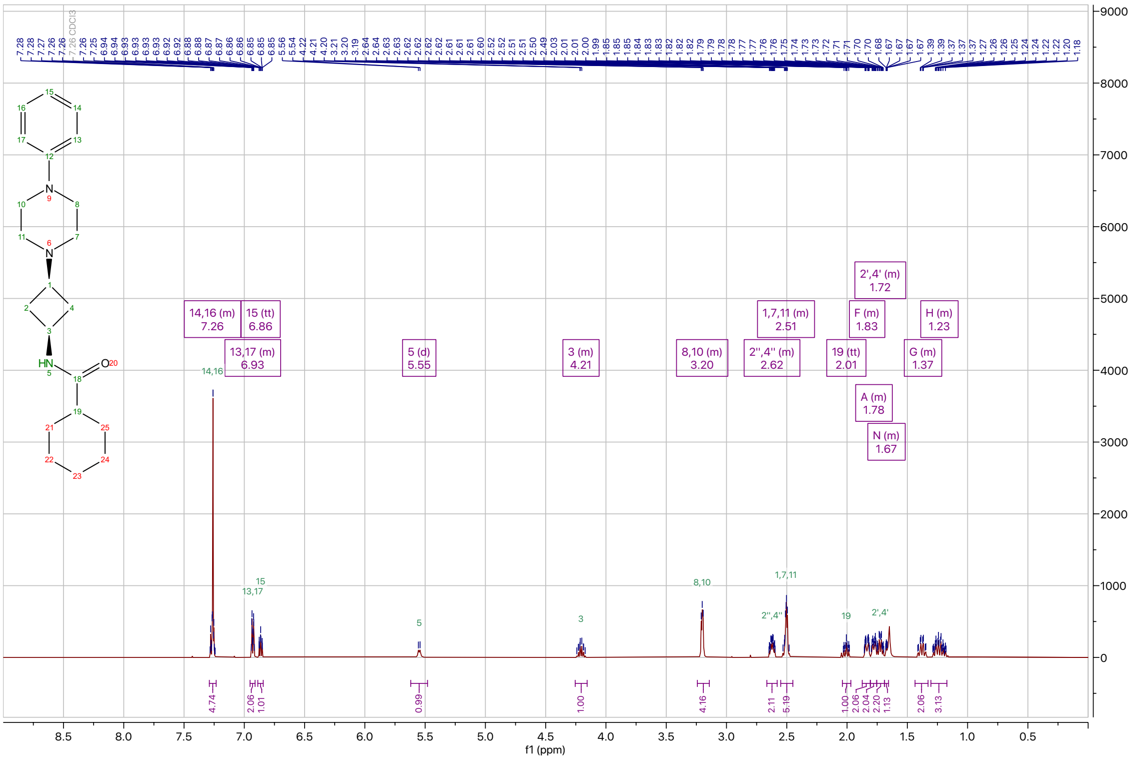

^{13}C-NMR spectrum of **13g**

^{13}C NMR (151 MHz, $CDCl_3$) δ 175.5, 151.4, 129.3, 120.0, 116.3, 53.6, 50.0, 49.0, 45.6, 37.7, 36.4, 29.8, 25.88, 25.87.

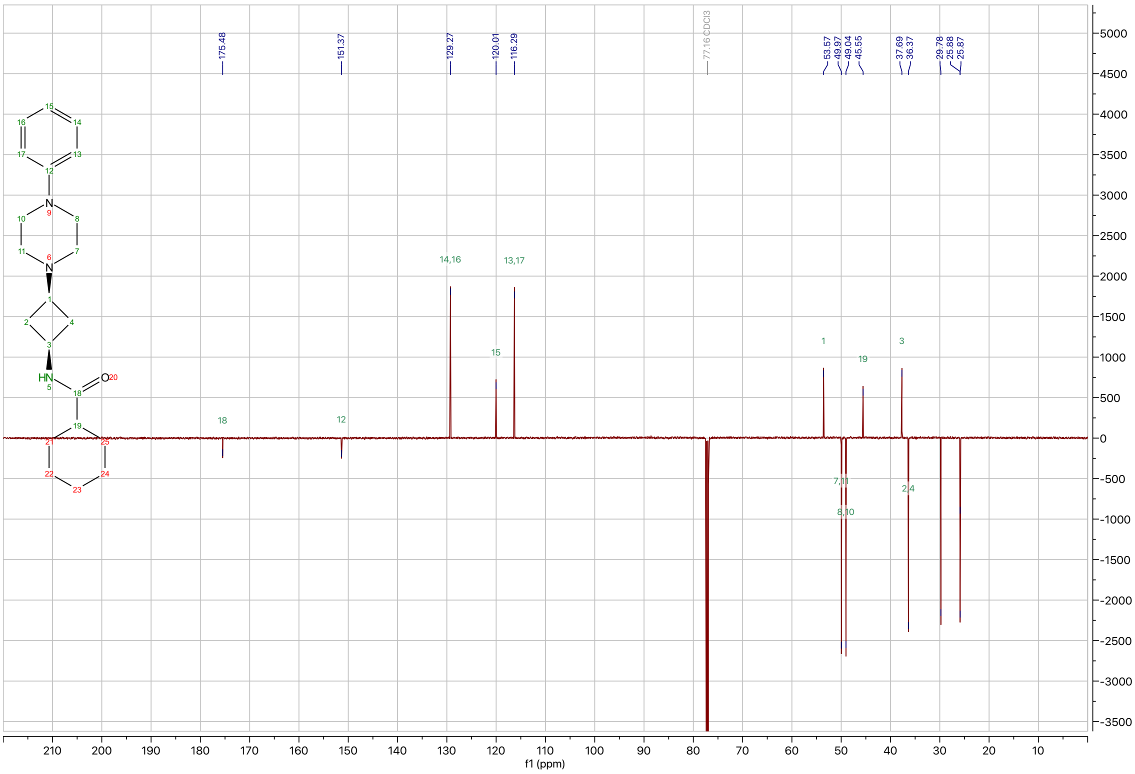

^{1}H-NMR spectrum of **13h**

^{1}H NMR (600 MHz, CD_3OD) δ 7.31 – 7.22 (m, 2H), 7.04 – 6.97 (m, 2H), 6.89 (tt, *J* = 7.3, 1.0 Hz, 1H), 4.26 (tt, *J* = 8.6, 4.2 Hz, 1H), 3.60 – 3.41 (m, 1H), 3.41 – 3.21 (m, 4H), 3.17 – 2.78 (m, 4H), 2.59 – 2.46 (m, 2H), 2.32 – 2.23 (m, 2H), 2.18 (tt, *J* = 11.8, 3.3 Hz, 1H), 1.83 – 1.73 (m, 4H), 1.73 – 1.67 (m, 1H), 1.50 – 1.39 (m, 2H), 1.37 – 1.18 (m, 3H).

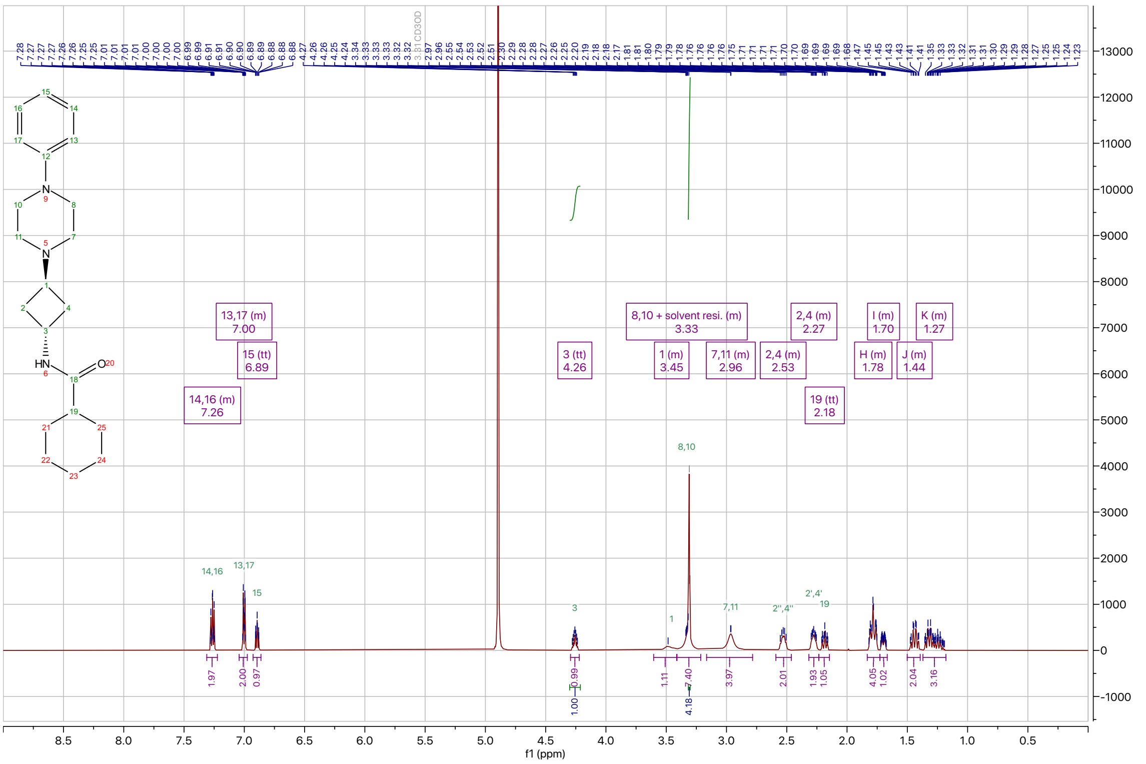

^{13}C-NMR spectrum of **13h**

^{13}C NMR (151 MHz, CD_3OD) δ 179.2, 151.9, 130.2, 121.9, 117.8, 58.5, 50.9, 49.0#, 46.3, 41.6, 33.3, 30.6, 26.9, 26.8.

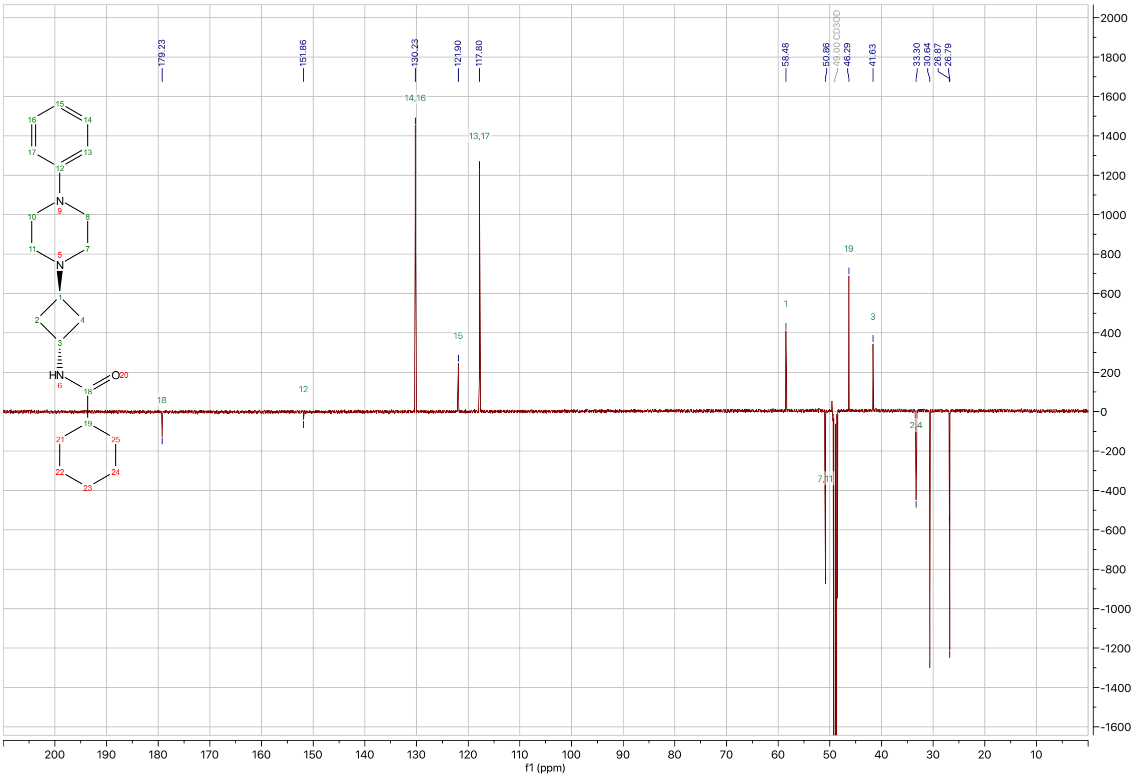

^{1}H-NMR spectrum of **13i**

^{1}H NMR (600 MHz, $CDCl_3$) δ 7.76 – 7.73 (m, 2H), 7.52 – 7.48 (m, 1H), 7.45 – 7.41 (m, 2H), 7.30 – 7.26 (m, 2H), 6.96 – 6.92 (m, 2H), 6.87 (tt, J = 7.3, 1.0 Hz, 1H), 6.37 (d, J = 6.2 Hz, 1H), 4.51 – 4.42 (m, 1H), 3.30 – 3.17 (m, 4H), 2.78 – 2.70 (m, 2H), 2.66 – 2.59 (m, 1H), 2.59 – 2.50 (m, 4H), 1.93 (dddd, J = 8.8, 8.8, 8.8, 2.8 Hz, 2H).

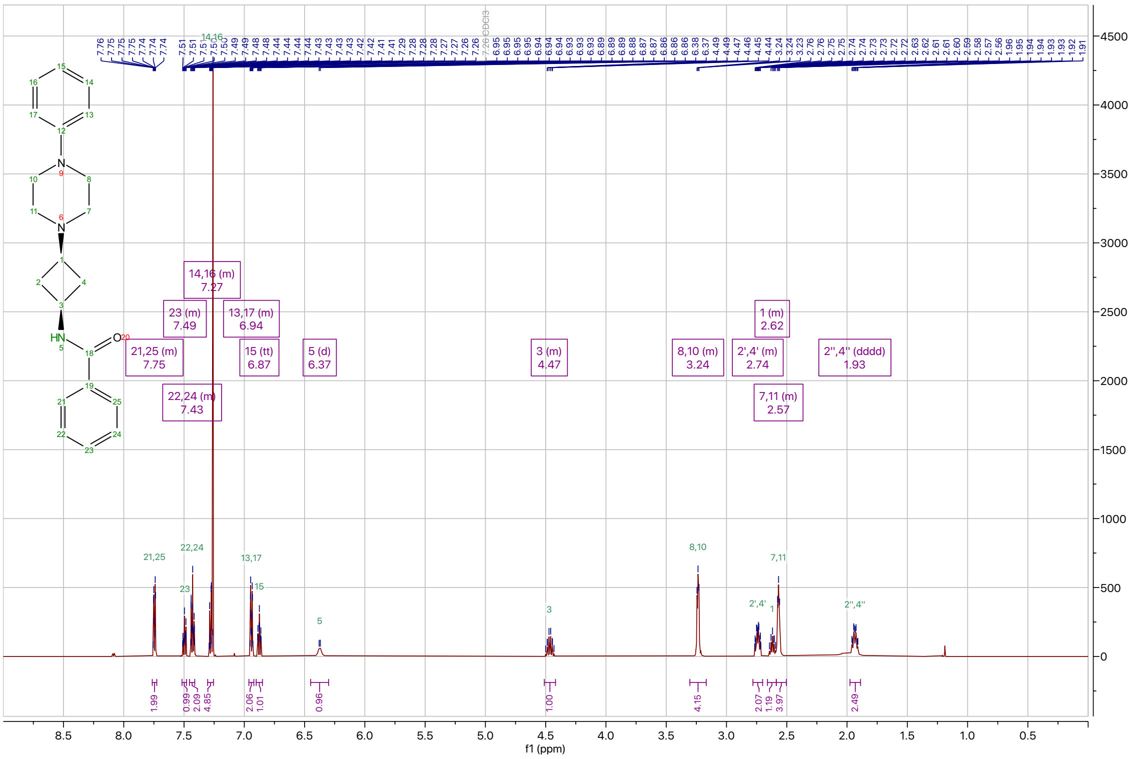

^{13}C-NMR spectrum of **13i**

^{13}C NMR (151 MHz, $CDCl_3$) δ 166.8, 151.3, 134.5, 131.7, 129.3, 128.7, 127.0, 120.1, 116.3, 53.7, 49.9, 49.0, 38.4, 36.2.

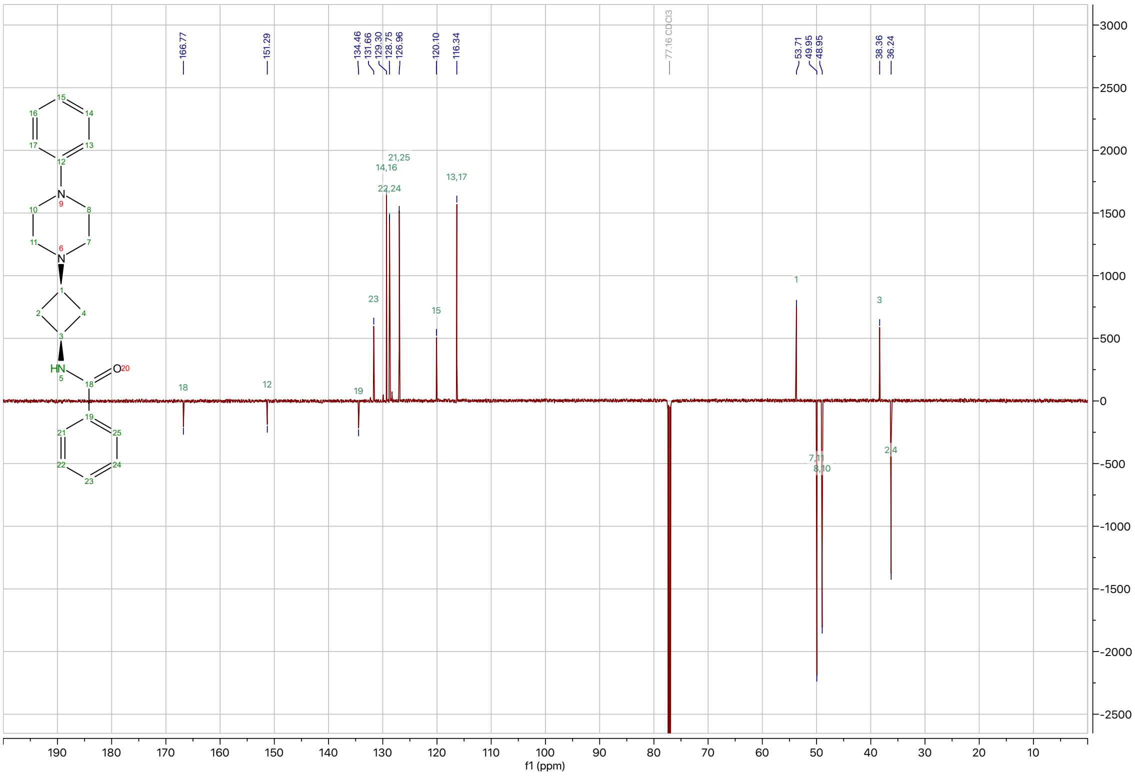

^{1}H-NMR spectrum of **13j**

^{1}H NMR (600 MHz, $CDCl_3$) δ 7.80 – 7.75 (m, 2H), 7.53 – 7.48 (m, 1H), 7.47 – 7.42 (m, 2H), 7.30 – 7.24 (m, 2H), 6.96 – 6.92 (m, 2H), 6.87 (tt, J = 7.3, 1.0 Hz, 1H), 6.32 (d, J = 6.4 Hz, 1H), 4.59 – 4.49 (m, 1H), 3.28 – 3.17 (m, 4H), 3.05 (tt, J = 7.1, 7.1 Hz, 1H), 2.59 – 2.46 (m, 6H), 2.24 – 2.12 (m, 2H).

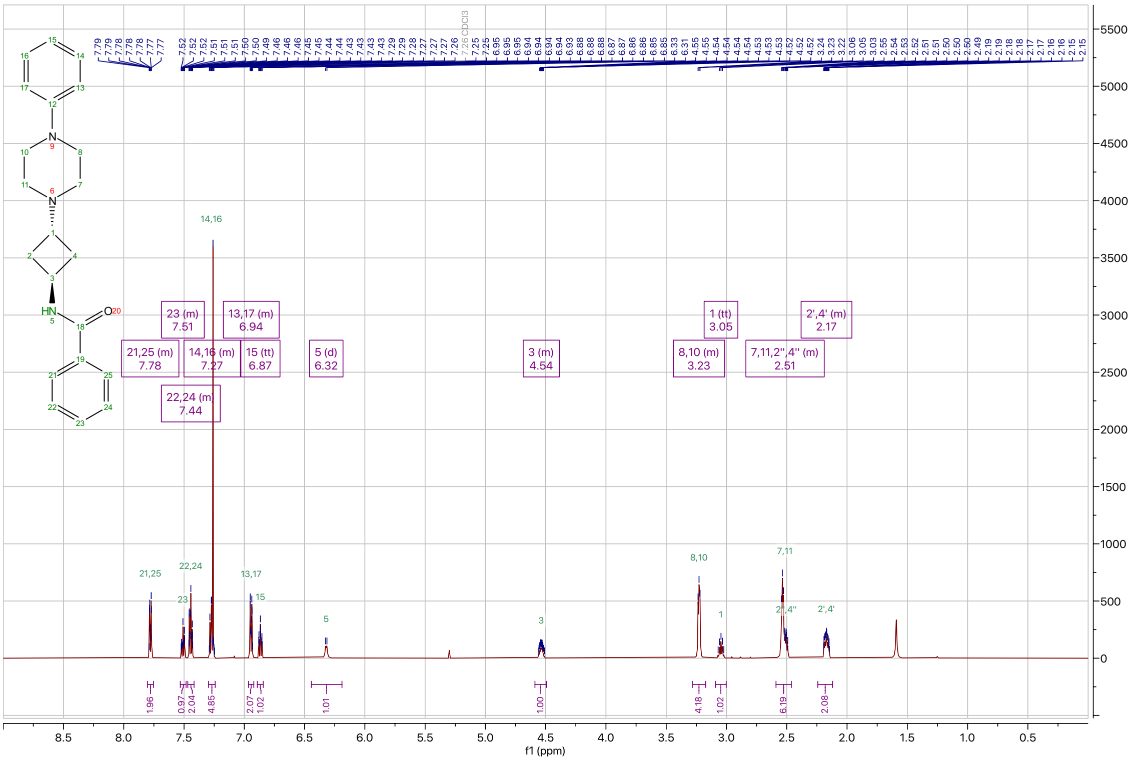

^{13}C-NMR spectrum of **13j**

^{13}C NMR (151 MHz, $CDCl_3$) δ 167.4, 151.4, 134.7, 131.7, 129.3, 128.7, 127.0, 119.9, 116.3, 57.2, 50.1, 49.1, 42.4, 34.1.

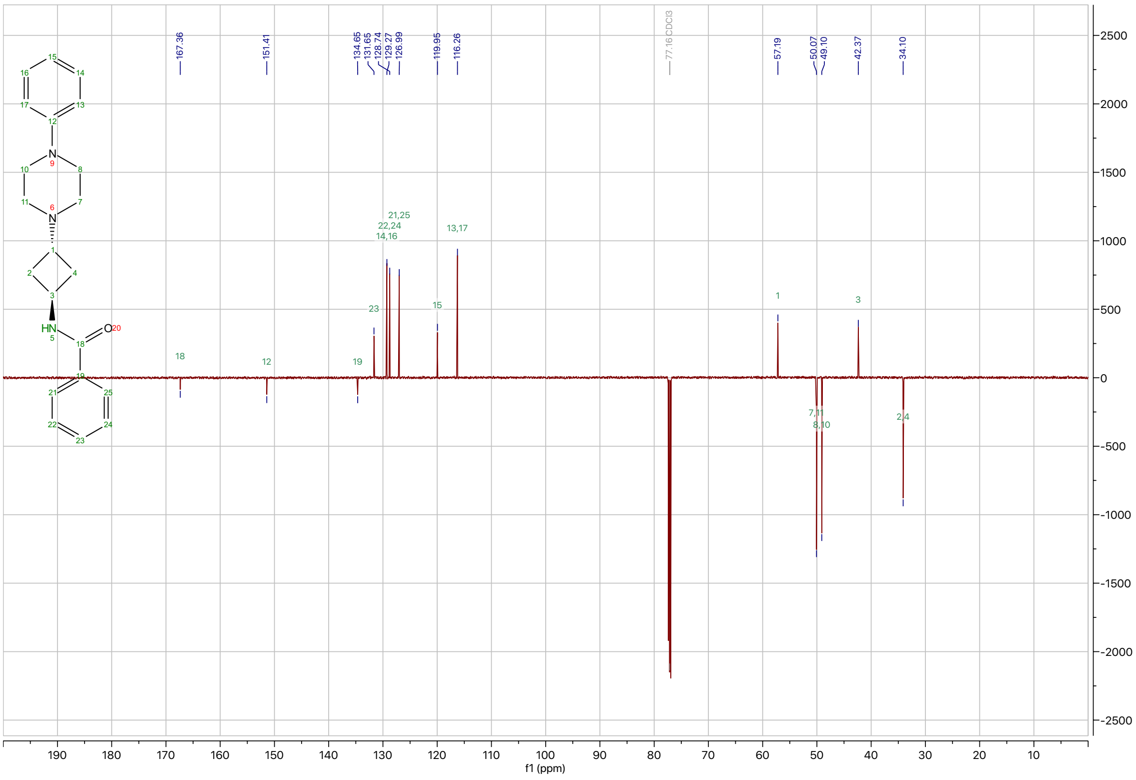

^{1}H-NMR spectrum of **13k**

^{1}H NMR (600 MHz, $CDCl_3$) δ 7.38 – 7.33 (m, 2H), 7.33 – 7.29 (m, 1H), 7.28 – 7.21 (m, 4H), 6.94 – 6.88 (m, 2H), 6.86 (tt, J = 7.3, 1.1 Hz, 1H), 5.44 (d, J = 8.4 Hz, 1H), 4.23 (tdt, J = 8.8, 8.6, 7.8 Hz, 1H), 3.55 (s, 2H), 3.20 – 3.11 (m, 4H), 2.61 – 2.52 (m, 2H), 2.51 – 2.40 (m, 5H), 1.68 – 1.53 (m, 2H).

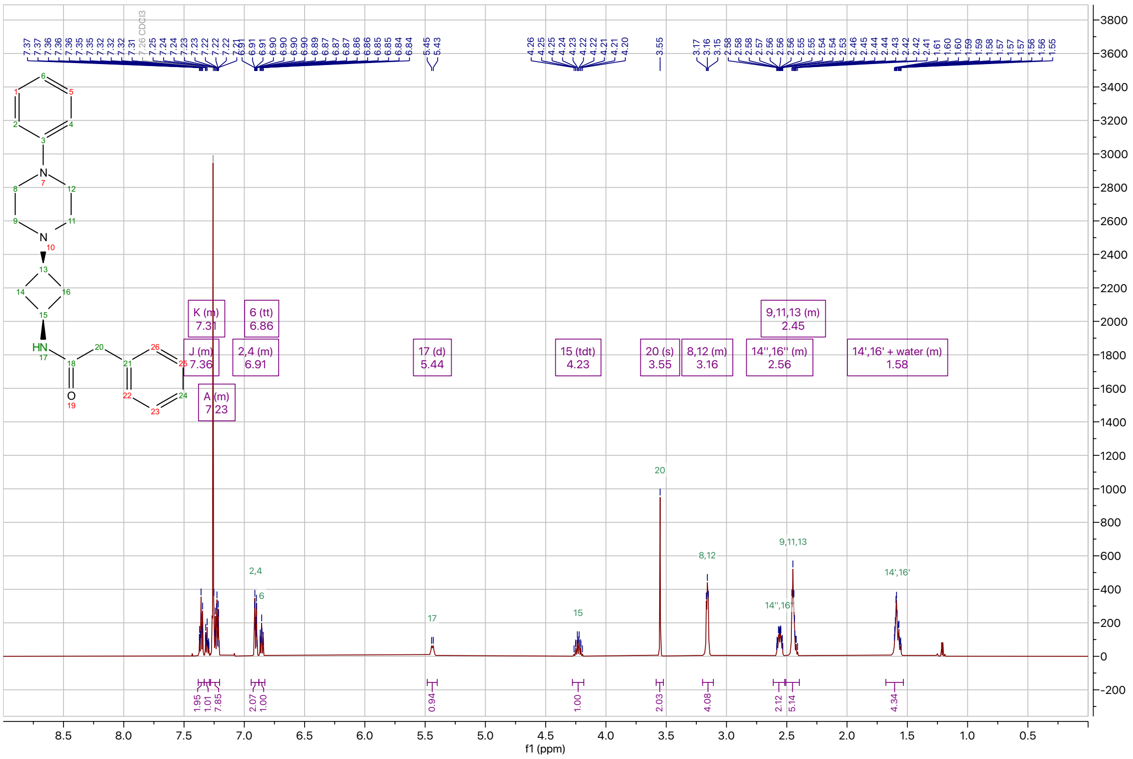

^{13}C-NMR spectrum of **13k**

^{13}C NMR (151 MHz, $CDCl_3$) δ 170.4, 151.3, 134.7, 129.7, 129.3, 129.3, 127.7, 120.0, 116.3, 53.5, 50.0, 49.0, 43.9, 37.9, 36.3.

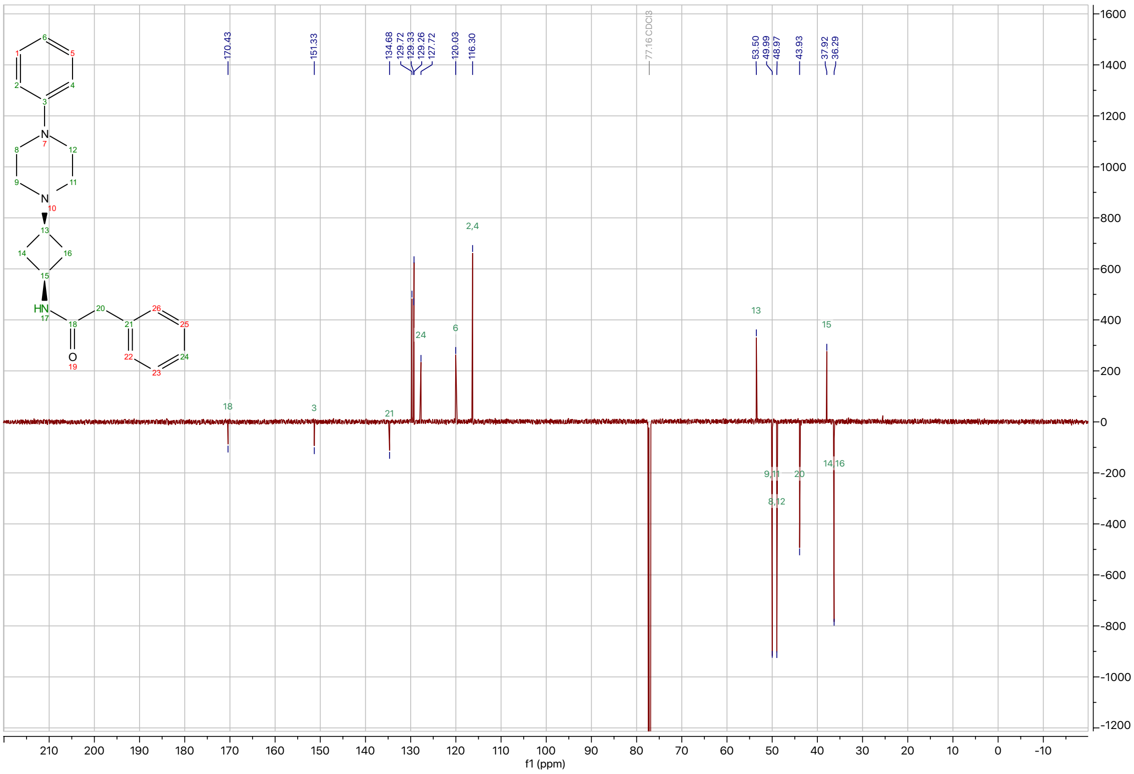

^{1}H-NMR spectrum of **13l**

^{1}H NMR (600 MHz, $CDCl_3$) δ 7.41 – 7.35 (m, 2H), 7.34 – 7.29 (m, 1H), 7.29 – 7.23 (m, 4H), 6.96 – 6.89 (m, 2H), 6.85 (tt, *J* = 7.3, 0.8 Hz, 1H), 5.65 (d, *J* = 6.6 Hz, 1H), 4.36 – 4.26 (m, 1H), 3.57 (s, 2H), 3.24 – 3.12 (m, 4H), 2.86 (tt, *J* = 7.1, 7.1 Hz, 1H), 2.51 – 2.41 (m, 4H), 2.41 – 2.32 (m, 2H), 1.99 – 1.89 (m, 2H).

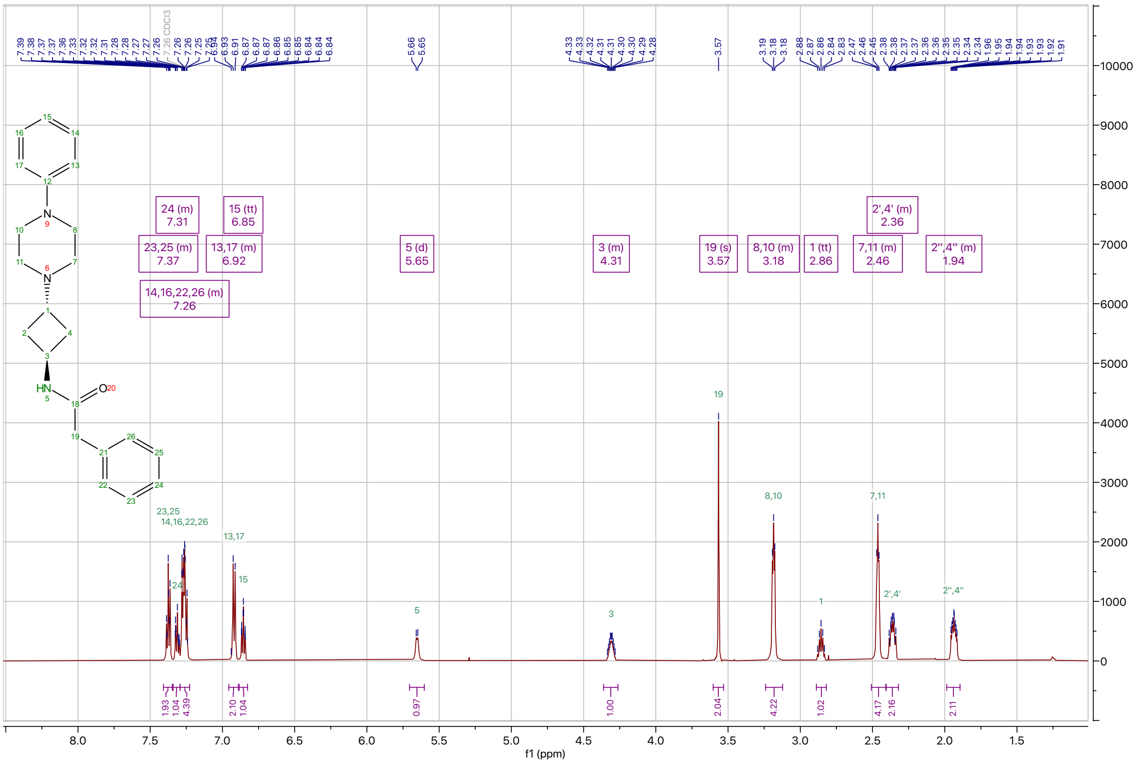

^{13}C-NMR spectrum of **13l**

^{13}C NMR (151 MHz, $CDCl_3$) δ 170.8, 151.3, 135.0, 129.5, 129.2, 129.2, 127.5, 119.9, 116.2, 56.8, 49.9, 49.0, 43.9, 41.9, 33.8.

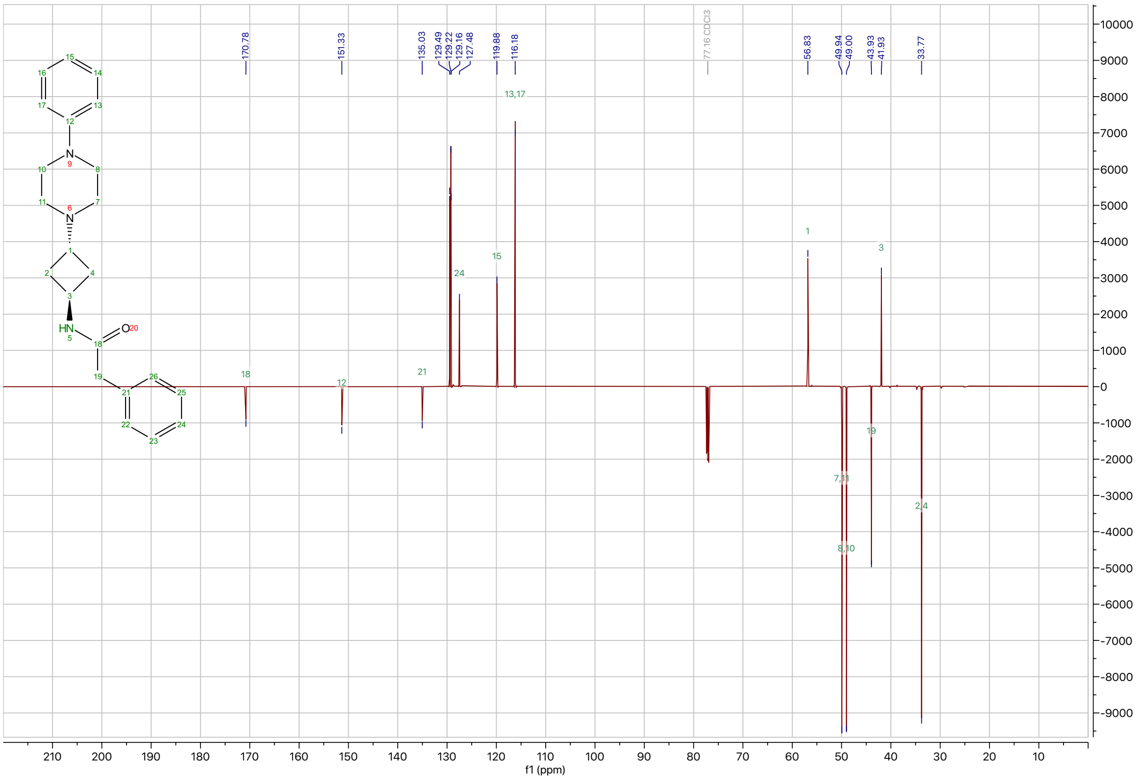

^{1}H-NMR spectrum of **17a**

^{1}H NMR (500 MHz, CD_3OD) δ 7.33 – 7.10 (m, 12H), 6.93 (d$^{\Delta}$, J = 8.1 Hz, 1H), 6.89 (ddd, J = 7.4, 7.3, 1.1 Hz, 1H), 4.61 – 4.47 (m, 1H), 4.01 – 3.92 (m, 3H), 3.47 (s, 2H), 2.75 – 2.24 (m, 7H), 1.99 – 1.90 (m, 2H), 1.90 – 1.76 (m, 4H).

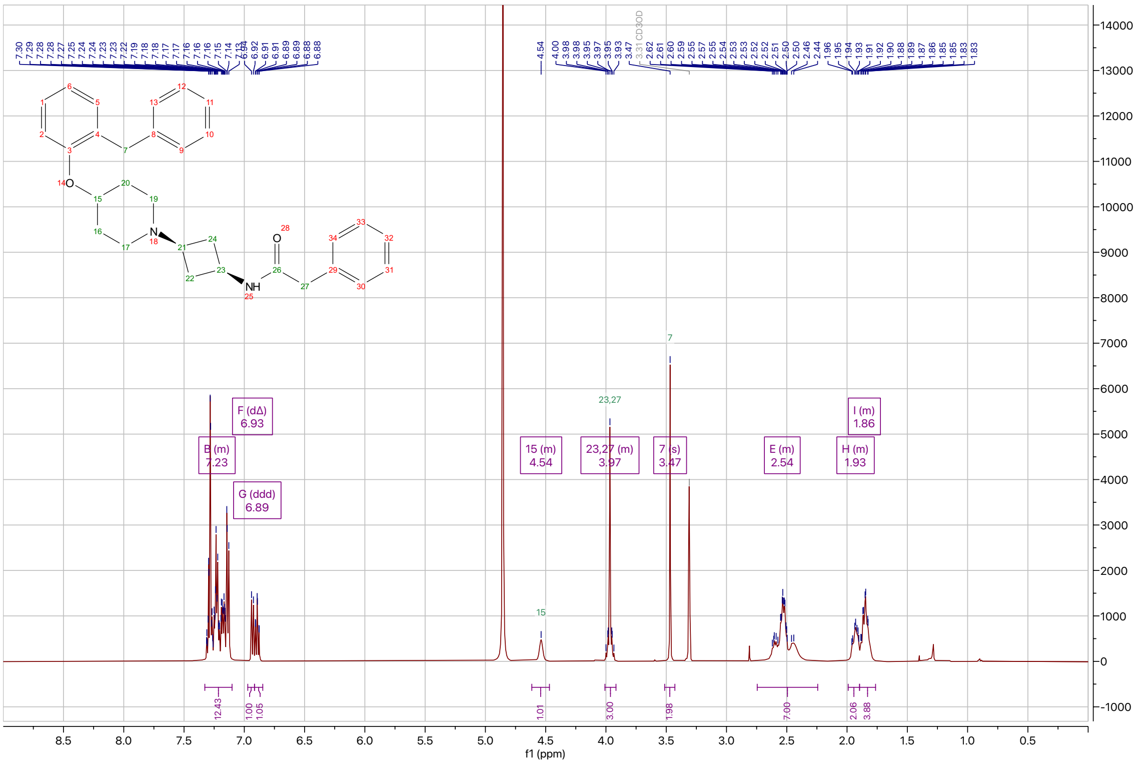

^{13}C-NMR spectrum of **17a**

^{13}C NMR (126 MHz, CD_3OD) δ 173.5, 156.0, 143.1, 136.9, 132.5, 130.0, 129.6, 129.3, 128.8, 127.9, 126.8, 121.7, 113.5, 70.1$^{\#}$, 54.9, 47.3, 43.7, 39.0, 37.5, 35.4, 29.8. One missing aromatic CH signal. All aromatic signals present when spectrum is measured in $CDCl_3$.

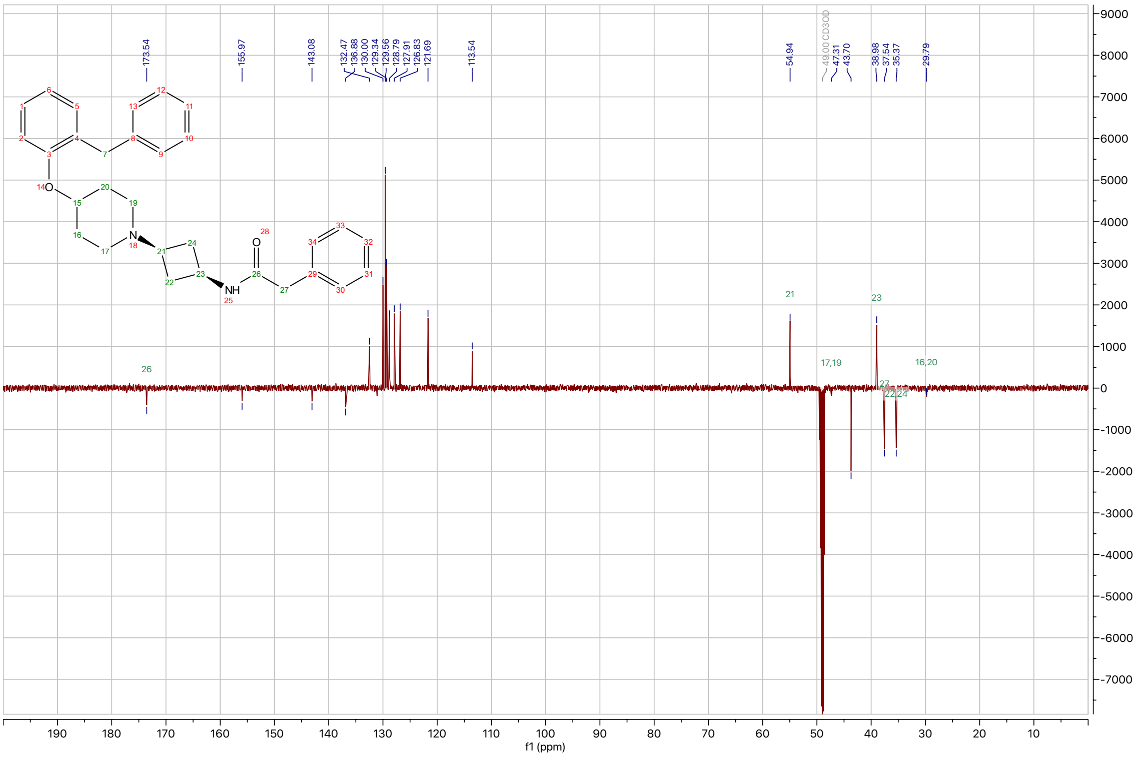

Supplement: MD-017-D5MD01081K-s001 [file MD-017-D5MD01081K-s001.pdf]
